# Upwind terrestrial influences on soil moisture variability across South America

**DOI:** 10.1038/s41467-026-75637-x

**Published:** 2026-07-24

**Authors:** Feini Huang, Shijie Jiang, Wei Shangguan, Gustau Camps-Valls, Alexander Winkler, Wantong Li, Gregory Duveiller, Christian Reimers, Wenli Zhao, Markus Reichstein, Yongjiu Dai

**Affiliations:** 1 School of Atmospheric Sciences, Sun Yat–Sen University, Zhuhai, China; 2 Department of Biogeochemical Integration, Max Planck Institute for Biogeochemistry, Jena, Germany; 3 ELLIS Unit Jena, Jena, Germany; 4 Guangdong Province Key Laboratory for Climate Change and Natural Disaster Studies, Sun Yat–Sen University, Zhuhai, China; 5 Southern Marine Science and Engineering Guangdong Laboratory (Zhuhai), Zhuhai, China; 6 Image Processing Lab (IPL), Universitat de València, Valencia, Spain; 7 Department of Environmental Science, Policy and Management, UC Berkeley, Berkeley, CA USA; 8 Climate and Ecosystem Sciences Division, Lawrence Berkeley National Laboratory, Berkeley, CA USA; 9 Department of Earth and Environmental Engineering, Columbia University, New York, NY USA

**Keywords:** Hydrology, Atmospheric dynamics, Hydrology

## Abstract

Soil moisture across South America exhibits coherent spatial patterns shaped by upwind terrestrial conditions, yet their downwind propagation remains poorly quantified. We develop an observation-driven framework integrating atmospheric transport trajectories with deep learning to estimate vegetation’s contribution to downwind soil moisture. The transport-informed model outperforms a local-only baseline, with upwind information contributing 67% of predictive power, of which vegetation explains 15%. This vegetation-mediated influence is enhanced over agricultural zones, forming hotspots aligned with land-cover change and drought exposure. The cross-regional influence is organized along major transport corridors and varies non-linearly with climate. In humid forests, downwind moisture supply is highly sensitive to vegetation change, with canopy degradation associated with reduced soil moisture; in semi-arid regions, initial greening is associated with increases, but further greening weakens or reverses the effect. These results quantify vegetation–soil moisture teleconnections, with implications for land–atmosphere coupling in Earth system models and hydrological consequences of land-use and climate change.

## Abstract (web summary)

The study applies deep learning and moisture tracking and finds that Amazonian vegetation shapes soil moisture thousands of kilometers downwind across South America, sustaining distant farmland and showing how forest loss could worsen drought risk.

## Introduction

Soil moisture is a key regulator of terrestrial ecosystems and climate, with its variability carrying important implications for water security^1^, agricultural productivity^2^, ecosystem stability^3^, and climate extremes^4^. At local scales, vegetation modulates soil moisture variability through land–atmosphere feedbacks^1^, surface energy balance^5^, and precipitation partitioning^6–8^. Crucially, evapotranspiration (ET) is the dominant source of recycled atmospheric moisture^9,10^ and contributes roughly 40% of continental precipitation^11,12^. Recent studies indicate that vegetation–soil moisture interactions can extend far beyond local scales, with effects carried by atmospheric moisture transport over hundreds to thousands of kilometers^13,14^. On larger scales, it has been proposed that vegetation can generate pressure gradients that draw oceanic moisture inland (the “biotic pump” hypothesis)^15^. These mechanisms suggest that vegetation acts not only as a local regulator of water balance but also as a continental-scale organizer of hydrological connectivity that may link distant regions through atmospheric moisture transport. For example, higher leaf area index (LAI; a proxy for vegetation dynamics) has been linked to enhanced downwind soil water availability^16^, especially during dry seasons^17^. Despite case studies related to downwind effects^16,18,19^, we still lack a systematic framework to quantify how upwind vegetation dynamics shape soil moisture variability downwind, how these effects vary across seasons, and how they depend on climate. This gap limits our understanding of land–atmosphere feedbacks and how ecosystems sustain water availability across regions^18–21^. This limitation is particularly consequential under climate change and rapid land-use change, as vegetation loss in key source regions (e.g., Amazon) may alter soil moisture regimes in downwind regions (e.g., Brazilian agricultural zones) through atmospheric moisture transport.

The need for such a framework is clear in South America, where the Amazon rainforest serves as a major source of continental moisture and has experienced decades of deforestation. Observations indicate that a 1% decrease in Amazon forest cover reduces local precipitation by 0.25 mm/month at a 200-km scale^22^, and moisture tracking studies show that Amazonian ET sustains rainfall hundreds of kilometers away in agricultural regions such as southeastern Brazil and the La Plata basin^23^. However, the direction and strength of vegetation-climate linkages remain uncertain. Models and observations often disagree even on the sign of vegetation’s remote impact on precipitation^24–26^, reflecting data gaps and incomplete representations of land–atmosphere feedbacks^22,26^. Soil moisture integrates surface and subsurface processes and retains memory on multi-week timescales^27,28^, making it a critical variable for understanding how remote vegetation signals manifest in land–atmosphere interactions. Existing Lagrangian and Eulerian approaches^29,30^ can track moisture transport^31^, identify source regions^32^, and map atmospheric corridors^33^, but they focus primarily on precipitation. Consequently, vegetation–soil moisture coupling remains largely unrepresented, outside of fully coupled climate models that often carry large uncertainties^27,28^. These limitations motivate a complementary observation-driven strategy that uses transport information to understand vegetation–soil moisture teleconnections without relying on climate model assumptions.

Here, we develop a framework that integrates deep learning with atmospheric moisture trajectories to quantify the remote influence of vegetation on soil moisture anomalies (SMA) across South America (Methods; Figs. S1–S3). This framework disentangles terrestrial drivers, including vegetation, climate, and surface properties, whose signals are carried by upwind moisture transport, allowing us to attribute downwind soil moisture variability to specific upwind land surface processes. We introduce two metrics: source influence, the contribution of local LAI anomalies to downwind SMA; and sink sensitivity, the response of local SMA to upwind LAI anomalies. We apply this framework to quantify how Amazonian vegetation dynamics shape downwind SMA across the continent, assess how these linkages align with atmospheric trajectories, and characterize how their strength and direction vary with climate. The results identify regions where hydrological stability is most sensitive to ongoing vegetation change and provide a basis for assessing drought risk, land-use impacts, and the potential downwind consequences of vegetation loss.

## Results

### Upwind vegetation signals improve soil moisture prediction

**Fig. 1:**
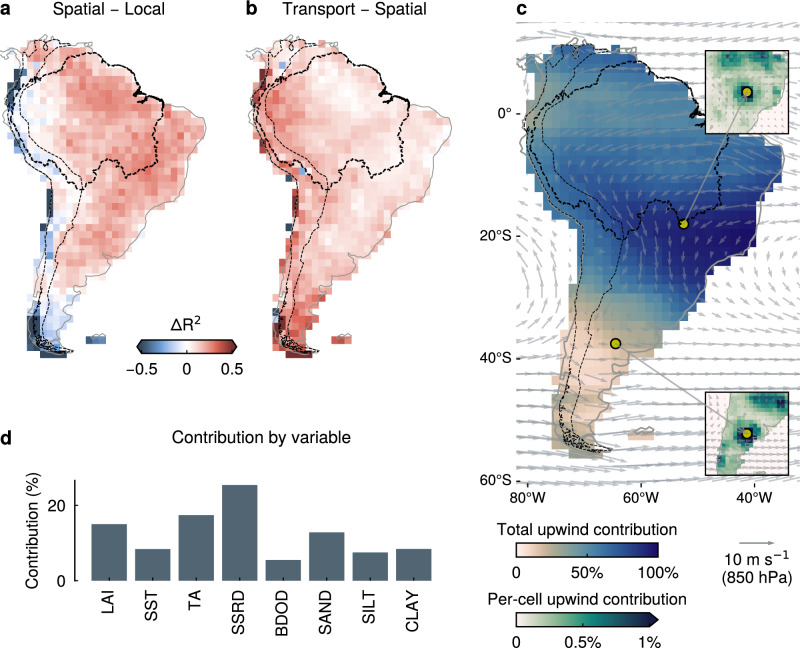
Performance gains of the transport-informed model and predictive attribution from land surface and near-surface atmospheric inputs. **a** Spatial-context gain (ΔR²) from the local-only model; positive values (red) indicate that adding non-directional spatial context improves skill. **b** Transport-informed gain (ΔR^2^) from spatial-context model; positive values indicate the additional gain from transport-informed directionality. Panels a and b share the ΔR^2^ color scale. **c** Total upwind contribution: for each target grid cell, the fraction of predictive attribution originating from all upwind terrestrial grid cells, computed as the summed absolute attribution over upwind cells normalized by the summed absolute attribution over all input grid cells in its 21 × 21 window (a value of 100% indicates complete dependence on upwind information). Upwind cells are identified with UTrack^34^ (Methods); predictive attribution is from expected gradients^38^ (Methods). The two insets show, for two representative target cells (yellow points), how this attribution is distributed across the surrounding source cells; colors give the per-cell contribution (% of that target cell’s total attribution), so the value mapped in **c** equals the sum over the upwind cells shown in each inset. Gray arrows are climatological 850 hPa wind vectors from ERA5 (2001–2018). **d** Contribution by variable (%), showing the share of total upwind predictability assigned to each terrestrial variable (summing to 100% across all eight variables) in the transport-informed model. It is estimated by the fraction of total absolute attribution over all upwind grid cells assigned to each variable. Variables include leaf area index (LAI), sea surface temperature (SST), air temperature at 2 m (TA), surface downward shortwave radiation (SSRD), bulk density (BDOD), and sand (SAND), silt (SILT), and clay (CLAY) fractions. The geographic base map in (**a**–**c**), including coastlines and country borders, is generated using the public-domain Natural Earth dataset via Python’s Cartopy library.

Soil moisture variability across South America is better captured when upwind vegetation information is incorporated. We evaluate this by comparing three models (Methods and Supplementary Information), including a local-only model that uses only local conditions, a spatial-context model with non-directional neighbors, and a transport-informed model guided by UTrack^34^ climatological moisture-trajectory information (Methods; Fig. S3). The transport-informed model achieves a cross-validated mean R^2^ of 0.87 across grid cells and seasons (Fig. S4), substantially outperforming the spatial-context (0.70) and local-only (0.64) models (Fig. 1a, b), with improvements observed in approximately 95% of the grid cells. Performance gains are pronounced downwind of the Amazon and along the eastern Andes, in alignment with transport pathways such as the South American low-level jet (SALLJ)^35^, which transports Amazonian moisture southward toward agricultural zones^35–37^. The model performance remains consistently high across regions spanning the full range of LAI anomaly mean and variance (Fig. S5), indicating that upwind information improves predictability even in regions with limited local vegetation variability. Agreement across independent soil moisture products indicates that the result is not dataset-specific (Fig. S6).

To identify which inputs drive this skill, we obtain predictive attribution using expected gradients^38^ (Methods). The predictive attribution quantifies the contribution of each terrestrial input to SMA in a target grid cell. Inputs from upwind grid cells, as defined by UTrack, account for 67% of the total predictive attribution (Fig. 1c), and are consistent with the climatological atmospheric circulation pattern^35,39^ (Fig. S7). Within this upwind contribution, LAI accounts for 15% of the upwind attribution on average (Fig. 1d), alongside contributions from upwind air temperature and shortwave radiation (for spatial patterns, see Fig. S8). Of these, shortwave radiation contributes the largest share, consistent with its role in driving ET and surface energy partitioning; together, these variables control transpiration, surface energy partitioning, and boundary-layer mixing, which jointly affect atmospheric moisture content and transport efficiency^40,41^. Attribution patterns are consistent across interpretation methods (Fig. S9) and model initializations (Fig. S10).

### Source–sink spatial structure of vegetation–soil moisture teleconnections

**Fig. 2:**
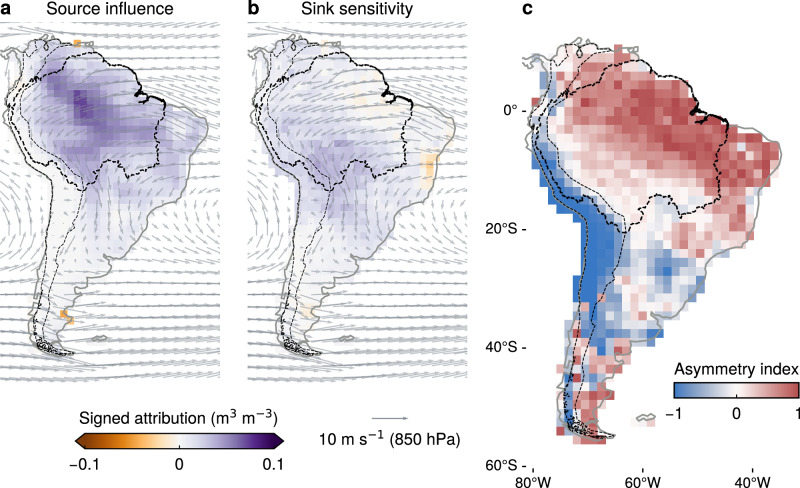
Spatial structure of directional vegetation-soil moisture teleconnections across South America. **a** Source influence, which quantifies the outgoing effect of local leaf area index (LAI) anomalies on downwind soil moisture anomalies (SMA), averaged over its downwind target cells (2001–2018; Methods). **b** Sink sensitivity, which quantifies the incoming effect of upwind LAI anomalies on local SMA, averaged over all upwind source cells (2001–2018; Methods). Positive values (purple) indicate that greening is associated with wetter downwind soil; negative values (orange) indicate the opposite. Panels a and b share the same color scale. **c** Asymmetry index (Methods; Fig. S14), computed from the magnitudes |source influence| and |sink sensitivity|. Values range from +1 (predominantly source/outgoing) through 0 (balanced) to −1 (predominantly sink/incoming), which reflects whether vegetation at a cell exerts a stronger influence on downwind soil moisture than it receives from upwind. Gray arrows in **a**–**b** indicate climatological 850 hPa wind vectors from ERA5 (2001–2018). The geographic base map in (**a**–**c**), including coastlines and country borders, is generated using the public-domain Natural Earth dataset via Python’s Cartopy library.

To trace the spatial footprints of these upwind vegetation effects (Fig. 1), we map two complementary metrics: source influence and sink sensitivity. These metrics leverage the model’s predictive attributions to capture the directionality of land–atmosphere pathways, allowing us to distinguish regions that export moisture signals from those that receive them (Fig. 2a, b; Methods; Fig. S11). The maps reveal a predominantly positive and spatially coherent source–sink structure. The influence a cell exerts downwind and the influence it receives from upwind are, however, often unequal (Fig. 2c; Methods; Fig. S14). The western and northern Amazon emerge as dominant source regions (Fig. 2a) and exhibit strongly positive asymmetry values (Fig. 2c). This pattern is consistent with moisture tracking studies showing that Amazonian ET supports rainfall in southeastern Brazil, the La Plata basin, and the eastern Andes^41–44^. These source regions align with major atmospheric transport corridors, particularly the SALLJ, and export moisture toward areas that sustain roughly 80% of Brazil’s agricultural production^45^. Areas of strong source influence overlap southern Amazonia, where deforestation rates are high (Fig. S15), suggesting that forest loss there could coincide with greater soil water vulnerability.

Regions of high sink sensitivity are concentrated in southeastern South America (Fig. 2b), corresponding to negative asymmetry indexes (Fig. 2c), including agricultural states such as Mato Grosso and Paraná. In these areas, moisture recycled from the Amazon supplies up to one-third of rainfall^46^. On average, in a typical month 55% of cropland cells are LAI-dominant, i.e., the LAI-derived sink sensitivity exceeds the median of the eight input variables, indicating that upwind LAI is the leading driver of cropland soil moisture sensitivity (Methods). Additionally, upwind LAI anomalies account for 15% of total predictive skill, with the largest gains during the growing season (Fig. S16). About 60% of identified drought events coincide with positive sink sensitivity, consistent with partial moderation of severity in some cases (Fig. S17; Methods and Supplementary Information). If the large-scale asymmetry (Fig. 2c) persists or intensifies, these far-downwind sink regions may face growing irrigation demand and higher drought exposure, particularly under enhanced self-propagating drought processes^47^. These results indicate that vegetation–soil moisture teleconnections via atmospheric moisture transport can modulate downwind soil water availability, with the largest benefits during growing periods (Fig. S18). Empirical and modeling evidence further links reductions in Amazonian ET to decreased precipitation and crop yields in these farming areas^48^, highlighting the dependence of southeastern Brazil and the La Plata basin on moisture imports from Amazonian source regions.

The observed spatial structure is governed by atmospheric moisture transport dynamics. The gradient of the asymmetry index from the coastal Amazon to the continental interior is consistent with the “biotic pump” hypothesis^15^, which proposes vegetation-generated pressure gradients that draw moisture inland. Widespread positive teleconnections indicate that where vegetation greening is associated with increased soil moisture downwind, additional ET can augment moisture export and benefit regions downwind. Negative patches suggest situations in which enhanced ET strengthens local precipitation recycling at the expense of downwind moisture transport^16,17^. These negative effects can be further amplified by vegetation-induced reductions in albedo and increases in surface roughness, which intensify turbulent mixing and convection^49^.

Our continent-scale analysis shows pronounced spatial heterogeneity. Positive source-sink linkages are widespread across South America, but local hydroclimatic controls dominate much of the southern and southwestern regions, where both source influence and sink sensitivity are weak. Beyond these direct linkages, the results raise the possibility of cascading land–atmosphere effects, where regions receiving Amazonian moisture can act as secondary sources, transmitting vegetation-induced soil moisture anomalies further downwind (Figs. S19 and S20; Supplementary Information). This cascading structure implies that vegetation change in one part of the continent can propagate impacts on hydrological cycles^50,51^ and water security^52^.

### Climatic regimes of non-linear vegetation–soil moisture teleconnections

**Fig. 3:**
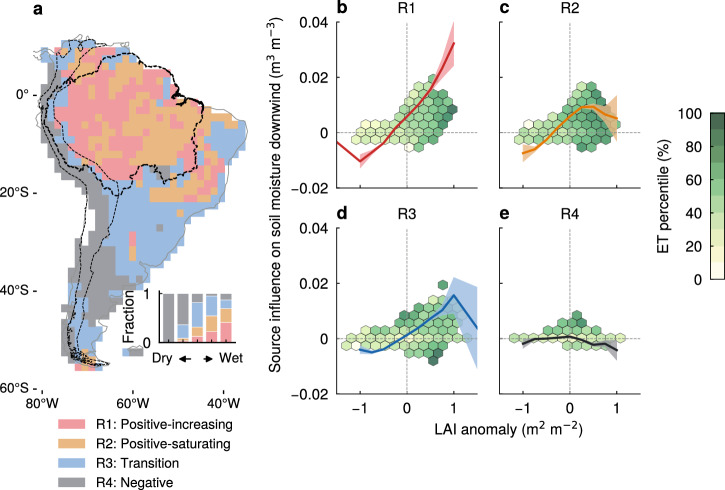
Non-linear regimes of vegetation influence on downwind soil moisture. **a** Spatial distribution of the four climatic regimes across South America, reflecting the consistency and shape of the downwind soil moisture response to upwind vegetation changes. Regimes are defined by whether the source influence remains predominantly positive ( > 70% of months) and by its post-breakpoint trend (Methods; see Fig. S21 for robustness across thresholds). White areas were excluded from the classification (hyper-arid cells, or cells with no statistically resolved coupling; Methods). The inset shows the fractional composition of the four regimes across five aridity classes (displayed from left to right) defined by the precipitation-to-potential evapotranspiration ratio, P/PET: hyper-arid ( < 0.05), arid (0.05–0.2), semi-arid (0.2–0.5), dry sub-humid (0.5–0.65), and humid ( > 0.65). **b**–**e** Relationship between upwind LAI anomalies (m^2^ m^−2^) and source influence on downwind soil moisture (m^3^ m^-3^) for each regime (R1–R4; color matches panel a). Solid lines show the regime-mean source influence within bins of LAI anomaly, and shaded bands the 95% confidence intervals; gray dashed lines mark zero. Hexagon bins are colored by the evapotranspiration (ET) percentile (calculated from the distribution of ET values within each grid cell) and require at least five grid cells per bin. The geographic base map, including coastlines and country borders, is generated using the public-domain Natural Earth dataset via Python’s Cartopy library.

Having mapped continental-scale patterns of vegetation–soil moisture teleconnections, we next examine how vegetation’s source influence varies non-linearly across background climates. We group the grid cells into four regimes (R1–R4; Fig. 3a) based on two criteria: 1) how often the upwind influence remains positive over time, and 2) how the downwind response changes after the non-linear coupling reaches its breakpoint (Methods). The regime patterns are robust across a physically meaningful range of persistence thresholds (Fig. S21). Their spatial distribution aligns with a gradient of moisture availability represented by the aridity index, indicating that background climate constrains vegetation’s downwind effects (Fig. 3a, inset). In each regime, we additionally color the response by the local ET percentile (relative to each cell’s own ET distribution; Fig. 3b–e), which links the strength of source influence to the prevailing ET state.

R1 and R2 dominate the humid Amazon basin, suggesting these regions play a generally positive role as moisture sources for remote regions. In R1, prevalent in the central and western Amazon, source influence is positive in more than 70% of months (*p* < 0.05; Binomial test; Methods). Increases in LAI are associated with nearly linear gains in downwind soil moisture once LAI anomalies rise above a low threshold (~−1.0 m^2^ m^−2^) (Fig. 3b); the highest source influence coincides with high-ET conditions, consistent with vigorous ET supplying moisture for downwind export. R2 spans the southern Amazon-Cerrado transition and follows a similar pattern until positive LAI anomalies exceed a moderate level (~+0.5 m^2^ m^−2^), after which source influence weakens (Fig. 3c). Although ET remains high in this range, the source influence no longer increases, indicating that additional evapotranspired moisture is increasingly retained locally, consistent with increasing water limitation in this transitional zone. R1 regions maintain higher terrestrial water availability than R2 (Fig. 3a, inset), supporting sustained ET during rapid vegetation growth.

R3 and R4 occur in semi-arid lowlands and highlands, where the sign of source influence can shift from positive to negative, suggesting a growing dominance of local moisture recycling over downwind export. R3, covering much of the La Plata basin and nearby croplands, shows a transitional response, where initial greening is associated with increased downwind soil moisture, but further greening yields diminishing returns and an eventual decline in the moisture available for atmospheric transport (Fig. 3d). R4 (Fig. 3e), concentrated in the Andes and Brazilian Highlands, shows weak source influence and a predominantly negative association, consistent with strong local recycling mediated by high-elevation thermal structure and topographic effects^53–55^. Here, varying ET percentiles are not associated with significant changes in source influence.

This regime framework is directly relevant for land-use change. Current deforestation hotspots overlap the positive moisture-export regimes (R1 and R2) in southeastern Amazonia (Fig. S15). Continued forest loss via deforestation or climate-driven dieback can alter these source characteristics, reducing Amazonian moisture export, weakening continental moisture connectivity, and increasing the exposure of downwind agricultural regions to water scarcity.

## Discussion

Our study provides observation-based, trajectory-informed evidence that Amazonian vegetation dynamics modulate soil moisture regimes hundreds to thousands of kilometers downwind along major atmospheric pathways. While previous studies suggested such teleconnections^37,43,44^, their strength is still difficult to quantify given persistent model-observation inconsistencies. By integrating directional atmospheric moisture trajectories within an observation-driven framework, the model outperforms alternatives without directional context (Fig. 1) and supports directional attribution of soil moisture variability to upwind vegetation. Beyond improved performance, the approach maps a systematic continent-scale source-sink architecture (Fig. 2) and shows that vegetation’s downwind influence operates through distinct, climate-dependent, and non-linear regimes (Fig. 3). This perspective is consistent with recent evidence that land-cover change can exert remote hydroclimatic impacts through atmospheric transport, with effects that depend on spatial scale and may even reverse seasonally, as reported for deforestation^17^. Building on these insights, our analysis provides a pathway to assess how land-use change, such as large-scale deforestation, may alter soil moisture conditions in downwind regions. Such non-local impacts are particularly relevant for anticipating consequences for agricultural productivity and food security far from the location of land surface change.

The spatial organization and non-linearity of these teleconnections matter for agriculture, drought risk, and land-use planning. Vegetation in the western and northern Amazon sustains soil moisture in distant agricultural zones, such as southeastern Brazil (Fig. 2b). For South American croplands, on average 55% of cells are LAI-dominant in a given month (i.e., the LAI-derived sink sensitivity exceeds the median across the eight input variables), especially in Mato Grosso and Paraná, identifying upwind vegetation as a leading contributor to their soil-moisture sensitivity (Fig. S16). More than 60% of drought events coincide with a positive sink sensitivity (Fig. S17), consistent with partial moderation of severity in some cases. However, the benefits are not uniform and depend on the background climate. Humid rainforests robustly support downwind moisture export (Fig. 3b), whereas teleconnection strength saturates (Fig. 3c) and can reverse in transitional or semi-arid regions (Fig. 3d), where local recycling can dominate over downwind transport. This asymmetry (Fig. 2c) indicates a critical vulnerability. Anthropogenic land-use change, which widely triggers severe LAI declines through deforestation and land degradation, especially in humid regions (R1 and R2 in Fig. 3a), threatens to disrupt these moisture supply regimes. Particularly if land-use change-driven forest loss exceeds ~40% in transitional zones^56^, the associated persistent drop in LAI could shift moisture exporting regions toward weaker-export or recycling-dominated regimes. Such a shift would weaken atmospheric moisture supply lines that currently buffer agricultural zones against drought^48,50^.

Vegetation-induced changes in downwind moisture availability can be interpreted within a conceptual framework in which enhanced ET is partitioned between local moisture recycling and atmospheric export. The balance between these pathways is further modulated by surface energy partitioning, including changes in albedo, aerodynamic roughness, and boundary-layer processes^49,53^. Crucially, this framework operates bi-directionally: while vegetation greening alters transport fractions, land-use change-induced LAI reduction reverses these pathways with distinct non-linear thresholds (as prominently illustrated in Fig. 3). When LAI increases from very low levels, the associated increase in ET may largely remain within the local land–atmosphere system through moisture recycling. Under such conditions, a substantial fraction of the additional moisture may return as local precipitation rather than being transported downwind, which can lead to weak or even negative responses in downwind soil moisture^40,54^. As vegetation greening continues to a certain level, ET can increasingly exceed the effective capacity of local recycling, allowing a larger fraction of evapotranspired moisture to be exported through atmospheric transport. The resulting positive source influence, therefore, likely reflects increased atmospheric moisture export associated with enhanced ET.

Within this conceptual framework, the non-local land–atmosphere coupling and its non-linear behavioral shifts vary systematically across the identified hydroclimatic gradients. In the humid tropical core in the R1 zone (Fig. 3a), the system exhibits a non-linear response that is highly sensitive to canopy degradation (Fig. 3b). While positive LAI anomalies show an increasing effect because background moisture is perennially abundant and the system is limited by radiation, negative LAI anomalies follow a different trajectory. Specifically, lowering LAI, whether driven by anthropogenic deforestation or climate-driven dieback, directly reduces moisture export along the dominant atmospheric transport pathways. This non-linear sensitivity underscores that canopy loss in the humid core can substantially alter soil moisture downwind, directly reducing the remote moisture supply during critical periods.

Moving away from the humid core region, the response pattern observed in the R2 regime (Fig. 3c) is consistent with environments of intermediate moisture availability, such as the Amazon-Cerrado transition zone. In these regions, the transition from recycling-dominated to export-dominated behavior appears to occur at relatively small positive LAI anomalies. Once this transition is reached, further increases in LAI are associated with a stabilization of positive source influence, which may reflect increasing constraints from local recycling and surface energy partitioning. Conversely, within the context of intense land-use change, even minor LAI decreases in R2 (Fig. 3c), characteristic of the Amazon-Cerrado transition zone, can abruptly shift the system back into a recycling-dominated state, effectively shutting down downwind moisture relief during critical periods. The R3 regime (Fig. 3d), characteristic of drier and more seasonally dynamic climates, suggests a different balance between these processes. Initial greening may enhance ET under relatively dry atmospheric conditions, favoring the export of atmospheric moisture and increasing source influence. However, continued increases in vegetation density may reduce the amount of ET available for atmospheric transport, potentially weakening or even reversing the influence of upwind land conditions on downwind soil moisture, through associated changes in near-surface humidity, boundary-layer structure, precipitation efficiency, surface roughness, and even local soil moisture deficit^57^. In the driest highland environments (R4, Fig. 3e), source influence is weak and predominantly negative, with local recycling dominating and upwind vegetation exerting little net downwind effect.

The framework is general and could be applied to other large-scale forests, such as those in central Africa and Southeast Asia^33,58^, to investigate whether comparable teleconnections support hydrological resilience elsewhere. While the present framework focuses on a transport-informed and observation-driven representation of vegetation–soil moisture teleconnections, future work could explore complementary data-driven or network-based approaches to further characterize large-scale moisture connectivity, e.g., network-based moisture analysis^33^ and graph-based learning^59^ methods.

Our results have important implications for Earth system models (ESMs), particularly for representing the role of upwind terrestrial processes in shaping soil moisture variability downwind. The substantial performance gain achieved by incorporating transport-informed upwind information (Fig. 1) indicates that vegetation and other land surface factors modulate soil moisture not only locally but also through atmospheric moisture transport, and reflects a pathway that is often simplified or inadequately represented in ESMs^58,60^. In this context, the proposed framework (Fig. S3) offers an observation-driven reference for evaluating whether models appropriately capture the sensitivity of soil moisture variability to upwind land surface conditions under contemporary climate conditions. Moreover, the identified directional asymmetries (Fig. 2) and climate-dependent response regimes (Fig. 3) provide physically interpretable patterns that may be used to diagnose whether large-scale models reproduce observed non-local land–atmosphere coupling. However, our analysis focuses on the contemporary period (2001–2018) and its direct applicability to future climate projections is therefore limited. Future work could compare contemporary ESM simulations of soil moisture over South America with the observationally informed patterns identified here. Such comparisons may help assess model realism and, if combined with independent constraints and longer records, could contribute to emergent-constraint–type applications^61,62^ across the Coupled Model Intercomparison Project ensemble.

While our observation-driven framework provides a robust approach for quantifying vegetation’s remote influence on soil moisture, several limitations should be considered. First, soil moisture products may affect the estimated teleconnection strength. The SoMo.ml dataset^63^ upscales sparse in-situ data and can be uncertain in data-sparse regions such as the Amazon Basin, while ESA CCI^64^ has substantial missing data over dense rainforest (Fig. S22) and inherits retrieval and assimilation uncertainties^65^. We gap-filled ESA CCI with ERA5-Land data (Methods). Despite missing data and remaining spatial biases in ESA CCI (Fig. S22), comparable sensitivity estimates from SoMo.ml and ESA CCI support the robustness of the dominant source-sink patterns (Fig. S23). Second, the coarse spatial resolution (1.5°) cannot resolve fine-scale heterogeneity. Processes such as vegetation interactions, rooting-depth variability, and soil-microbial feedbacks likely shape local coupling^66^ and may modulate some regional signals even if large-scale patterns persist. Third, we use climatological transport fields from 2008 to 2017 to inform the model, so transient circulation anomalies and short-term feedbacks are not explicitly represented. Finally, the framework identifies robust statistical associations that are physically consistent with known mechanisms but does not by itself establish causality. Targeted perturbation experiments with dynamic vegetation-climate models and observational tests during documented greening or deforestation episodes provide a path for evaluating causal pathways. While our analysis focuses on surface soil moisture (0-10 cm) to capture rapid land-atmosphere coupling, deeper soil layers play a critical role in sustaining vegetation water uptake and regulating LAI variability through root-zone water availability^67^. Root-zone soil moisture typically exhibits longer memory and integrates hydrological processes over seasonal timescales^27,28^. The transport-informed model partly accounts for persistence effects through the temporal structure of SMA anomalies (1-6 months), but does not explicitly represent root-zone dynamics^68^. Furthermore, cross-product comparisons (Fig. S24) reveal significant subsurface divergence, likely due to high land surface heterogeneity and a lack of direct satellite constraints at depth. Incorporating deeper soil moisture may therefore modify the magnitude, persistence, or timing of vegetation-mediated influences inferred here. How such differences would affect the spatial expression and regime structure of vegetation–soil moisture teleconnections remains an open question and represents an important direction for future work, particularly as observation-constrained root-zone soil moisture products with sufficient spatial coverage and uncertainty characterization become available.

Looking ahead, intensifying land-use change and climate variability will likely increase the relevance of vegetation-driven moisture connectivity. Current monitoring and modeling systems largely treat land–atmosphere coupling as local^69^. Incorporating directional source-sink interactions could improve drought early warning and risk assessment and may require revising concepts spanning ecosystem resilience, agricultural dependence, and trans-boundary water security^52^. Ultimately, developing tools and policies that account for hydrological interdependence among distant regions should be a priority, as the stability of continental water cycles depends critically on the state and integrity of upwind ecosystems.

## Methods

### Data processing

We use the datasets listed in Table S1 for 2001–2018. All dynamic variables, including soil moisture, LAI, air temperature at 2 m (TA, K), surface downward shortwave radiation (SSRD, W m^−2^), sea surface temperature (SST, K) and ET, are aggregated to monthly means on a 1.5° grid by area-weighted averaging, masked to South American land cells using the ERA5-Land land-sea mask.

Specifically, soil moisture is from the SoMo.ml v1 product (0-10 cm; m^3^ m^−3^)^63^, trained on long-term in-situ measurements to extrapolate daily dynamics. The soil moisture data are converted to anomalies by removing the linear trend and subtracting the 2001-2018 monthly climatology. We assess consistency by correlating the SMA time series with five alternative products (Table S1). Pearson correlation is computed on space-time samples, and significance is evaluated at *p* < 0.01 (Fig. S25). Surface soil moisture is selected because it couples with ET and boundary layer processes, which capture atmospheric moisture transport signals^4,27^.

LAI is obtained from the GIMMS LAI4g v1 product (1982–2020)^70^. Half-monthly values are averaged to monthly, regridded from 1/12° to 1.5° by averaging, and converted to anomalies via detrending and deseasonalizing within the land-sea mask. TA, ET, and SSRD come from ERA5 reanalysis^71^. Daily values are averaged to monthly, and the 0.25° fields are aggregated to 1.5° by averaging, and TA and SSRD anomalies are formed with the land-sea mask. SST represents oceanic influence on SMA in this study. We aggregate global ERA5 SST to monthly means, regrid to 1.5°, and form anomalies. We compute empirical orthogonal functions (EOFs) of global SST anomalies, retain the leading modes^16^, and relate them to SMA using canonical correlation analysis (70% of land grid cells for training and 30% for validation). The first canonical variate is kept as the SST predictor for each land grid cell. We retain the first 40 SST EOF modes, which account for 78% of SST variance. To represent atmospheric moisture transport, we use the global UTrack dataset^34^, a Lagrangian climatology of evaporation recycling at 0.5° resolution derived from ERA5 for 2008–2017. Moisture source fractions are regridded to 1.5° by averaging and aligned with the land-sea mask. Soil properties from SoilGrids^72^ at 250 m resolution are used as static inputs, including clay content (CLAY, %), bulk density (BDOD, g cm^-3^), silt content (SILT, %), and sand content (SAND, %). We use the surface layer (0–5 cm), regridded to 1.5° by averaging.

We include tree cover loss and cropland cover as auxiliary data for post-model analysis. Tree cover loss is from the Global Forest Watch^73^ (3 km native resolution). We generate binary maps of loss greater than 30% per grid cell and convert to fractional loss on the 1.5° grid for 2001–2003, 2004–2008, 2009–2013, and 2014–2018. Cropland (defined where the cropland fraction exceeds 10%) is from ref. ^74^ (3 km, 2003–2019, four-year intervals). We aggregate them to 1.5°, mask with the land-sea mask, and assign them to the study period as follows: 2001–2003 uses 2003 cropland, 2004–2008 uses 2008 cropland, 2009–2013 uses 2013 cropland, and 2014–2018 uses 2018 cropland.

### ESA CCI gap-filling

We validate SoMo.ml with ESA CCI surface soil moisture. To address missing data in the Amazon (Fig. S22), we gap-fill ESA CCI data using ERA5-Land^75^. ERA5-Land hourly data are aggregated to daily, temporally aligned with ESA CCI, and rescaled to the satellite range. For each 0.25° grid with concurrent observations, we rescale ERA5-Land to ESA CCI by mean–variance matching (a linear transform that maps the ERA5-Land mean and standard deviation onto those of ESA CCI), and use it to fill missing ESA CCI values:

1 $${{\rm{SM}}}_{{\rm{filled}}}=\frac{{{\rm{\sigma }}}_{{\rm{ESA\; CCI}}}}{{{\rm{\sigma }}}_{{\rm{ERA}}5-{\rm{Land}}}}\times {{\rm{SM}}}_{{\rm{ERA}}5-{\rm{Land}}}+{{\rm{\mu }}}_{{\rm{ESA\; CCI}}}-\frac{{{\rm{\sigma }}}_{{\rm{ESA\; CCI}}}}{{{\rm{\sigma }}}_{{\rm{ERA}}5-{\rm{Land}}}}\times {{\rm{\mu }}}_{{\rm{ERA}}5-{\rm{Land}}}$$

where $\mu$ and $\sigma$ are the per-cell time-series mean and standard deviation over the overlapping period. For fully missing grid cells, we apply a $3\times 3$ moving-window average and refit iteratively until stable. We then compute anomalies under the land-sea mask and evaluate the models with the same configuration used for SoMo.ml.

### Model structures

We develop three predictive deep learning models to estimate SMA, each incrementally adding spatial and physical context to isolate the effect of atmospheric transport information. All models share the same input setup, predicting monthly SMA at each land grid cell from four dynamic anomalies (SST, LAI, TA, SSRD) and four static soil properties. Each model uses a convolutional neural network (CNN) that ingests the previous 5 months, along with current conditions, to account for soil moisture memory. Architectural details and all hyperparameters are provided in the Supplementary Information.

1. The local-only model serves as the baseline model, relating only local predictors at the target grid cell to local SMA via one-dimensional convolutional layers and fully connected layers.
2. The spatial-context model expands this view by incorporating non-directional surrounding context from a 21 × 21 grid-cell window (31.5° × 31.5°) centered on each target cell. The window size is chosen using UTrack coverage to include over 90% of terrestrial moisture source regions (Fig. S26). Three-dimensional convolutional layers extract joint spatiotemporal features from all cells in the window, without imposing wind directionality.
3. The transport-informed model further introduces a physically guided attention mechanism to capture the directionality of atmospheric moisture transport (Fig. S3). It retains the core architecture of the spatial-context model and adds an attention module (see Supplementary Information) trained to weight surrounding grid cells according to climatological moisture source patterns from the UTrack dataset^34^. Specifically, UTrack source percentages enter as a soft constraint through an additional loss term, guiding the learned attention map to align with physical knowledge, so that upwind source regions receive higher weights. The designed loss function is as follows:

2 $${Loss}=\mathop{\sum }\limits_{i=1}^{n}\left[{({y}_{{\rm{SMA}},{\rm{obs}}}^{i}-{y}_{{\rm{SMA}},{\rm{pred}}}^{i})}^{2}+{({\omega }_{{\rm{Atmos}},{\rm{UTrack}}}^{i}-{\omega }_{{\rm{Atmos}},{\rm{pred}}}^{i})}^{2}\right]$$

where ${y}_{{\rm{SMA}},{\rm{obs}}}$ and ${y}_{{\rm{SMA}},{\rm{pred}}}$ denote the observed and predicted SMA values for the ${i}_{{th}}$ output sample, respectively; $n$ is the total prediction number in the transport-informed model; and$\,{\omega }_{{\rm{Atmos}},{\rm{Utrack}}}$ and ${\omega }_{{\rm{Atmos}},{\rm{pred}}}$ denote the UTrack source percentages and predicted source percentages, respectively. Simply adding UTrack as an input feature performs worse (Fig. S27), because directional constraints cannot be learned from raw features alone.

### Model training and running

All inputs and outputs use min-max scaling based on global extrema. The dataset contains 733 land grid cells at 1.5° resolution. Models are evaluated with spatial four-fold cross-validation under four random seeds, with three folds for training and one for testing per split, ensuring that neighboring cells are not simultaneously in train and test sets. Hyperparameters, including batch size 4, learning rate 0.001, and a maximum of 500 epochs, are selected by grid search. Computations run on a system with 8 GPUs and 500 GB of memory.

### Model performance evaluation

Model performance is evaluated using the coefficient of determination (R^2^) computed on held-out folds. Reported values are averaged across the four folds. To capture different dimensions of model skill, we compute: (1) An overall R^2^ by concatenating test-set predictions across all grid cells and months; (2) A per-grid-cell temporal R^2^ to map spatial patterns. Differences between models are expressed as ΔR^2^ relative to the local-only and spatial-context baselines (Fig. 1); (3) To examine seasonal structure, we average per-grid-cell R^2^ over space for each calendar month, producing the monthly skill shown in Fig. S4. For context, Fig. S5 shows model performance across grid cells as a function of the 2001–2018 mean and standard deviation of LAI anomalies.

### Predictive attribution

To determine how much each terrestrial input contributes to the model’s predictions, we estimate predictive attribution in the transport-informed model using expected gradients^38^ and verify patterns with GradientSHAP^76^. The expected gradients method integrates gradients from a reference distribution to produce attribution scores for every variable-location pair (see Supplementary Information). Attributions are rescaled to physical units (m^3^ m^−3^) by inverting the min-max normalization of the output. To identify long-term drivers in prediction, we summarize contributions by averaging absolute attributions over time. Shares are computed as the fraction of total absolute attribution assigned to a given variable or region (Fig. 1c and d). The anomaly-based formulation allows attribution signs to be interpreted directionally (Fig. S11). To summarize the spatial structure of variable-wise attribution, we average the EG values of each variable’s spatiotemporal inputs across grid cells and time steps; the resulting maps are shown in Fig. S12.

### Quantifying upwind dominance

To assess the dominance of upwind processes, we calculate at each target cell and month the ratio between the absolute attribution summed over upwind land grid cells and the absolute attribution summed over all grid cells in the model’s 21 × 21 input window. Upwind cells are those whose UTrack fractional moisture contribution to the target cell exceeds 0.1%. The denominator includes the local cell, and ocean cells are excluded throughout. We then average this ratio over time and across land cells to produce the map in Fig. 1c. Variable-specific contributions (for example, LAI) are calculated the same way, except that the numerator and denominator are restricted to the variable of interest; all results use absolute attributions (Fig. 1d).

### Upwind region, source influence, sink sensitivity, and asymmetry index

Upwind regions are identified using UTrack^34^, which quantifies non-local moisture contributions between grid cells. The original UTrack dataset provides pixel-level moisture source contributions for each target grid cell. In this study, upwind regions are defined as grid cells within the 21 × 21 window around each target cell whose fractional moisture contribution to that target grid cell exceeds 0.1%, using a land mask from ERA5.

We define two metrics based on the LAI anomalies’ predictive attributions (expected gradients) from the transport-informed model. For each month, the attribution from a cell’s LAI anomalies to a target cell’s SMA is signed, so that its sign indicates the direction of the ecological effect (positive: greening associated with wetter downwind soil; negative: with drier downwind soil). Source influence quantifies the outgoing effect of a given cell. For any source cell $i$, it is computed as the signed attribution from its LAI anomaly to soil moisture at each downwind target cell $j$, averaged across all downwind target cells (${N}_{{\rm{downwind}}}$) within its 21 × 21 neighborhood:

3 $${\rm{Source\; Influence}}(i)=\frac{1}{{N}_{{\rm{downwind}}}}\mathop{\sum}\limits_{j\in {\rm{downwind}}}{\rm{Attribution}}({{\rm{LAI\; anomaly}}}_{i}\to {{\rm{SMA}}}_{j})$$

Sink sensitivity quantifies the incoming sensitivity of a given cell. For a given target grid cell $j$, it is the signed attribution from upwind cells’ LAI anomalies to that target’s SMA, averaged over all upwind source cells (${N}_{{\rm{upwind}}}$) in the same neighborhood.

4 $${\rm{Sink\; sensitivity}}(j)=\frac{1}{{N}_{{\rm{upwind}}}}\sum\limits_{i\in {\rm{upwind}}}{\rm{Attribution}}({{\rm{LAI\; anomaly}}}_{i}\to {{\rm{SMA}}}_{j})$$

Retaining the sign is what allows the directional interpretation used in Fig. 2 and the sign-persistence classification of regimes in Fig. 3. For the maps in Fig. 2a and b, both the source influence and sink sensitivity are averaged over time to give a single value per cell; for the regime classification in Fig. 3, we instead use the monthly source influence and quantify how often it is positive (see Definition of climatic regimes).

Source influence and sink sensitivity quantify the asymmetric roles of grid cells in the directional transmission of predictive information related to non-local vegetation effects on SMA. To summarize this directional imbalance, we compute an asymmetry index from the magnitudes of the two time-mean metrics, so that it compares the strength of the influence a cell exerts downwind (outgoing) with the influence it receives from upwind (incoming):

5 $${\rm{Asymmetry\; Index}}=\,\frac{\left|{\rm{Source\; Influence}}\right|-\left|{\rm{Sink\; Sensitivity}}\right|}{\left|{\rm{Source\; Influence}}\right|+\left|{\rm{Sink\; Sensitivity}}\right|+{\rm{\epsilon }}}$$

with ${\rm{\epsilon }}={10}^{-5}$ for numerical stability. The index ranges from −1 to +1, where values approaching +1 indicate a predominantly outgoing (source-dominated) cell, values near 0 indicate balance, and values near -1 indicate a predominantly incoming (sink-dominated) cell. The index does not diagnose whether a location exports or imports moisture on average; rather, it indicates whether vegetation at a grid cell exerts a stronger influence on downwind soil moisture than it receives from upwind vegetation. When outgoing contributions dominate, the cell acts as a primary transmitter of predictive information to downwind regions; conversely, when incoming contributions dominate, it is primarily influenced by upwind vegetation (as shown in Fig. S14).

### Application to croplands and drought events

Cropland sink sensitivity is computed using the cropland land-cover dataset. For each cropland cell and month, we compare the LAI-derived sink sensitivity with the median across the eight input variables at that cell and month, and classify the cell as LAI-dominant if the LAI-derived sink sensitivity exceeds this median. For each month, we then compute the sensitivity ratio, i.e., the fraction of cropland cells classified as LAI-dominant, and report its time series (Fig. S16); averaged over 2001–2018, this ratio is 55%. We also report the area-weighted mean sink sensitivity over croplands.

We identify droughts as connected spatiotemporal events where soil moisture falls below the 10th percentile threshold derived from the 1978–2018 ERA5-Land climatology, using mathematical morphology for characterization^77,78^. Corresponding upwind grid cells are located with UTrack. Event-level sink sensitivity is obtained by averaging over each drought event and its associated upwind source set (Fig. S17).

### Definition of climatic regimes

We classify each land grid cell into one of four regimes that summarize how upwind vegetation couples to downwind soil moisture. The classification proceeds in two stages: we first stratify cells by the temporal sign persistence of the source influence, and then, within the persistently positive cells, by the shape of the non-linear response to LAI anomaly (an example of non-linear EG responses to LAI anomalies is provided in Fig. S13). Here, the source influence is evaluated monthly. Before classification, we exclude hyper-arid cells and cells whose source influence is not statistically distinguishable from zero, so that the regimes describe robust and active land-atmosphere interactions. The remaining cells are then classified as follows.

In stage 1, we quantify sign persistence as the percentage of months in which the monthly source influence is positive, and test it against a null hypothesis of random sign occurrence using a Binomial test (the probability of a positive month *p* = 0.5, *α* = 0.05). This splits cells into three bands: persistently positive ( > 70% of months), transitional (30–70%), and persistently negative ( < 30%). A high persistence ( > 70%) identifies a stable, climatologically consistent positive covariation between upwind LAI and downwind moisture.

In stage 2, for persistently positive cells, we further characterize the coupling by fitting a single-breakpoint piecewise linear model to monthly source influence ($y$) versus LAI anomaly ($x$) at each cell (Eq. 6). The breakpoint is a freely floating variable optimized for each grid cell by minimizing the residual sum of squares using a genetic algorithm. Hence, the model independently determines the vegetation threshold at which the coupling intensity shifts for each location. Both pre- and post-breakpoint slopes (${k}_{1},{k}_{2}$) are required to be significant (two-tailed t-tests, *p* < 0.05); the sign of (${k}_{2}-{k}_{1}$) then separates a strengthening from a saturating coupling.

6 $$y=\left\{\begin{array}{c}{k}_{1}x+{b}_{1},x < {\rm{breakpoint}}\\ \,{k}_{2}x+{b}_{2},x\ge {\rm{breakpoint}}\end{array}\right.$$

where ${k}_{1}$ and ${k}_{2}$ are the slopes before and after the breakpoint, respectively, and ${b}_{1}$ and ${b}_{2}$ are intercepts.

The four regimes (Fig. 3b–e) follow directly from these two criteria. Regime 1 (positive-increasing; Fig. 3b) is identified by more than 70% positive months with a steepening slope (${k}_{1} < {k}_{2}$), so that the coupling strengthens as the LAI anomaly increases. Regime 2 (positive-saturating; Fig. 3c) is identified by more than 70% positive months with a flattening slope after the breakpoint (${k}_{1} > {k}_{2}$), so that the positive influence plateaus beyond a certain LAI anomaly. Regime 3 (transition; Fig. 3d) is identified by 30–70% positive months, for which the vegetation-soil moisture link is inconsistent and frequently switches sign. Regime 4 (negative; Fig. 3e) is identified by fewer than 30% positive months, for which the LAI anomaly and downwind moisture supply are predominantly inversely associated. Every non-masked cell falls into one of these four regimes, and Fig. S21 confirms that the classification is robust to alternative persistence thresholds around the 70% criterion.

## Supplementary information

Supplementary Information

Transparent Peer Review file

## Data Availability

GIMMS LAI4g^70^ data are available at 10.5281/zenodo.7649107. The SoMo.ml^63^ soil moisture data are available at 10.17871/BGI_SOMO.ML_V1_2020. ESA CCI^64^ soil moisture data are available at https://climate.esa.int/en/projects/soil-moisture. ERA5-Land^71^ climate and soil moisture reanalysis datasets are available at 10.24381/cds.e2161bac. ERA5 reanalysis^71^ data are available at 10.24381/cds.adbb2d47. SoilGrids^72^ soil properties are available at https://soilgrids.org. UTrack^34^ data are available at 10.1594/PANGAEA.912710. Cropland^74^ data are available at https://glad.umd.edu/dataset/croplands. Global Forest Watch^73^ data are available at https://data.globalforestwatch.org. The data used to generate the figures in this study are available at Zenodo^79^ and are cited in the reference list.

## Code availability

The codes required for reproducing the results and figures in the main text have been deposited at 10.5281/zenodo.19063712.
