# Supplementary material for: Upwind terrestrial influences on soil moisture variability across South America
Source: Nat Commun. 2026 Jul 24;17:7256. doi: 10.1038/s41467-026-75637-x (PMC13400732; doi:10.1038/s41467-026-75637-x)
Supplement: Supplementary file 1 — Supplementary Information [file 41467_2026_75637_MOESM1_ESM.pdf]

Supplementary Information for "Upwind terrestrial influences on soil moisture variability across South America"

Feini Huang[1,2,3,4,5], Shijie Jiang[2,3,*], Wei Shangguan[1,5,*], Gustau Camps-Valls[6], Alexander Winkler[2,3], Wantong Li[7,8], Gregory Duveiller[2], Christian Reimers[2,3], Wenli Zhao[9], Markus Reichstein[2,3], Yongjiu Dai[1]

[1] School of Atmospheric Sciences, Sun Yat–Sen University, Zhuhai, China.

[2] Department of Biogeochemical Integration, Max Planck Institute for Biogeochemistry, Jena, Germany.

[3] ELLIS Unit Jena, Jena, Germany

[4] Guangdong Province Key Laboratory for Climate Change and Natural Disaster Studies, Sun Yat–Sen University, Zhuhai, China.

[5] Southern Marine Science and Engineering Guangdong Laboratory (Zhuhai), Zhuhai, China.

[6] Image Processing Lab (IPL), Universitat de València, Valencia, Spain

[7] Department of Environmental Science, Policy and Management, UC Berkeley, Berkeley, CA, USA

[8] Climate and Ecosystem Sciences Division, Lawrence Berkeley National Laboratory, Berkeley, CA, USA

[9] Department of Earth and Environmental Engineering, Columbia University, New York, NY, USA

**Corresponding authors*

Shijie Jiang, sjiang@bgc-jena.mpg.de

Wei Shangguan, shgwei@mail.sysu.edu.cn

Contents

1. Model component basis 1
1.1 Convolutional layer 1
1.2 Spatial attention mechanism 1
1.3 Fully connected neural network and flatten layer 2
1.4 Dropout, max-pool and batch normalization layers 2
1.5 Model settings and hyperparameters 2
2. Model interpretation 3
2.1 Expected gradients 3
2.2 GradientSHAP 4
2.3 Integrated gradients 4
2.4 Interpretation uncertainty assessment 4
Table S1 | Data sources and specifications of input variables used in the study. 6
Fig. S1 | Concept of convolutional layer components. 7
Fig. S2 | Structure of spatial attention mechanism. 8
Fig. S3 | Transport-informed deep learning model architecture for predicting soil moisture anomaly (SMA) at a target land grid cell using terrestrial inputs. 9
Fig. S4 | Monthly averaged temporal R^2 values for local-only, spatial-context and transport-informed models. 10
Fig. S6 | Performance gains of the transport-informed model and predictive attribution from land surface and near-surface atmospheric inputs based on ESA CCI soil moisture dataset. 12
Fig. S7 | Spatial structure of average monthly precipitation across South America. 13
Fig. S8 | Variable-wise spatial distribution of predictive attribution within upwind grid cells in the transport-informed model. 14
Fig. S9 | Structure similarity index metrics (SSIM) of integrated gradients (IG), GradientSHAP (GS), and expected gradients (EG) explanation. 15
Fig. S10 | Standard deviation of the predictive attribution for eight input variables from four different trained models in four random seeds. The geographic base map in a-h, including coastlines and country borders, is generated using the public-domain Natural Earth dataset via Python's Cartopy library. 16
Fig. S11 | Directional dependence of predictive attribution (Expected Gradients) on input variables in transport-informed model. 17
Fig. S12 | Spatial distribution of averaged predictive attribution (Expected Gradients, EG) for individual input variables. 18
Fig. S13 | An example of nonlinear responses of predictive attribution (Expected Gradients, EG) to LAI anomalies. 19
Fig. S14 | Schematic illustration of source influence, sink sensitivity, and the asymmetry index. 20
Fig. S15 | Tree loss area across South America for four historical periods. 21
Fig. S16 | Sink sensitivity in cropland across South America. 22
Fig. S17 | Event-based sink sensitivity of soil moisture droughts to upwind vegetation. 23
Fig. S18 | Comparison of sink sensitivity and source influence between growing season (DJF) and non-growing season (JJA) across South America. 24
Fig. S19 | Cascade of Amazonia vegetation to soil moisture anomaly in the Brazilian agricultural zone. ... 25
Fig. S20 | Meteorological anomalies and LAI anomalies in the cascade of Amazonia vegetation to soil moisture anomaly in the Brazilian agricultural zone. 26
Fig. S21 | Sensitivity of regime classification to persistence thresholds. 27
Fig. S22 | ESA CCI data completeness and validation metrics. 28
Fig. S23 | Comparable analysis of sensitivity derived from SoMo.ml and ESA CCI. 29
Fig. S24 | Pearson correlation of different sources of subsurface soil moisture anomaly data. 30
Fig. S25 | Pearson correlation of different sources of surface soil moisture anomaly data. 31
Fig. S26 | The proportion of terrestrial water vapor sources outside the defined window. 32
Fig. S27 | Model performance (R^2 values) of deep learning model with the UTrack data as one of the input variables based on two different soil moisture datasets. 33
Supplementary References 34

1. Model component basis

In this section, the components of the deep learning model employed in our experiment are introduced. These components include convolutional layers in convolutional neural networks (CNN), a spatial attention mechanism, and fully connected linear layers.

1.1 Convolutional layer

Convolutions are a form of filtering that involves applying a learnable matrix or vector of parameters to extract low-dimensional features from an input dataset. Convolutional neural networks leverage the concept of spatial locality by enforcing a local connectivity pattern between neurons across adjacent layers[1]. Intuitively, convolution can be understood as the process of applying a sliding window (a filter with learnable weights) to the input data, resulting in a weighted sum (of weights and input) as the output. This weighted sum constitutes the feature space used as input to subsequent layers.

(1) Conv1D

The majority of simplistic convolutions are of the Conv1D (1D convolutional layer) variety. These are most commonly used on sequence datasets, although they can also be applied to other applications. They can be used to extract local one-dimensional subsequences from local patterns within the convolutional window. **Fig. S1a** shows how a one-dimensional convolution filter is applied to a sequence to obtain new features.

(2) Conv3D

Conv3D (3D convolutional layer) is a sophisticated deep learning module that applies a 3-dimensional filter to the dataset, thereby enabling the calculation of low-level feature representations. This is achieved by applying sliding cuboidal convolution filters to 3-dimensional input, with the filters moving vertically, horizontally, and along the depth to convolve the input (**Fig. S1b)**. The incorporation of an additional dimension, time, into the convolutional process enables Conv3D to comprehend both spatial and temporal characteristics.

1.2 Spatial attention mechanism

A spatial attention mechanism module generates a spatial attention map in CNN by leveraging the inter-spatial relationships among features, which contrasts with channel attention[2], which focuses on where which feature channels contain informative features (**Fig. S2**). Spatial attention complements channel attention. To compute spatial attention, the following steps are taken: first, average- and max-pooling operations are applied along the channel axis, and then the outputs are concatenated to generate an efficient feature descriptor. The concatenated feature descriptor is then processed by a convolution layer, which generates a spatial attention map that encodes the regions to emphasize or suppress.

1.3 Fully connected neural network and flatten layer

(1) Fully-connected neural network (FNN)

In the context of neural networks, a fully connected layer is one in which each neuron applies a linear transformation to the input vector using a weight matrix. Consequently, this implies that all possible connections layer-to-layer are present, signifying that every input of the input vector influences every output of the output vector.

(2) Flatten layer

A flatten layer reshapes a multi-dimensional tensor into a one-dimensional vector. It serves as a critical connection between convolutional layers and fully connected layers, enabling high-level feature integration.

1.4 Dropout, max-pool and batch normalization layers

(1) Dropout

Dropout is a regularization technique that reduces overfitting in neural networks. During training, it randomly drops (i.e., temporarily deactivates) a random subset of neurons in a layer for each training example. This prevents neurons from becoming co-dependent and forces the network to learn more robust features. It significantly improves generalization performance by preventing the network from over-relying on any single neuron or set of neurons.

(2) Max-pool

Max-Pool is a down-sampling operation that reduces the spatial dimensions (width and height) of input feature maps. It partitions the input into rectangular regions (e.g., 2x2 pixels) and outputs the maximum value for each region. This effectively summarizes the most salient feature presence within that window. It provides a form of translation invariance, making the network less sensitive to the exact position of features and reduces computational complexity and memory usage for subsequent layers, thereby helping to control overfitting.

(3) Batch normalization (BatchNorm)

BatchNorm accelerates and stabilizes the training of deep neural networks. It normalizes the inputs to a layer by adjusting and scaling activations to have zero mean and unit variance across each mini-batch. This is followed by scaling and shifting with two learnable parameters (gamma and beta). BatchNorm can mitigate internal covariate shift (changes in network activation distributions during training) and enable the use of higher learning rates.

1.5 Model settings and hyperparameters

(1) Local-only model

The local-only model employs three one-dimensional temporal convolutional layers (channel sizes: 128, 512, 1024) to relate local predictors to SMA per grid cell. Each layer uses size-3 kernels to integrate the previous five months and current data, with padding=0, stride=1, dropout (0.1), batch normalization, LeakyReLU activation, and max-pooling. The input tensor (N × 8 × 216, N: number of samples) is processed through these convolutional layers, flattened, and passed through three fully connected layers (sizes: 441, 484, 986) with the same regularization and activation, yielding an output of size 1 × 210.

(2) Spatial-context model

Similar to the local-only model, the spatial-context model extends the architecture to include three-dimensional spatiotemporal information from surrounding regions. A 21×21 window selected based on UTrack moisture tracking data which captures over 90% of terrestrial moisture sources across South America (**Fig. S26**) is centered on each target land cell. The input tensor (N × 8 × 216 × 21 × 21) is processed through three 3D-CNN layers (channel sizes: 128, 512, 1024), each with 3×3×3 kernels, padding (0, 1, 1), stride 1, dropout (0.1), 3D batch normalization, max-pooling, and LeakyReLU. The resulting features are flattened and passed through the same fully connected layers as the local-only model to produce the output.

(3) Transport-informed model

The transport-informed model uses the same architecture and input/output structure as the spatial-context model but incorporates an additional attention module guided by climatological moisture transport fields (**Fig. S3**). This module processes the CNN outputs through a spatial attention mechanism that combines max- and mean-pooling, followed by a 3D convolution (kernel: (1, 3, 3), padding: (0, 1, 1), stride: (1) and sigmoid activation. The attention maps are constrained via a mean squared error loss using UTrack-based moisture source percentages corresponding to the 21×21 monthly window, with equal weight to the primary SMA prediction task. For comparison, a model using UTrack data directly as input performed worse (**Fig. S27**), confirming the advantage of the attention-based constraint.

2. Model interpretation

2.1 Expected gradients

In this study, we use expected gradients[3] (EG) to quantify the contribution of each input feature to the model prediction. The expected gradients method averages integrated-gradient attributions over reference samples drawn from a background distribution and over points sampled along the straight-line path between each reference sample and the input. For an input sample x, the expected gradients attribution of feature i is defined as

$$\mathrm{EG}_i(x) = \mathbb{E}_{x' \sim D,\, \alpha \sim U(0,1)}\left[(x_i - x_i')\frac{\partial f\big(x' + \alpha(x - x')\big)}{\partial x_i}\right] \tag{1}$$

where D is the background distribution from which the reference sample x' is drawn, α is sampled from a uniform distribution between 0 and 1, and f denotes the trained model. The term $x' + \alpha(x - x')$ represents a point along the interpolation path from the reference sample to the input sample. The sign of $\mathrm{EG}_i(x)$ indicates the direction of the feature contribution relative to the reference distribution, while its magnitude represents the strength of that contribution.

2.2 GradientSHAP

We also use GradientSHAP[4] (GS) as an alternative attribution method to assess the consistency of the expected gradients results. GradientSHAP approximates SHAP values by repeatedly sampling a reference input from a background distribution and a point along the straight-line path between the reference input and the input being explained. For an input sample x, the GradientSHAP attribution of feature i can be approximated as

$$\mathrm{GS}_i(x) \approx \frac{1}{M}\sum_{j=1}^{M} \left(x_i - x_i'^{(j)}\right)\frac{\partial f\left(x'^{(j)}+\alpha_j(x-x'^{(j)})\right)}{\partial x_i} \quad (2)$$

where $x'^{(j)}$ is a reference sample drawn from the background distribution, α_j is independently sampled from a uniform distribution $U(0, 1)$, $M$is the number of Monte Carlo samples, and f denotes the trained model. The final attribution is obtained by averaging the gradient contributions over the sampled reference inputs and interpolation points. The sign of $\mathrm{GS}_i(x)$ indicates the direction of the feature contribution relative to the reference distribution, while its magnitude represents the strength of that contribution.

2.3 Integrated gradients

We also use integrated gradients[5] (IG) as an alternative attribution method for comparison with expected gradients and GradientSHAP. The integrated gradients method attributes the difference between the model output for an input x and that for a reference input x' by accumulating gradients along the straight-line path from x' to x. For feature i, the attribution is defined and numerically approximated as

$$\mathrm{IG}_i(x) = (x_i - x_i')\int_0^1 \frac{\partial f\left(x'+\alpha(x-x')\right)}{\partial x_i}\,d\alpha \approx \frac{x_i - x_i'}{m}\sum_{k=1}^{m} \frac{\partial f\left(x'+\frac{k}{m}(x-x')\right)}{\partial x_i} \quad (3)$$

where x' is the reference input, m is the number of interpolation steps, and k indexes the points sampled along the interpolation path. The sign of $\mathrm{IG}_i(x)$ indicates whether the feature contributes positively or negatively to the model output relative to the reference input, and its magnitude represents the strength of that contribution.

2.4 Interpretation uncertainty assessment

We use structure similarity index metrics[6] (SSIM) to evaluate uncertainty across explanation methods, i.e., EG, SG and IG. SSIM measures the similarity between two images. It is a complete reference metric, meaning that the measurement or prediction of image quality is based on an initial, uncompressed, or distortion-free image that serves as a reference point. It is defined as follows:

$$\mathrm{SSIM}(x, y) = \frac{(2\mu_x\mu_y + (k_1 L)^2)(2\sigma_{xy} + (k_2 L)^2)}{(\mu_x^2 + \mu_y^2 + (k_1 L)^2)(\sigma_x^2 + \sigma_y^2 + (k_2 L)^2)} \tag{4}$$

where μ_x is the pixel sample mean of x, μ_y is the pixel sample mean of y, σ is the variance and σ_{xy} is the covariance of x and y, L is the dynamic range of the pixel-values and k_1 =0.01 and k_2=0.03 by default. When *SSIM(x,y)* is equal to 1, x and y are the same.

Table S1 | Data sources and specifications of input variables used in the study.

Variable	Source	Version	Spatial Resolution	Temporal Resolution	Unit	Depth/ Height
**Soil Moisture**	SoMo.ml	v1	0.25°	daily	m^3/m^3	10cm
	ERA5-Land	e1	0.1°	hourly	m^3/m^3	7cm
	GLEAM	v4.2a	0.1°	daily	m^3/m^3	7cm
	CDS	v202312.0.0	0.25°	daily	m^3/m^3	7cm
	ESA CCI	v08.1	0.1°	daily	m^3/m^3	7cm
	GRACE	-	0.25°		%	-
**Air Temperature**	ERA5	e1	0.25°	daily	K	2m
**Sea Surface Temperature**	ERA5	e1	0.25°	daily	K	0.5-1m
**Evapotranspiration**	ERA5-Land	e1	0.1°	hourly	mm	-
**Surface Solar Radiation Downward**	ERA5	e1	0.25°	daily	W/m^2	-
**Leaf Area Index**	GIMMS	LAI4g	1/12°	half-monthly	m^2/m^2	-
**Bulk Density**	SoilGrids	v2.0	250 m	static	g/cm^3	0-5cm
**Clay Content**	SoilGrids	v2.0	250 m	static	%	0-5cm
**Silt Content**	SoilGrids	v2.0	250 m	static	%	0-5cm
**Sand Content**	SoilGrids	v2.0	250 m	static	%	0-5cm
**Moisture Flows**	UTrack	-	0.5°	monthly	-	-

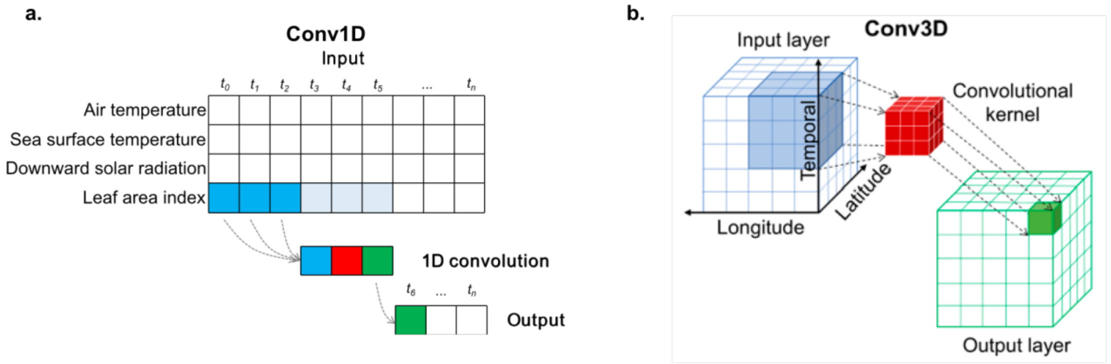

Fig. S1 | Concept of convolutional layer components.

a, one-dimensional convolution (Conv1D); **b**, three-dimensional convolution (Conv3D).

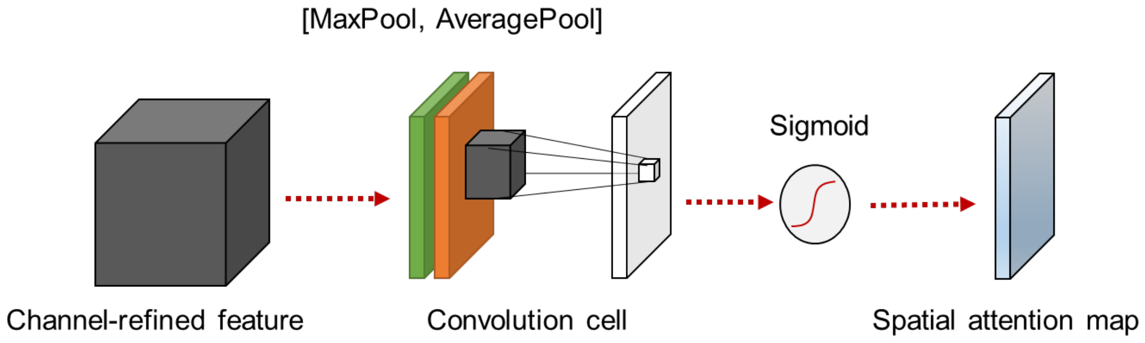

Fig. S2 | Structure of spatial attention mechanism.

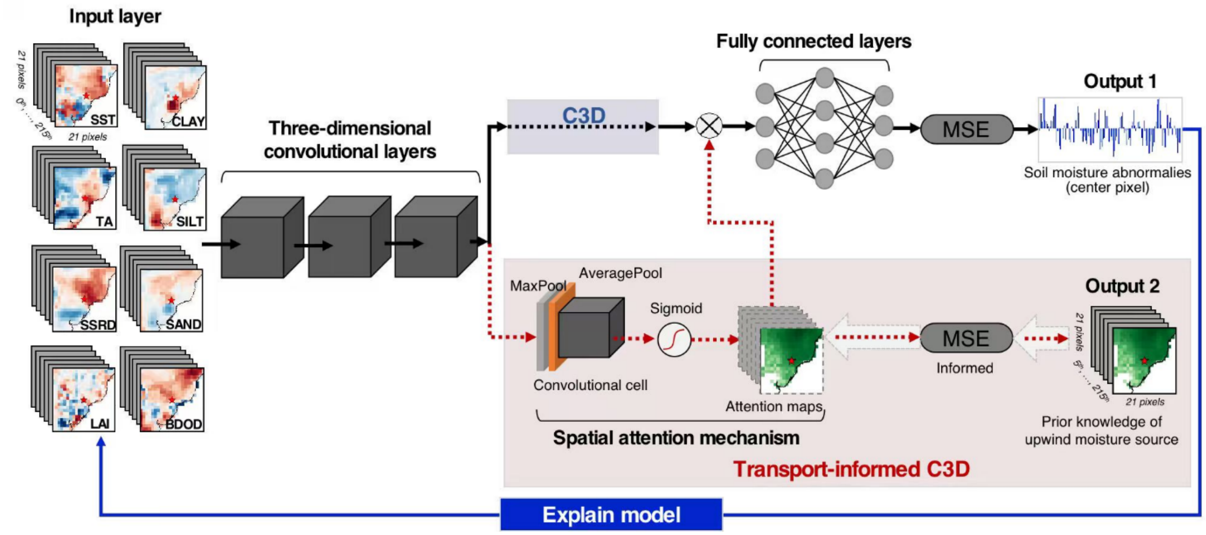

Fig. S3 | Transport-informed deep learning model architecture for predicting soil moisture anomaly (SMA) at a target land grid cell using terrestrial inputs.

The architecture of transport-informed deep learning model is used to link leaf area index (LAI) anomaly and soil moisture anomaly (SMA). The model uses 216 months of input data across eight variables. Each variable is provided over a 21×21-pixel window centered on the target pixel (red star), resulting in an input shape of (8, 216, 21, 21). The transport-informed three-dimensional convolutional network (C3D) incorporates a spatial attention mechanism to identify input regions within the input window most relevant for prediction (highlighted in the red box). In the attention maps, green shading indicates regions assigned higher importance by the model during prediction, reflecting areas with stronger inferred moisture sources.

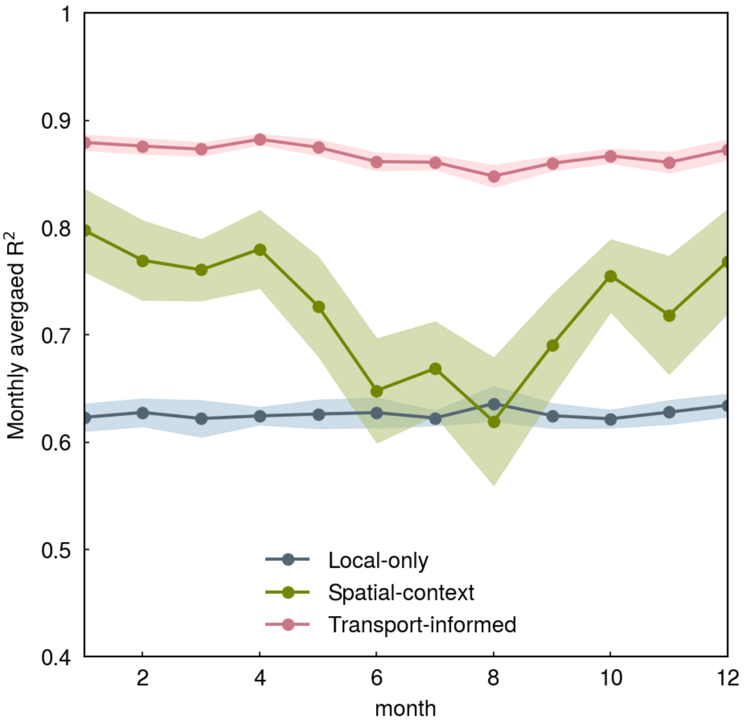

Fig. S4 | Monthly averaged temporal R^2 values for local-only, spatial-context and transport-informed models.

Monthly R^2 values represent temporal averages of spatially computed R^2 at each observation time. The shaded areas, colored to match, represent the 95% confidence intervals (CIs). We use critical value × standard error to estimate the CI. The critical value is that for a 95% CI, this is the t-value that cuts off the top 2.5% of the t-distribution with $n-1$ degrees of freedom. For large samples ($n > 30$), this is approximately 1.96 (from the standard normal distribution, or z-distribution). Standard error is defined as $\frac{s}{\sqrt{n}}$, where s is the sample standard deviation and n is the sample size.

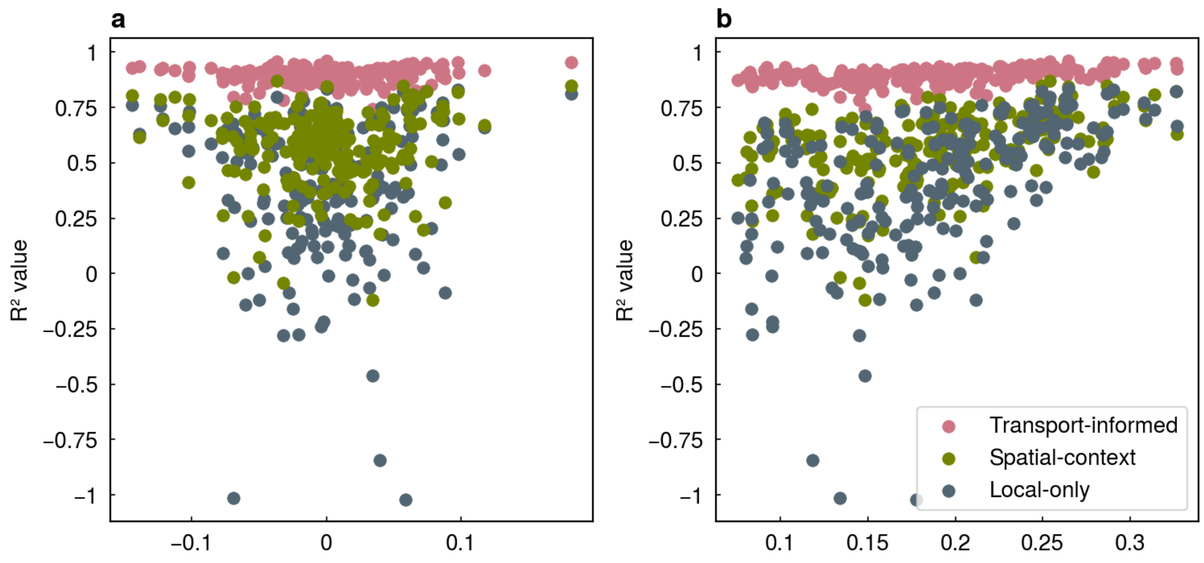

Fig. S5 | Model performance as a function of the temporal mean of LAI anomalies (a) and their temporal standard deviation (b) across grid cells.

a, b, Each marker represents a grid cell. The x-axis in **a** denotes the 216-month temporal mean of LAI anomalies at each grid cell from January 2001 to December 2018, while the x-axis in **b** denotes the corresponding temporal standard deviation. The y-axes in both panels indicate the predicted R^2 values of the grid cells.

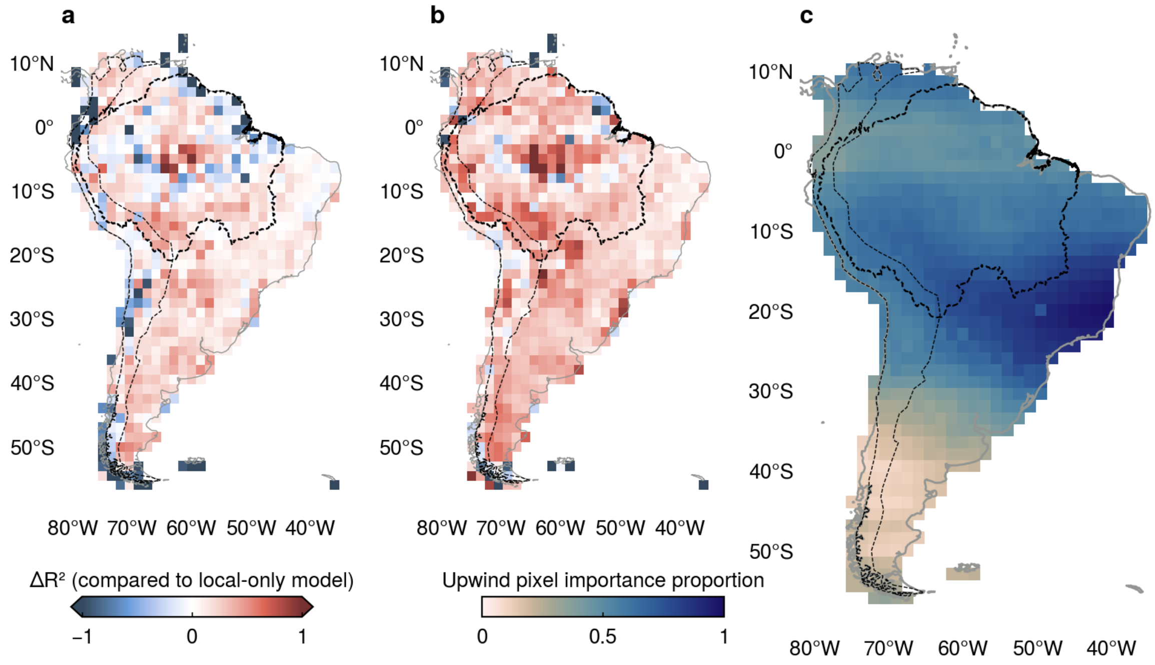

Fig. S6 | Performance gains of the transport-informed model and predictive attribution from land surface and near-surface atmospheric inputs based on ESA CCI soil moisture dataset.

a, ΔR^2 between the spatial-context and local-only models; positive values (red) indicate that adding non-directional spatial context improves skill. **b**, ΔR^2 between the transport-informed and local-only models; positive values indicate the overall gain of the transport-informed model relative to the local-only model. Panels **a** and **b** share the ΔR^2 color scale. **c**, Total upwind contribution: for each target grid cell, the fraction of predictive attribution originating from all upwind terrestrial grid cells, computed as the summed absolute attribution over upwind cells normalized by the summed absolute attribution over all input grid cells in its 21×21 window (a value of 100% indicates complete dependence on upwind information). Upwind cells are identified with UTrack (**Methods**); predictive attribution is from expected gradients (**Methods**). The geographic base map in a, b and c, including coastlines and country borders, is generated using the public-domain Natural Earth dataset via Python's Cartopy library.

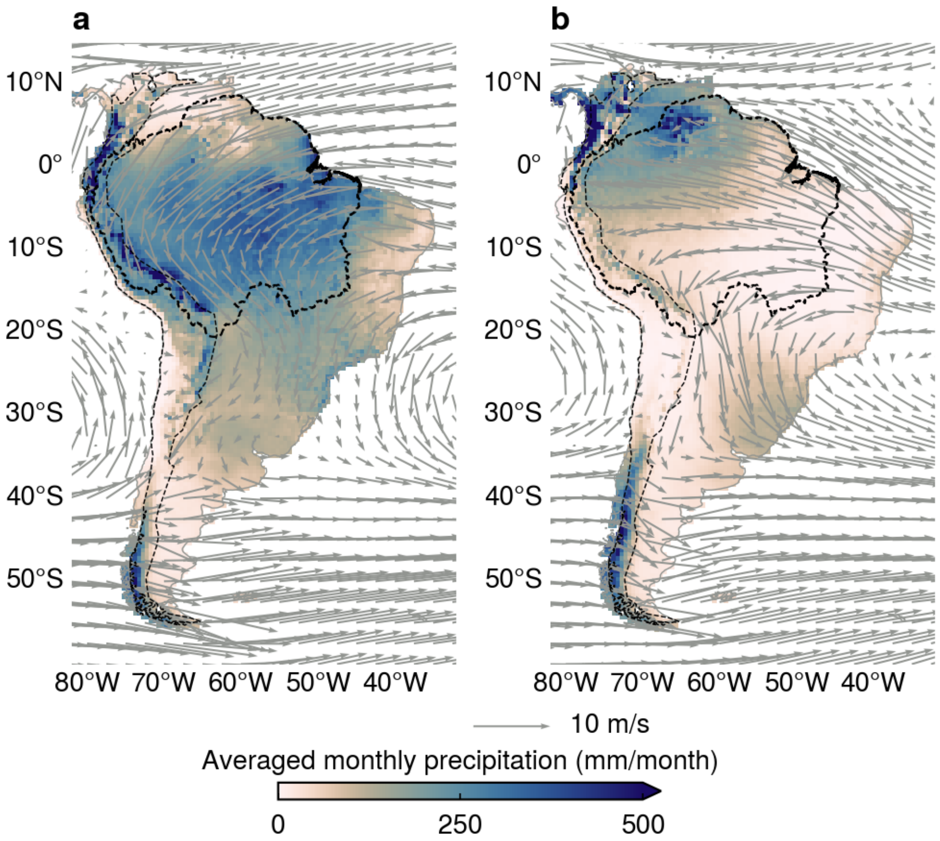

Fig. S7 | Spatial structure of average monthly precipitation across South America.

a, Average monthly precipitation in January. **b**, Average monthly precipitation in July. The precipitation is obtained from ERA5 and averaged over 2001–2018. Arrows show climatological 850 hPa wind vectors from ERA5 reanalysis (2001–2018). The geographic base map in a and b, including coastlines and country borders, is generated using the public-domain Natural Earth dataset via Python's Cartopy library.

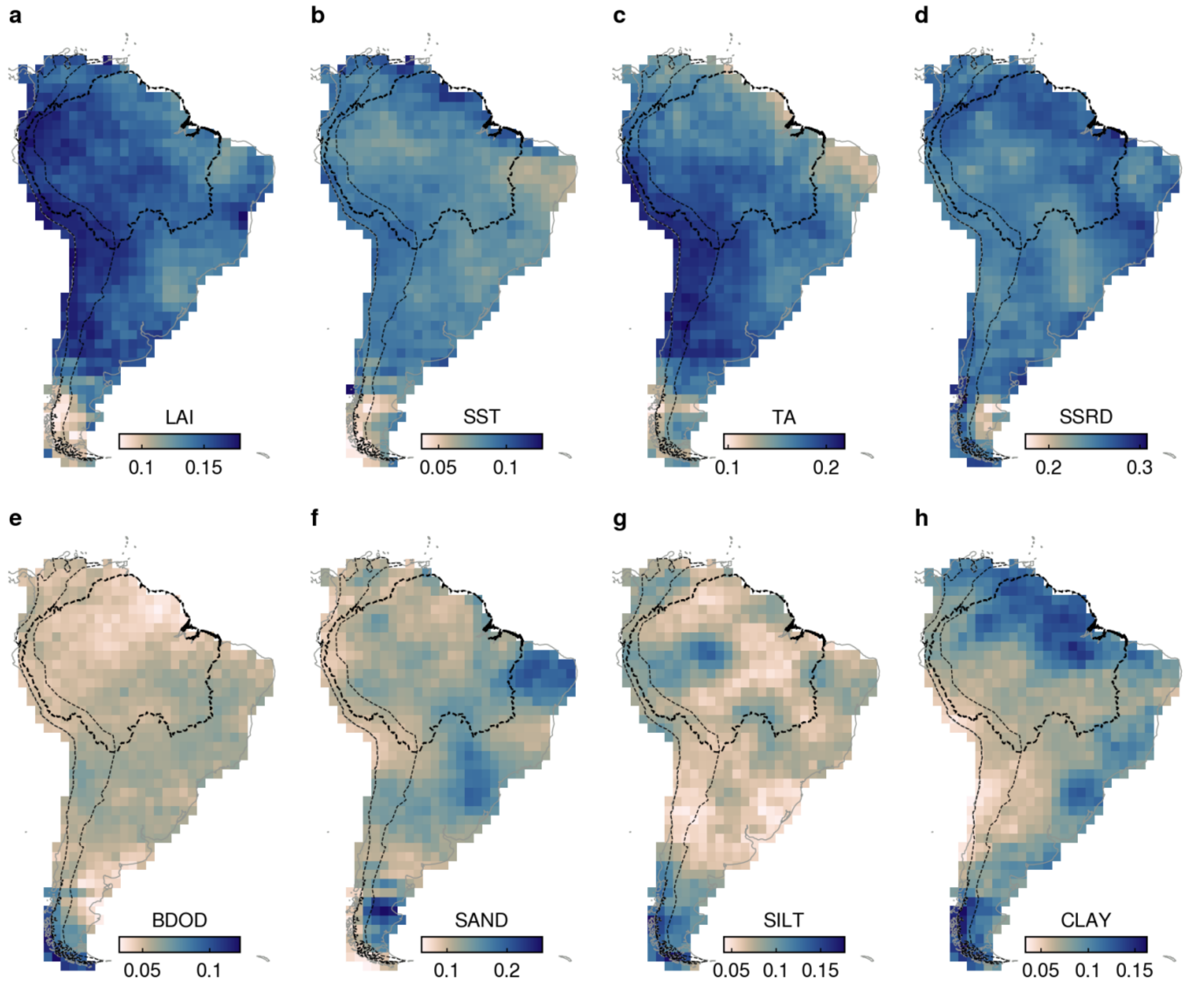

Fig. S8 | Variable-wise spatial distribution of predictive attribution within upwind grid cells in the transport-informed model.

Higher values indicate stronger dependence of soil moisture anomaly prediction on upwind information from corresponding variables. Shown are leaf area index (LAI, **a**), sea surface temperature (SST, **b**), air temperature at 2 m (TA, **c**), surface downward shortwave radiation (SSRD, **d**) anomalies, bulk density (BDOD, **e**), and sand (SAND, **f**), silt (SILT, **g**), and clay (CLAY, **h**) fractions. The geographic base map in a-h, including coastlines and country borders, is generated using the public-domain Natural Earth dataset via Python's Cartopy library.

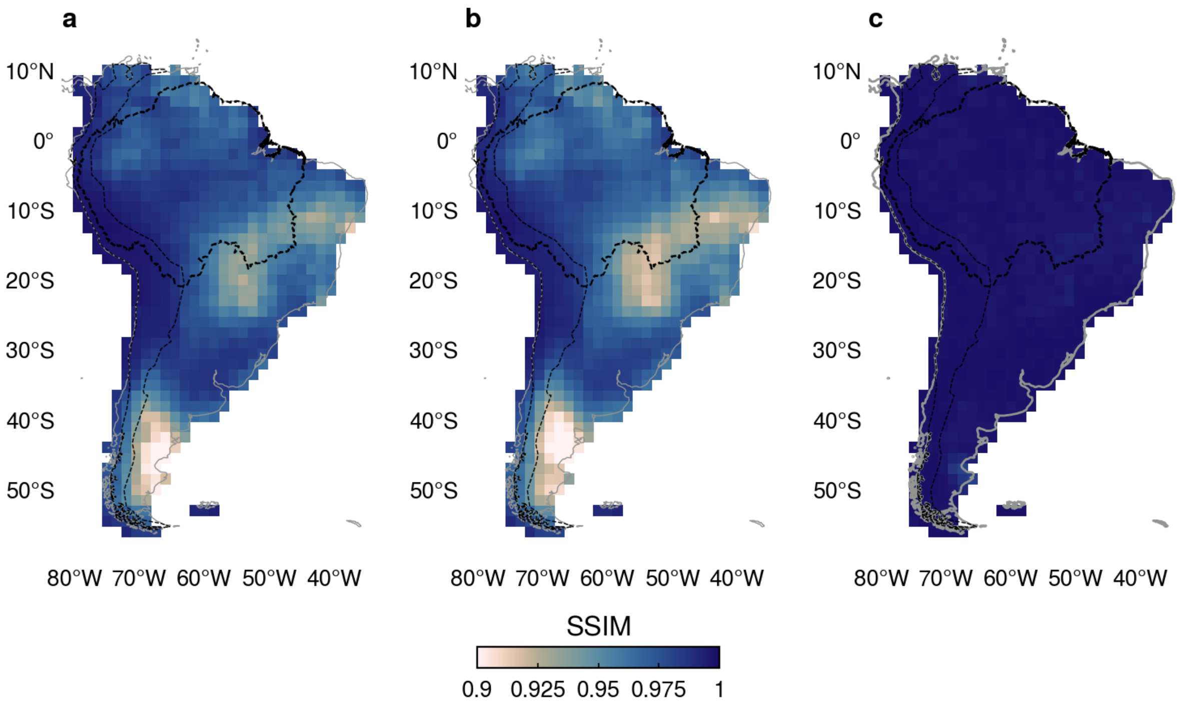

Fig. S9 | Structure similarity index metrics (SSIM) of integrated gradients (IG), GradientSHAP (GS), and expected gradients (EG) explanation.

a, SSIM of IG and GS; **b**, SSIM of IG and EG; **c**, SSIM of EG and GS. After obtaining the full set of explanation results from the three XAI methods, the SSIM is computed for each variable per pixel, and subsequently averaged to generate the spatial distribution maps. The geographic base map in a, b and c, including coastlines and country borders, is generated using the public-domain Natural Earth dataset via Python's Cartopy library.

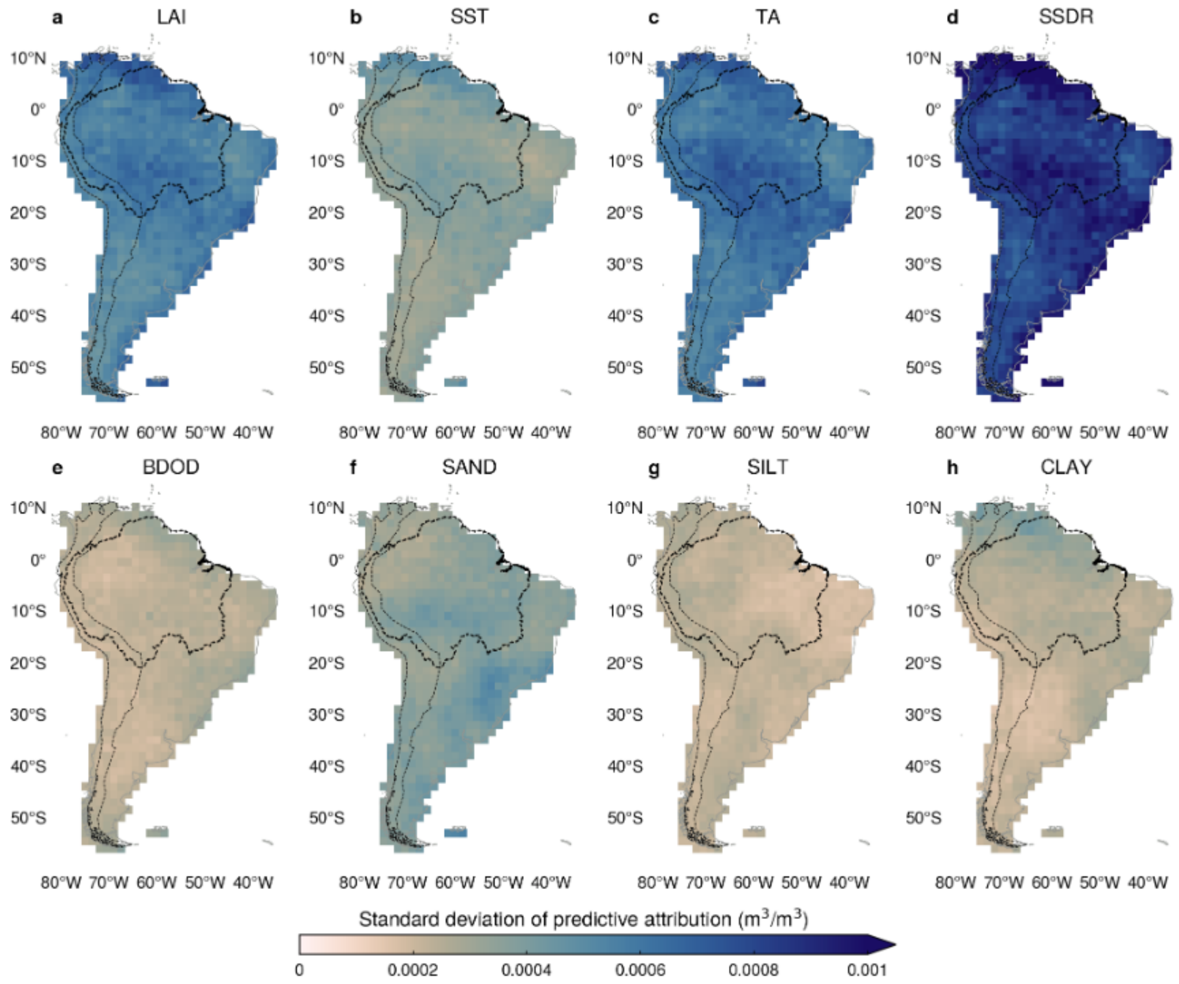

Fig. S10 | Standard deviation of the predictive attribution for eight input variables from four different trained models in four random seeds. The geographic base map in a-h, including coastlines and country borders, is generated using the public-domain Natural Earth dataset via Python's Cartopy library.

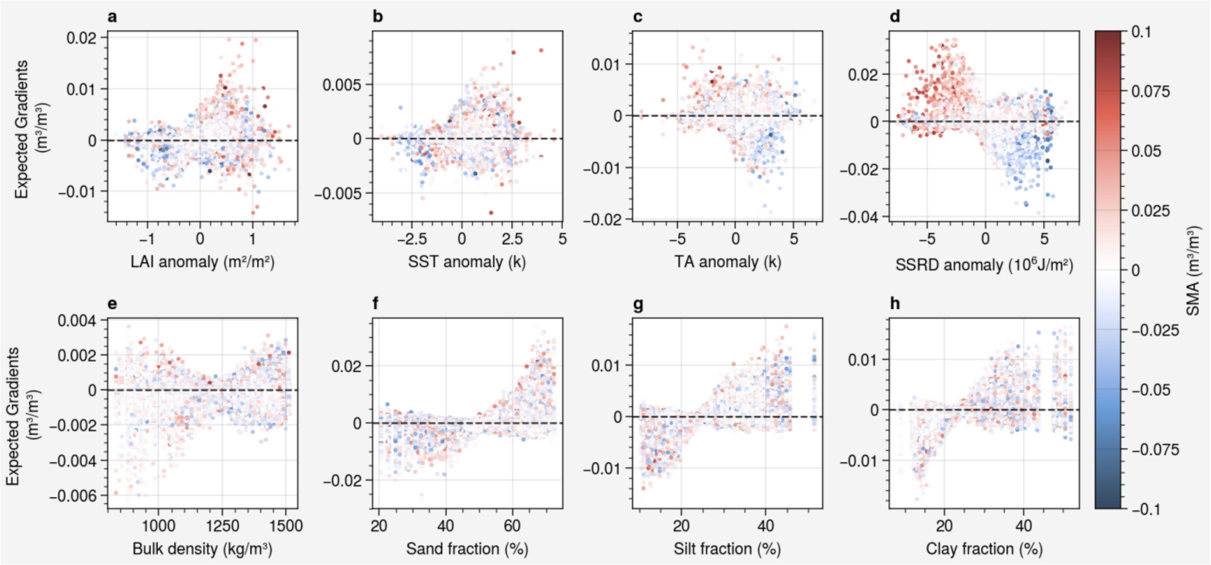

Fig. S11 | Directional dependence of predictive attribution (Expected Gradients) on input variables in transport-informed model.

Relationships between inputs (standardized anomalies) and expected gradients (EG) values for individual variables. Because both input variables and soil moisture anomaly (SMA) are expressed as anomalies, the sign of EG provides directional information, i.e., positive (negative) attribution values indicate that positive anomalies of the input variable are associated with increases (decreases) in predicted SMA relative to the climatological mean. The magnitude of EG reflects the local sensitivity of the prediction to perturbations in the input variable. Colors indicate the predicted SMA value. Panels show examples for (a) leaf area index (LAI), (b) sea surface temperature (SST), (c) near-surface air temperature (TA), (d) surface downward shortwave radiation (SSRD), (e) bulk density, and (f) sand, (g) silt, and (h) clay fractions.

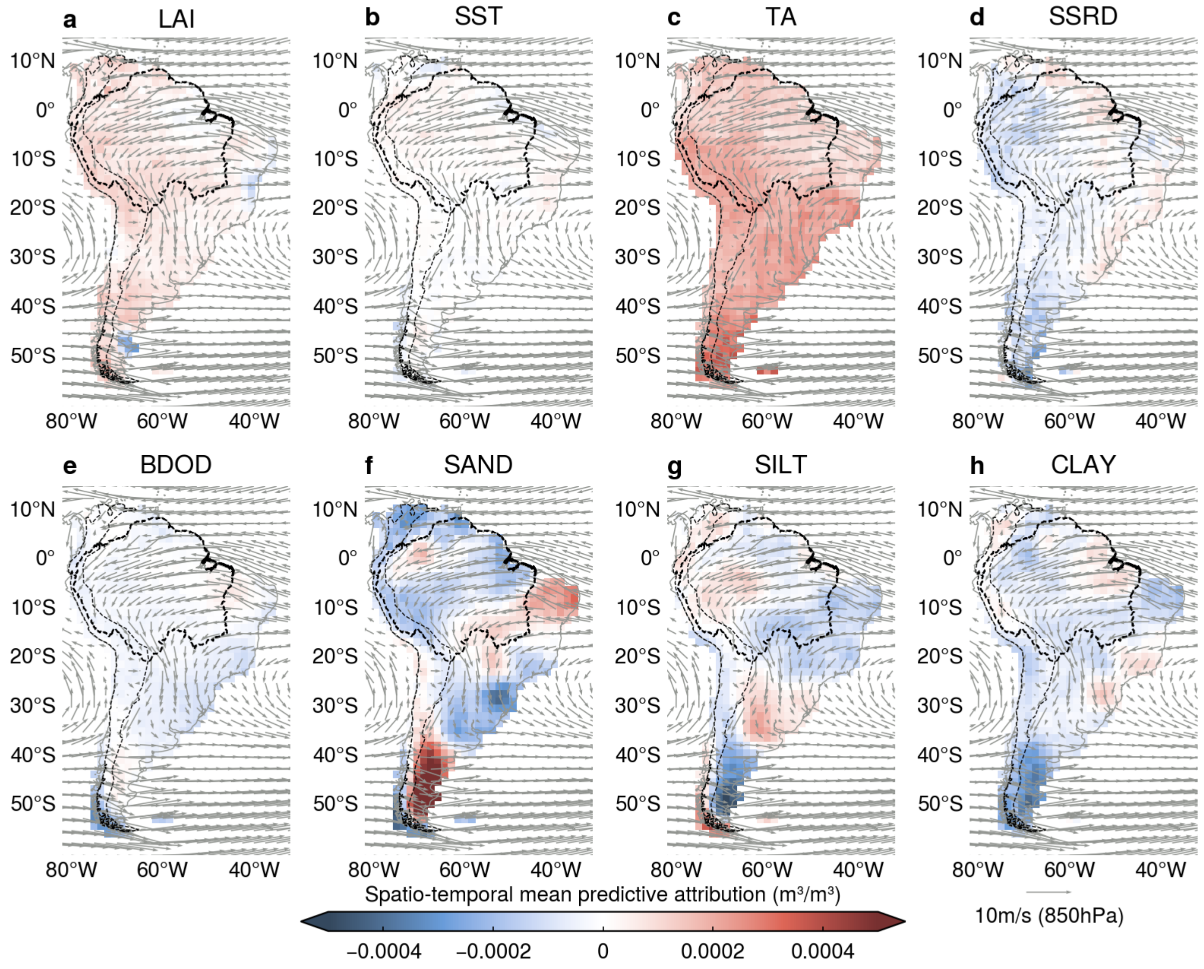

Fig. S12 | Spatial distribution of averaged predictive attribution (Expected Gradients, EG) for individual input variables.

The spatial map is derived by averaging the EG values of each variable's spatio-temporal inputs per grid cell and time step, followed by an overarching temporal averaging. The geographic base map in a-h, including coastlines and country borders, is generated using the public-domain Natural Earth dataset via Python's Cartopy library.

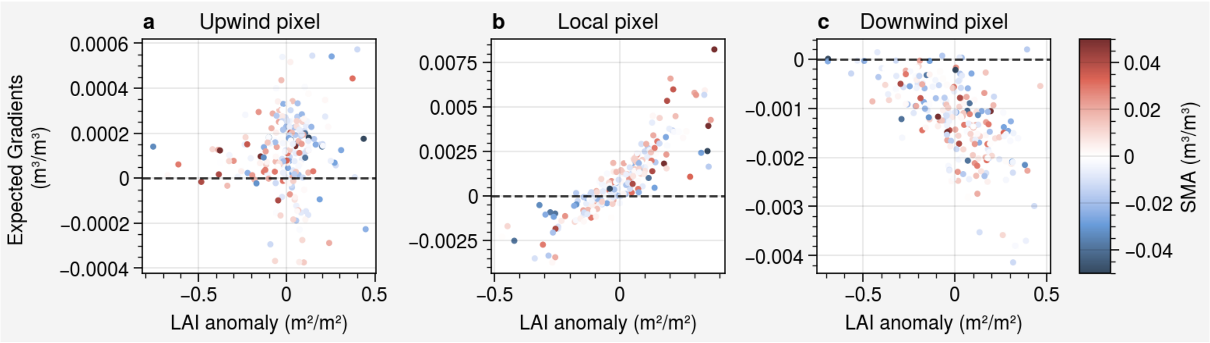

Fig. S13 | An example of nonlinear responses of predictive attribution (Expected Gradients, EG) to LAI anomalies.

Example prediction illustrating the relationship between LAI anomalies and EG attribution for the inputs for (**a**) upwind, (**b**) local, and (**c**) downwind pixels for a specific target grid cell, highlighting nonlinear and spatially dependent vegetation effects on predicted SMA.

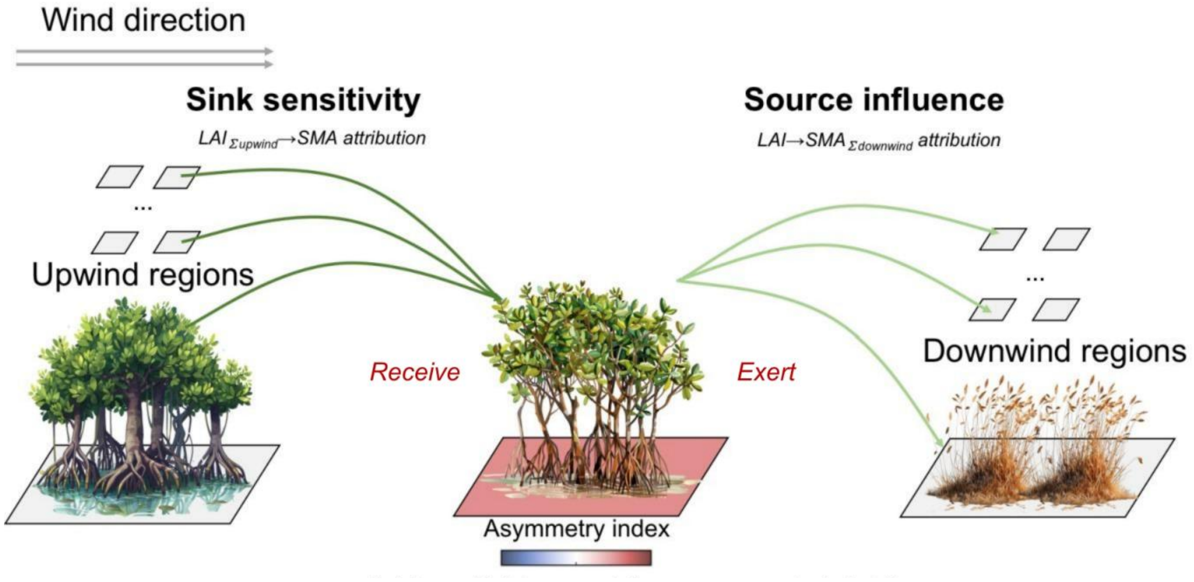

Fig. S14 | Schematic illustration of source influence, sink sensitivity, and the asymmetry index.

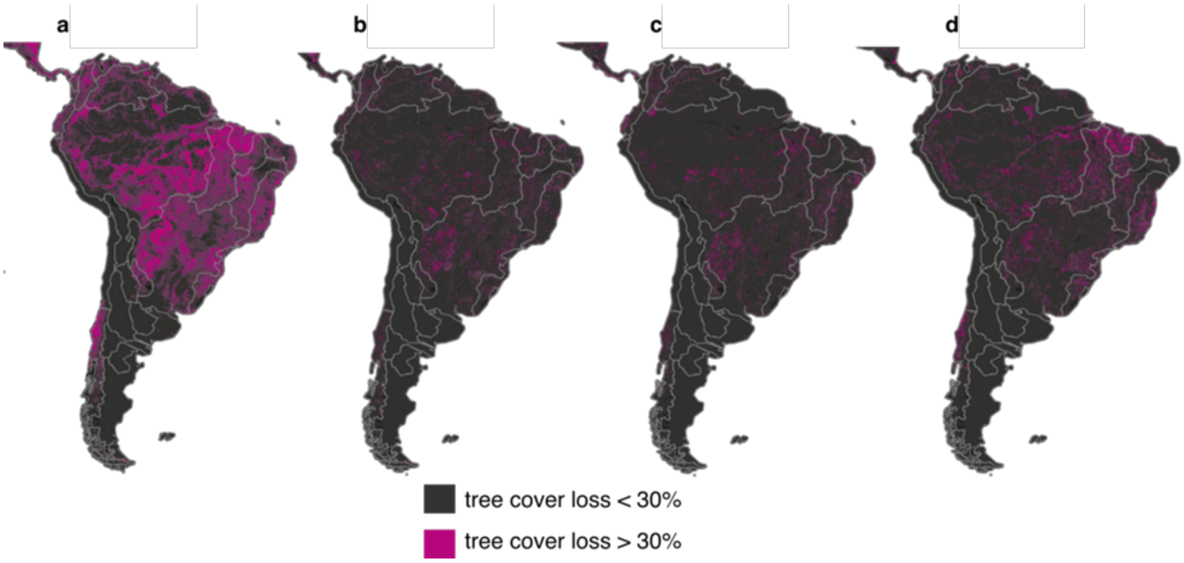

Fig. S15 | Tree loss area across South America for four historical periods.

a, 2001-2003; **b**, 2004-2008; **c**, 2009-2013; **d**, 2014-2018. The data is derived from Interactive World Forest Map & Tree Cover Change Data | GFW (https://www.globalforestwatch.org/map/). The geographic base map in a-d, including coastlines and country borders, is generated using the public-domain Natural Earth dataset via Python's Cartopy library.

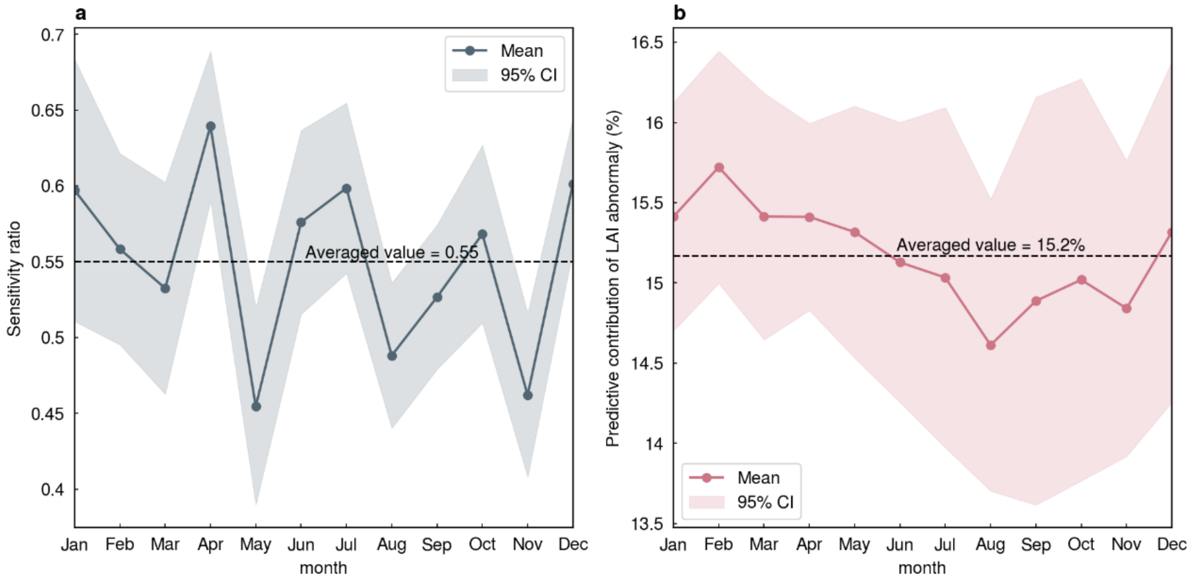

Fig. S16 | Sink sensitivity in cropland across South America.

a, Area-weighted averaging is applied to account for spatial heterogeneity in grid cell area. The proportion of LAI-dominant cropland cells is calculated and averaged monthly. Grid cells are classified as LAI-dominant if their LAI-derived sink sensitivity exceeds the median across the eight input variables (at that cell and month). **b**, Spatial and monthly averaging of LAI predictive contribution across cropland grid cells. Cropland data are from the GLAD cropland dataset. The data are aggregated to a 1.5° resolution and aligned with a land–sea mask. Cropland is defined as grid cells where the cropland fraction exceeds 10%.

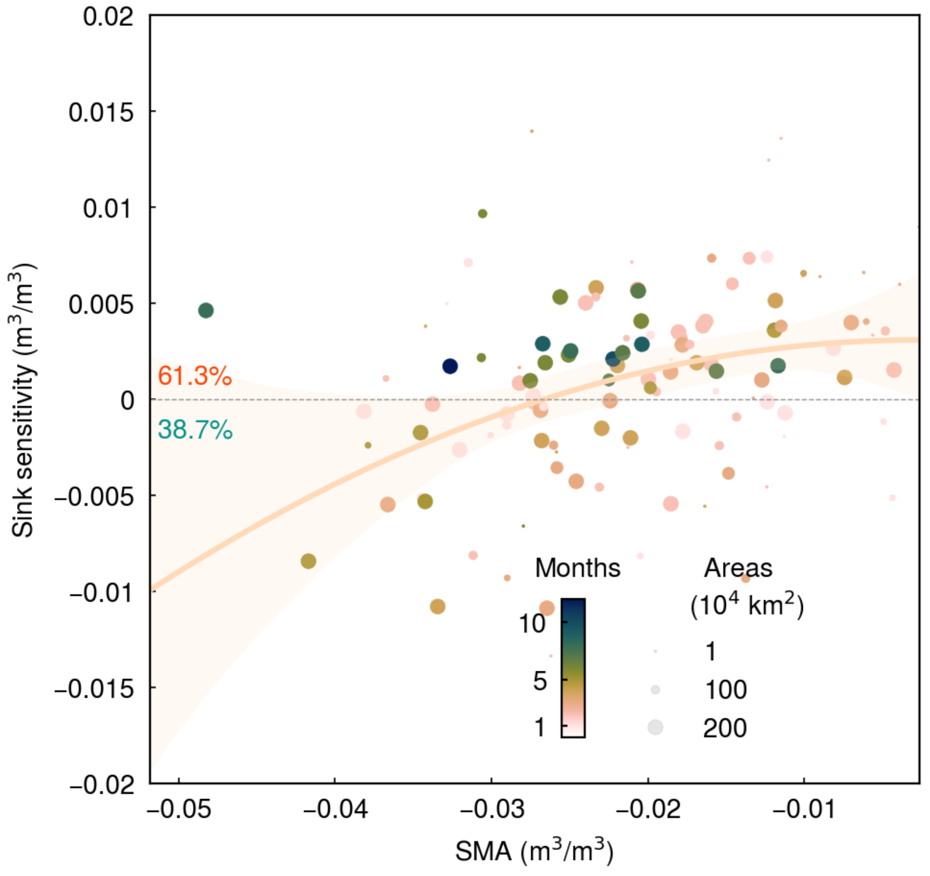

Fig. S17 | Event-based sink sensitivity of soil moisture droughts to upwind vegetation.

Event-averaged sink sensitivity versus mean SMA during 119 drought events from the SoMo.ml dataset (circles). Marker color denotes drought duration, and size denotes spatial extent. Positive sink sensitivity was observed in 61.3% of events.

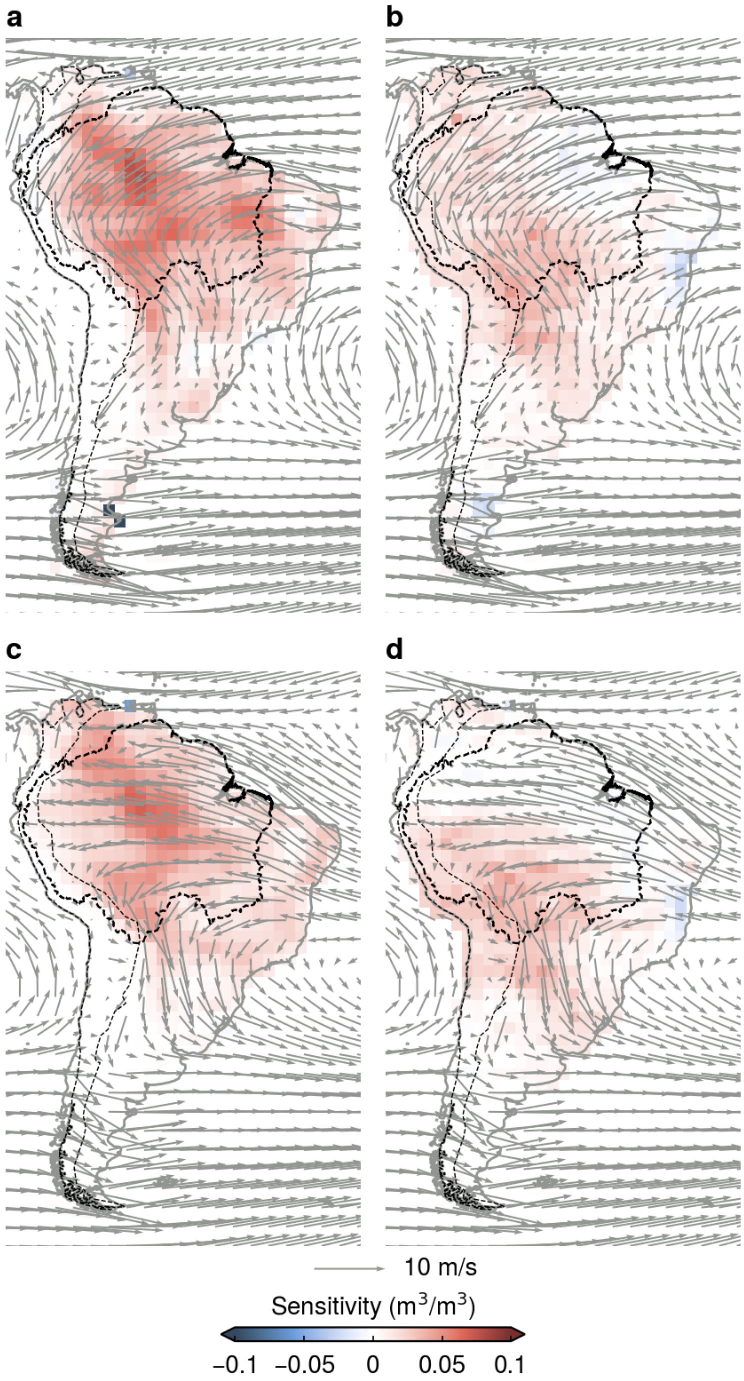

Fig. S18 | Comparison of sink sensitivity and source influence between growing season (DJF) and non-growing season (JJA) across South America.

The left panel compares the source influence in DJF (**a**) and JJA (**c**), and the right panel compares the sink sensitivity in DJF (**b**) and JJA (**d**). The geographic base map in a, b, c and d, including coastlines and country borders, is generated using the public-domain Natural Earth dataset via Python's Cartopy library.

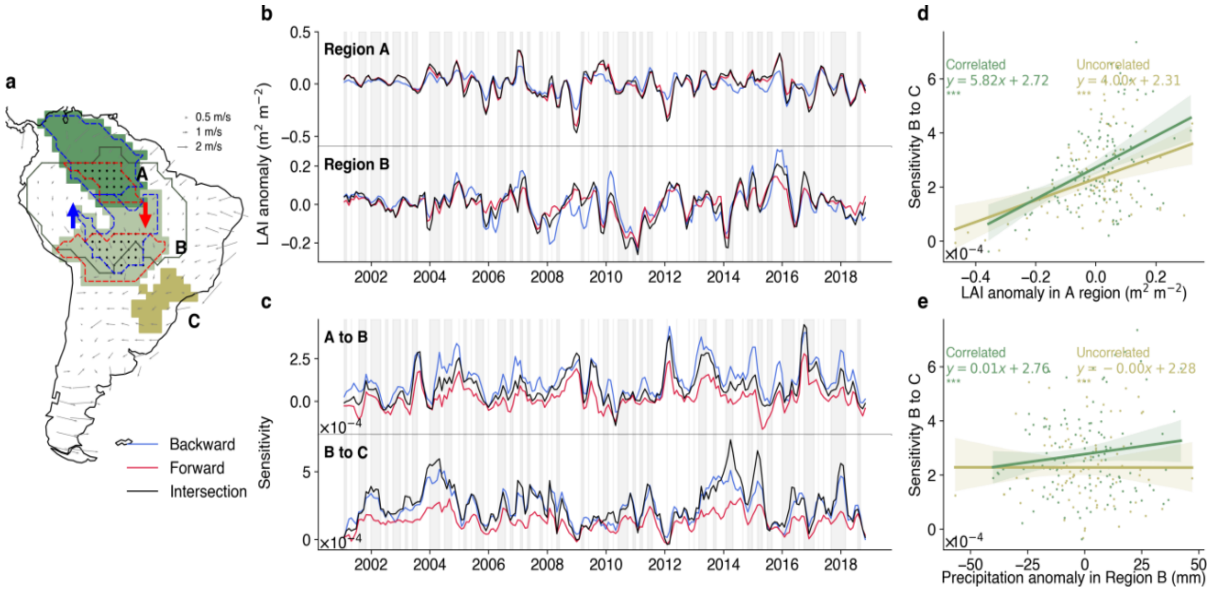

Fig. S19 | Cascade of Amazonia vegetation to soil moisture anomaly in the Brazilian agricultural zone.

a, Identified significant regions for cascade A (dark green)→B (light green)→C (orange). The area enclosed by the blue line is identified using the traceback approach (blue arrow), and the red line represents the trace-forward approach (red arrow), with the intersection areas marked as black dots. The wind plot is derived from monthly-averaged zonal and meridional wind data at 10 meters from 2001 to 2018. **b**, **c**, The 6-month rolling averaged time series of spatially averaged LAI anomalies of Region A and B (**b**) and sensitivity of A→B and B→C (**c**). The black line represents the intersection areas identified by the trace-forward approach (red line) and the trace-backward approach (blue line). **d**, Linear regression with LAI anomalies in Region A as input and sensitivity B→C as output. The green line denotes the linear regression result, with shading indicating the 95% confidence interval; green dots represent cases in which the A→B and B→C sensitivities are both present and correlated. Orange lines and dots denote the corresponding cases without this correlation. **e**, The same as **d**, but the linear regression input is precipitation in Region B. The geographic base map in a, including coastlines and country borders, is generated using the public-domain Natural Earth dataset via Python's Cartopy library.

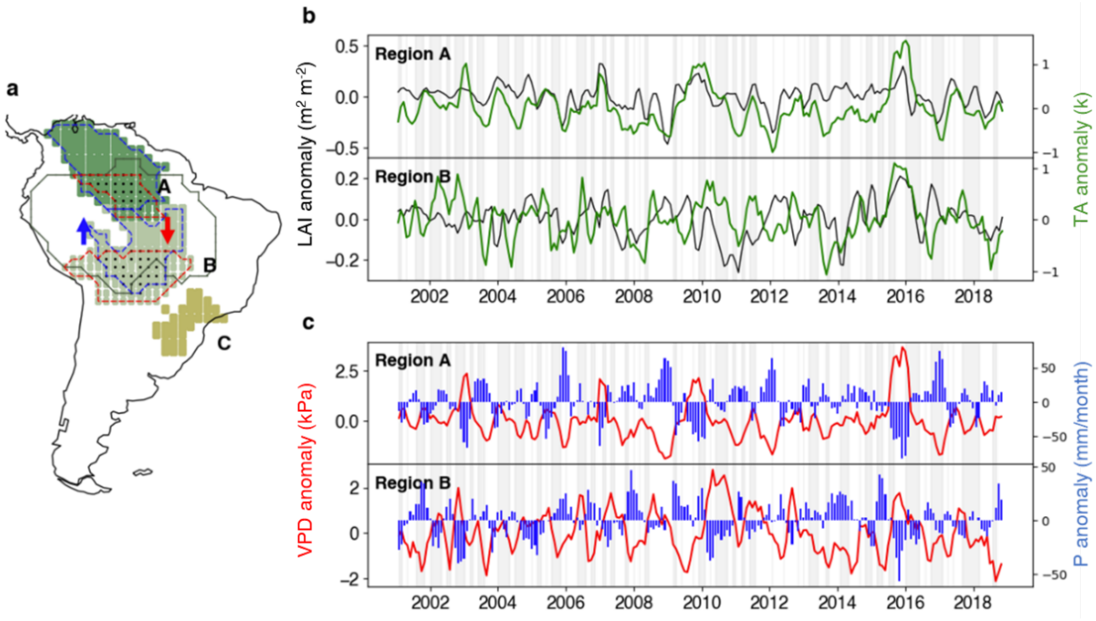

Fig. S20 | Meteorological anomalies and LAI anomalies in the cascade of Amazonia vegetation to soil moisture anomaly in the Brazilian agricultural zone.

a, Identified significant regions for cascade A (dark green)→B(light green)→C (orange). The area enclosed by the blue line is identified using the traceback approach (blue arrow), and the red line represents the trace-forward approach (red arrow), with the intersection areas marked as black dots. **b**,**c**, The 6-month rolling averaged time series of spatially averaged LAI anomaly and TA anomaly (b) and vapor pressure deficit (VPD) anomaly and precipitation (P) anomaly (**c**) in Region A and B. The geographic base map in a, including coastlines and country borders, is generated using the public-domain Natural Earth dataset via Python's Cartopy library.

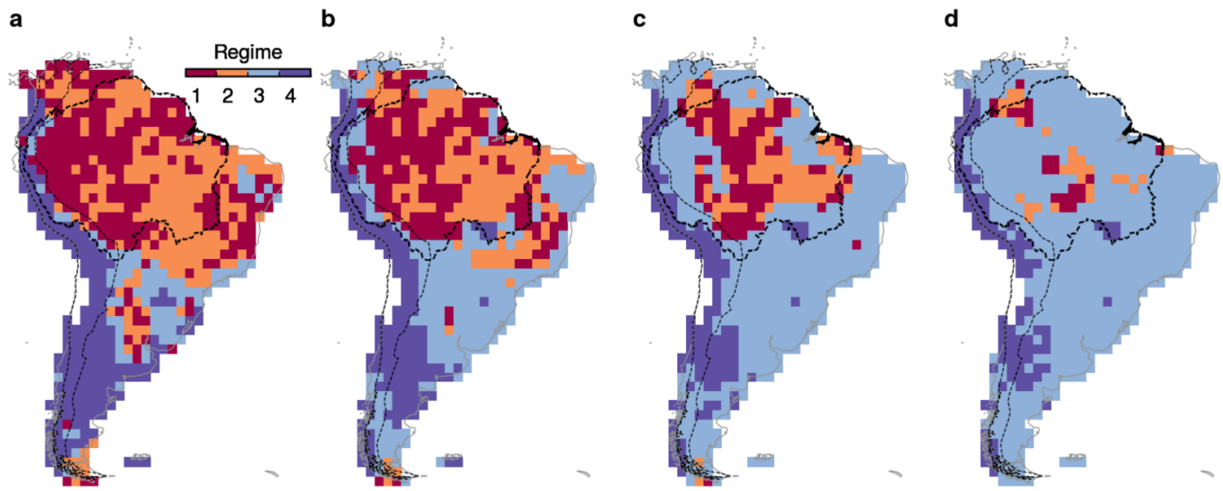

Fig. S21 | Sensitivity of regime classification to persistence thresholds.

Regimes are classified based on the fraction of months with positive source influence. The figure compares the resulting regime assignments using thresholds of 60% (**a**), 70% (**b**), 80% (**c**), and 90% (**d**), demonstrating that the main regime patterns are robust to the choice of threshold. The geographic base map in a-d, including coastlines and country borders, is generated using the public-domain Natural Earth dataset via Python's Cartopy library.

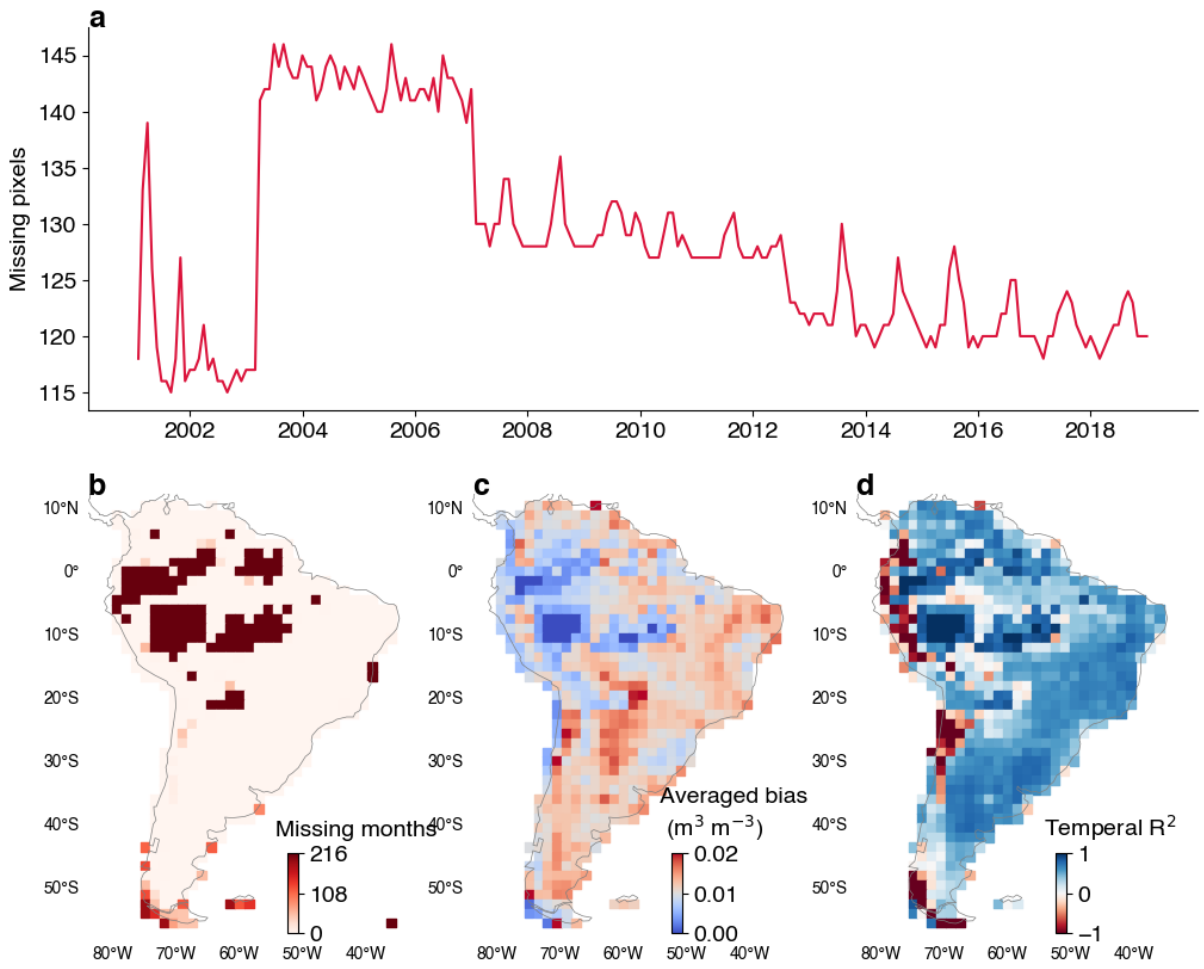

Fig. S22 | ESA CCI data completeness and validation metrics.

a, Temporal coverage by year highlighting periods with incomplete data. **b**, Spatial distribution of the number of missing months per grid cell. **c**, Temporally averaged bias in filled ESA CCI soil moisture with ERA5-Land compared to SoMo.ml (m³ m⁻³). **d**, Temporal R^2 correlation coefficient between filled ESA CCI and SoMo.ml ranging from -1 to 1, where values approaching 1 indicate stronger agreement between observed and reference data. The geographic base map in b-d, including coastlines and country borders, is generated using the public-domain Natural Earth dataset via Python's Cartopy library.

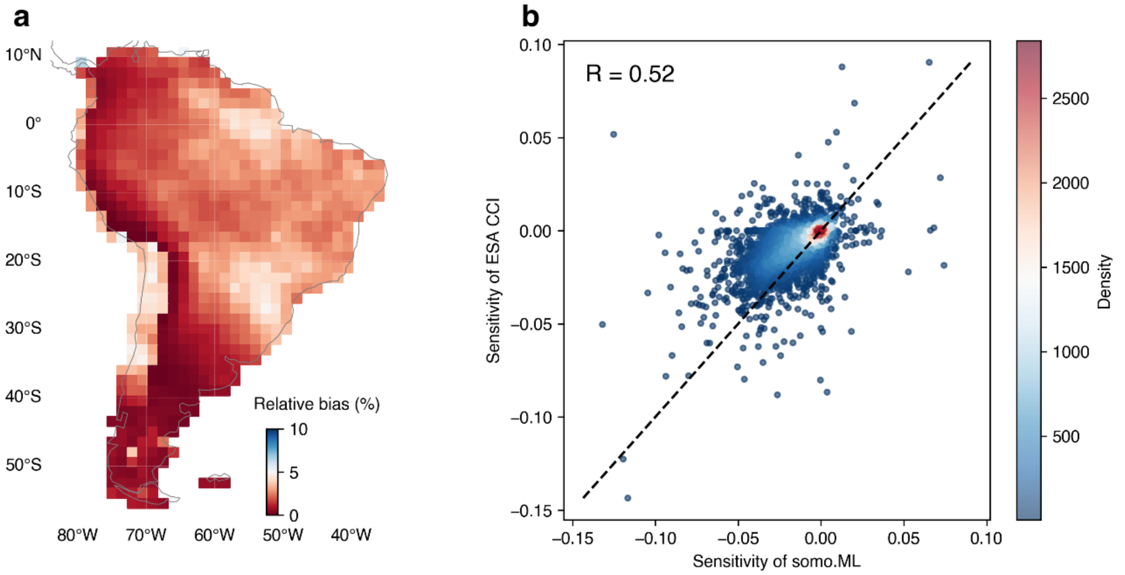

Fig. S23 | Comparable analysis of sensitivity derived from SoMo.ml and ESA CCI.

a, Relative bias between sink sensitivity derived from SoMo.ml and ESA CCI. The relative bias is calculated by the percentage of sink sensitivity difference accounting for the range of sink sensitivity from SoMo.ml. **b**, The distribution and density of sensitivity of SoMo.ml and ESA CCI. The Pearson coefficient (R) is 0.52 between the two sensitivities derived from the two datasets. The geographic base map in a, including coastlines and country borders, is generated using the public-domain Natural Earth dataset via Python's Cartopy library.

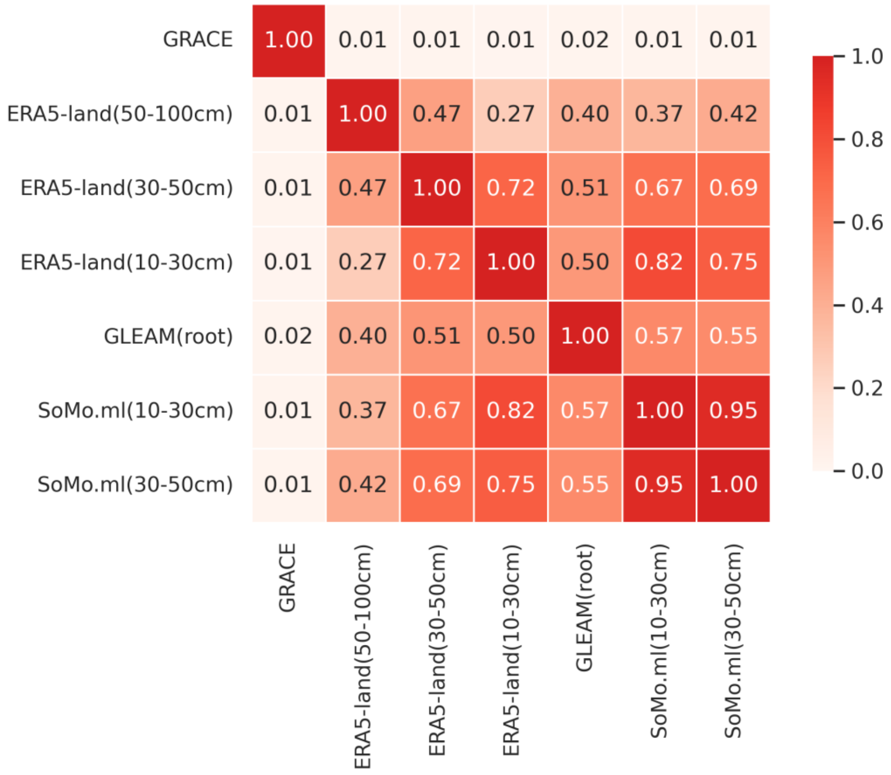

Fig. S24 | Pearson correlation of different sources of subsurface soil moisture anomaly data.

Data are obtained from GRACE (grace.jpl.nasa.gov), ERA5-Land (cds.climate.copernicus.eu), SoMo.ml (nature.com/articles/s41597-021-00964-1), and GLEAM (gleam.eu). For ERA5-Land, subsurface soil moisture data at 10-30 cm, 30-50 cm, and 50-100 cm layers were obtained through linear interpolation of the original levels.

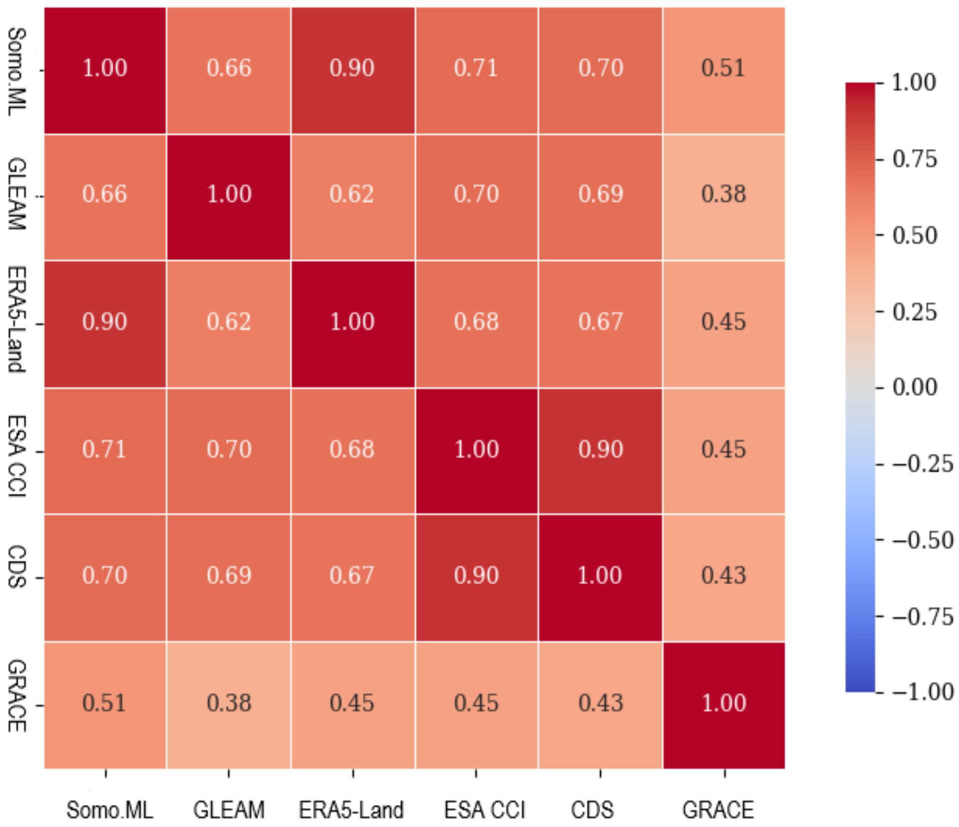

Fig. S25 | Pearson correlation of different sources of surface soil moisture anomaly data.

Data are obtained from GRACE (grace.jpl.nasa.gov), ERA5-Land (cds.climate.copernicus.eu), SoMo.ml (nature.com/articles/s41597-021-00964-1), and GLEAM (gleam.eu). Copernicus Climate Change Service Climate Data Store (CDS) soil moisture is from https://climate.copernicus.eu/land-hydrology-cryosphere. For ERA5-Land and CDS, surface soil moisture data at 0-10 cm layers were obtained through linear interpolation of the original levels.

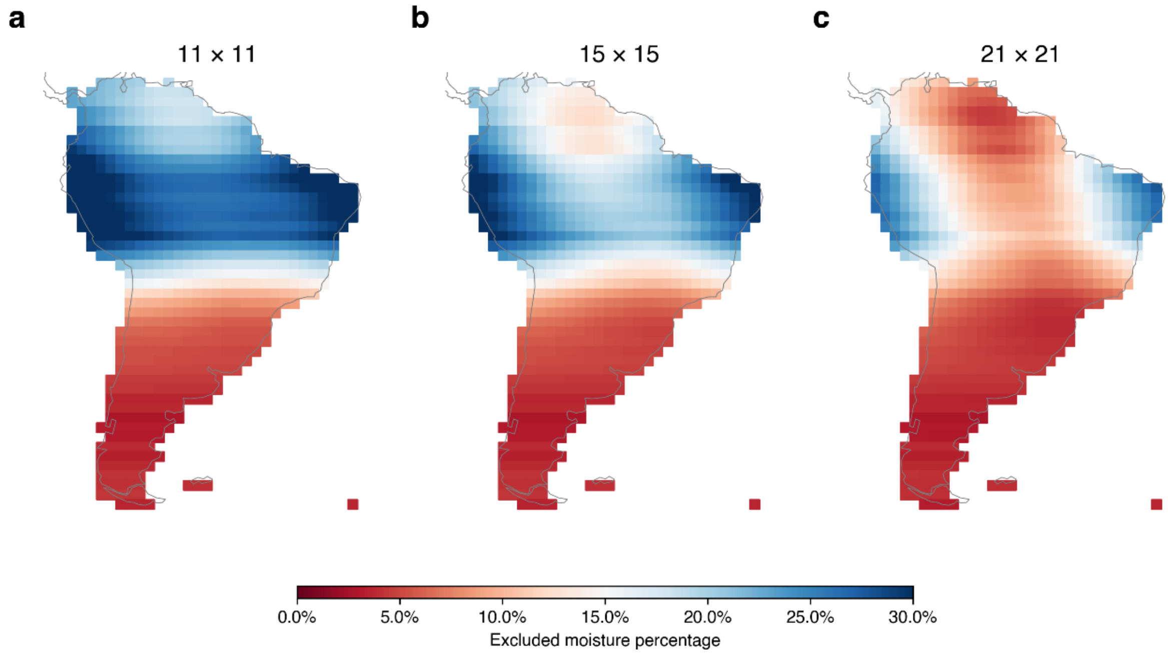

Fig. S26 | The proportion of terrestrial water vapor sources outside the defined window.

a, 11×11 grid cells. **b**, 15×15 grid cells and **c**, 21×21 grid cells. The geographic base map in a, b and c, including coastlines and country borders, is generated using the public-domain Natural Earth dataset via Python’s Cartopy library.

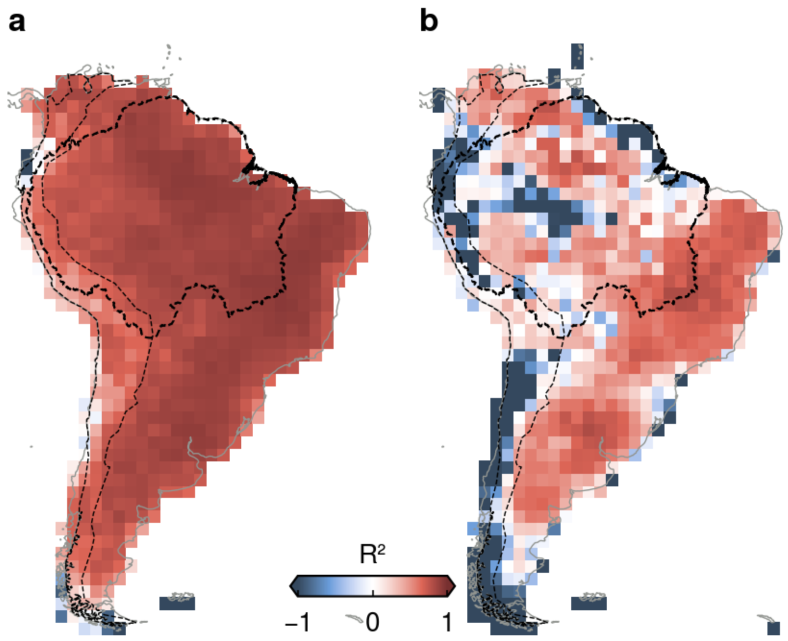

Fig. S27 | Model performance (R^2 values) of deep learning model with the UTrack data as one of the input variables based on two different soil moisture datasets.

a, Spatial R^2 values of the deep learning model based on SoMo.ml soil moisture dataset. The spatial R^2 values are the mean spatial R^2 values of the four settings of the initial model. **b**, the same as a, but for the ESA CCI dataset. The geographic base map in a and b, including coastlines and country borders, is generated using the public-domain Natural Earth dataset via Python's Cartopy library.

Supplementary References

1. Dhillon, A.; Verma, G.K. Convolutional neural network: A review of models, methodologies, and applications to object detection. Prog. Artif. Intell. 9, 85–112 (2020).

2. Li, H., Yue, X. & Meng, L. Enhanced mechanisms of pooling and channel attention for deep learning feature maps. PeerJ Computer Science 8, e1161 (2022).

3. Erion, G., Janizek, J. D., Sturmfels, P., Lundberg, S. M. & Lee, S.-I. Improving performance of deep learning models with axiomatic attribution priors and expected gradients. Nat Mach Intell 3, 620–631 (2021).

4. Lundberg, S. M. & Lee, S.-I. A unified approach to interpreting model predictions. In Advances in Neural Information Processing Systems 30 (2017).

5. Sundararajan, M., Taly, A. & Yan, Q. Axiomatic attribution for deep networks. Proceedings of Machine Learning Research 70, 3319–3328 (2017).

6. Wang, Z., Bovik, A. C., Sheikh, H. R. & Simoncelli, E. P. Image quality assessment: from error visibility to structural similarity. IEEE Trans. on Image Process. 13, 600–612 (2004).
