# Supplementary material for: Upwind terrestrial influences on soil moisture variability across South America
Source: Nat Commun. 2026 Jul 24;17:7256. doi: 10.1038/s41467-026-75637-x (PMC13400732; doi:10.1038/s41467-026-75637-x)
Supplement: Supplementary file 2 — Transparent Peer Review file [file 41467_2026_75637_MOESM2_ESM.pdf]

nature portfolio

Peer Review File

Upwind terrestrial influences on soil moisture variability across South America

Corresponding Author: Dr Shijie Jiang

This file contains all reviewer reports in order by version, followed by all author rebuttals in order by version.

Version 0:

Reviewer comments:

Reviewer #1

(Remarks to the Author)
Thank you for inviting me to review the paper: “Vegetation dynamics modulate downwind soil moisture across South America” by Huang et al.

This is a very interesting paper, as it explores how two factors—upwind moisture transport and different vegetation cover—affect soil moisture across South America. One strength of the manuscript is its use of quite “calm” scientific language. However, it also has clear implications for two main environmental issues affecting the Amazon: (1) climate change and (2) deforestation. It might be beneficial to highlight these points early on, possibly even in the Abstract. For example, it could state: “…a share that is significantly enhanced in agricultural zones, which may represent areas of deforestation.” This is especially important because researchers often initially judge papers based on the Abstract alone.

The title feels odd? According to the authors’ statistics in the Abstract, 67% of the predictive model for soil moisture relies on upwind information, with only 15% explained by vegetation. Should the title be made broader? (also see comment ** below – at first glance, the impression is that vegetation dynamics might be a local influence – and upwind is simply inbound moisture as an additional forcing).

I really appreciate the focus on atmospheric teleconnections, and I would say there has always been significant interest in these throughout my career as a climate researcher. They are, of course, challenging to define—linking different locations by definition, sometimes across seasons, and involving varying quantities at the start/end of a teleconnection. I imagine this could be a promising area for additional machine learning applications—beyond the “spatial context model” - used to identify other South American teleconnections. In this case, the authors have relied on their intuition, which has been validated by data showing a strong soil moisture dependence on upwind moisture transport. The authors might consider acknowledging that modern methods could further refine these findings. Otherwise could apply more subtle AI/ML techniques to extend the analysis presented here.

While I trust that the analysis is good, and reproducible, the presentation could be substantially improved in order to remove any risk of ambiguity. Figure 1 is especially critical to the paper, but I am really struggling to fully understand what it represents – despite reading the caption multiple times. This is not helped by the caption being incomplete, and potential some of what should be in the caption appearing in the colourbar label.

Aiming for a keynote Figure 1, the starting point of the paper is that there are three models considered of (1) a local model (so accounting only for local rainfall and evaporation), but not accounting for upwind influence, (2) the upwind contribution included and based on a “spatial context” model (so AI-derived) and (3) upwind contribution using the process-based atmospheric transport model. Hence, in Figure 1, if I have understood correctly, panel (a) is the extra part of R^2 fit that is achieved with the AI-derived model compared to considering just local effects. Then panel (b) is the same, but using the physics-based atmospheric transport model. Hence, if the intention is to show the physics-based model does better, the authors might like to plot instead the difference in R^2 between the physics-based model and the AI-model – i.e. as panel b? That feels more logical to me, as it shows the progress (left-to-right) of better models.

The problem is, I cannot understand what panel c is showing at all. All the caption says is “Relative contribution of upwind regions to total feature importance”. However, the main map does not disaggregate by different features – although the

histogram inset does, of course. What is Figure 1c plotting?

What only becomes fully clear, when examining the inset of Figure 1c, is that disaggregating drivers into different factors actually relates to the upwind forcings. This needs to be emphasised more clearly in the introductory sections of the paper, because most might assume the study balances local factors (such as the local impact of LAI on soil moisture calculations) with inbound atmospheric moisture as simply another driver. In reality, the paper explores the significance of various terrestrial factors (inset) on the transported moisture from upstream. Please make it very clear that the paper is studying the terrestrial factors (inset Fig 1c) of the upstream contributions.

The reason the points made in the paragraph above are so important is that this really opens the way to using this analysis to understand the importance of land use change (deforestation) on soil moisture downstream. This could raise the prospect that some configurations of major deforestation might impact food security downstream.

I like Figure 2 partly because there are other papers that also examine atmospheric transport across South America – so it's helpful to see the mean flows displayed. For consistency, it would be good to include a reference back to Figure 1. One idea is to show the influence of all parameters in the inset (Figure 1c) as maps, like in Figure 2. Is SMA considered an aggregator of some of the non-LAI parameters listed in the inset?

I would like to understand the Asymmetry Index better. I appreciate there is an equation (3) in Methods, but I have found it hard to grasp its precise meaning. The text above Eqn (3) is also difficult to follow. (Additionally, if one does not refer to the Methods, there is a risk of misunderstanding from the caption of Figure 2. Although there is interannual variability, over a reasonable period, most locations will be in a near-equilibrium state, balancing import and export of moisture). To truly understand the contributions to the "Asymmetry Index," a schematic in the Methods might be helpful.

The caveats could be elaborated further. I always value "one paper, one message," and this manuscript clearly adopts this approach to identify features of South American soil moisture from 2008 to 2017, emphasising upwind and vegetation dynamics. Such detailed focus enables a better regional understanding compared to, for example, analysing ESM outputs. However, for this analysis to be truly useful, it should be considered within the broader context of climate change and deforestation. Would the authors be willing to extend the caveats or discussion section of the paper to acknowledge this, and offer specific recommendations? For example, future research could compare ESM projections of soil moisture for South America for the contemporary period. Does this reduce the uncertainty bounds for future soil moisture under a changing climate? Could the presented analysis serve as a data point in an emergent constraint across the CMIP6 ensemble? Do large-scale models also demonstrate the dependence on land-use and vegetation dynamics the authors identify in this data-led analysis, thus increasing their reliability for simulations involving significant deforestation?

In general, the presentation of the diagrams feels really rushed. Boxes are not closed around the panels, there is some ambiguity in places as to which panels the colourbars apply to, the panels are not lined up (they should be on either at least one dimension, horizontal or vertical), the font sizes seem in some places too large, elsewhere too small. If possible, please make the captions as complete as possible, given how people often export figures with captions for use in lectures and talks.

I am very happy to see this paper again. The overall impression is of an important piece of analysis, full of quite critical insights into the hydrological cycle of South America. However, the current version requires some further work to reach publication standards. I am confident that the authors can accomplish this, and I hope this happens.

(Remarks on code availability)

Reviewer #2

(Remarks to the Author)
This manuscript presents a well-structured analysis of how upwind vegetation dynamics influence downwind soil moisture across South America using a novel combination of deep learning and moisture-transport information. The study addresses an important scientific question, introduces a creative methodological framework, and provides continent-scale insights. Overall, the work is of high quality and potentially impactful, though several aspects would benefit from clarification before acceptance.

Here are some comments:

The regime classification is based on two criteria: 1) the persistence of the sign of source influence (e.g., >70% positive months), and 2) the shape of the piecewise linear regression between LAI anomaly and source influence. Have the authors tested the other thresholds, like 80% or 90%? Moreover, I suggest briefly summarizing the key steps in the main text, particularly the role of sign persistence and breakpoint-based regression, to improve clarity and readability.

The interpretation of regime 2 and 3 is interesting but underexplained. These two regimes represent the most complex and climatically heterogeneous parts of the continent, so I encourage the authors to provide a clearer mechanistic discussion for these two regimes.

While the attribution analysis using Expected Gradients provides spatially resolved importance scores, it does not inform the

directionality or nonlinear shape of input variables' influence on soil moisture anomaly. For example, how does increasing LAI or temperature affect predicted soil moisture?

The analysis is based solely on surface soil moisture (0–10 cm). However, deeper soil layers (e.g., 10–100 cm) are crucial for plant water uptake, particularly in seasonally dry regions. Could the author discuss whether considering only shallow soil moisture would influence attribution analysis? If deeper soil layers were considered and excluded, please clarify why.

(Remarks on code availability)

Version 1:

Reviewer comments:

Reviewer #1

(Remarks to the Author)
Thank you for asking me to rereview paper: "Upwind terrestrial influences on soil moisture variability across South America" by Huang et al.

First, can I apologise for taking longer than the usual two weeks to return this review.

I continue to believe that this manuscript has important implications for agricultural resilience, as measured by changes in soil moisture, calculated downwind of the Amazon rainforest. What I particularly like is that, unlike other papers, the focus is applicable to both potential land-use drivers in the Amazon and climate change ("TA", Figure 1d).

I can see that the authors have responded extensively to the review comments, which is appreciated.

My only remaining problem with the paper is that, in places, it is still quite difficult to follow, which is a real shame given its important messages. Getting this right is particularly important for a generalist journal such as Nature Communications. Indeed, even environmental scientists, though very familiar with their own research area, still need to be informed when considering other aspects of large-scale environmental change.

If I can give an example, that may improve the presentation of analysis in Figure 3, and the related text and caption. Anything in the climate system that is nonlinear will always capture the interest of people, so this Figure is important.

(1) The first line of the caption correctly addresses the influence of vegetation on downwind soil moisture. To make this super clear, maybe change the "y"-label to something like "Source influence on downwind soil moisture (m3/m3)."

(2) Land-use change is likely to cause LAI decreases, and so this is why this diagram needs prominence. Please ensure that specific point is reiterated more strongly in the Discussion.

(3) The methodological part is tough to follow. For instance, I really wanted to understand the threshold for the four nonlinear types. The caption is quite technical? But in particular, I wanted to understand what "identified by a 70% temporal sign-persistance threshold….and post-breakpoint trend" actually means in a more general sense.

(4) To achieve (3), the reader is sent, correctly to "Methods". In the methods, the key part is "Definition of climatic regimes". Somehow the description there feels slightly incomplete. How is the binomial test applied, e.g. is the breakpoint a freely floating variable? The four regimes are described as "positive-saturating", "positive-saturating", "transition" and "negative". However, does this order of the cases relate to the order of panels b, c, d and e? If so, then state this, or annotate the panels. For instance, is panel 3e actually "negative". Maybe it is negative, but the diagram shows little change? The paper is very technical, and it is important that all information is made available. However, it also remains important that everything is written in an accessible way. For each technical point, extra words nearby something like "Hence, what this means is that…." all help.

(5) I'm assuming that every location falls into at least one of the four panels? Correct? So there is no "no change" option for ET as a function of LAI. Hence, even small negative gradients would fall in the "negative" case? (But if there is no "no change" option, how does that fit with the statistical thresholds of change set out in Methods?)

(6) The presentation of Figure 3 can be improved a little. Close the rectangular boxes of b-e, add some space around each. The background colours used, along with the colourbar, appear especially weak – although that could be my printer (I have a paper copy of the manuscript in front of me).

(7) Regarding the extra information of ET Percentile, the caption description is "ET percentile,… based on distribution of ET values within each individual grid cell". I think I understand this, but then why does the colourbar not stretch from 0% to 100%, if every location is represented in one of panels b-e? Apologies if I am missing something very obvious.

Figure 3 is important because in the humid regions, many locations are "red" (Figure 3a; regime "1"), and so Figure 3b shows that lowering LAI (e.g. deforestation or climate-driven "dieback") can substantially alter soil moisture downwind.

Please make sure the Discussion/Conclusions are incredibly clear and simple in the way they cite back to Figure 3.

In summary, the paper is near, and its findings are especially important – I can imagine this manuscript appearing in Nature Communications. But could the authors please make a final sweep through, checking that everything is as clear as possible to readers? Although the comments above are directed to Figure 3, please check the manuscript elsewhere for similar issues.

(Remarks on code availability)

Reviewer #2

(Remarks to the Author)
The authors have thoroughly addressed my previous comments, and the manuscript has improved a lot. I have no further comments and support publication.

(Remarks on code availability)

Open Access This Peer Review File is licensed under a Creative Commons Attribution 4.0 International License, which permits use, sharing, adaptation, distribution and reproduction in any medium or format, as long as you give appropriate credit to the original author(s) and the source, provide a link to the Creative Commons license, and indicate if changes were made.
In cases where reviewers are anonymous, credit should be given to 'Anonymous Referee' and the source.
The images or other third party material in this Peer Review File are included in the article's Creative Commons license, unless indicated otherwise in a credit line to the material. If material is not included in the article's Creative Commons license and your intended use is not permitted by statutory regulation or exceeds the permitted use, you will need to obtain permission directly from the copyright holder.
To view a copy of this license, visit https://creativecommons.org/licenses/by/4.0/

Response to Reviewer #1

Reviewer 1 (1): Thank you for inviting me to review the paper: "Vegetation dynamics modulate downwind soil moisture across South America" by Huang et al.

This is a very interesting paper, as it explores how two factors—upwind moisture transport and different vegetation cover—affect soil moisture across South America. One strength of the manuscript is its use of quite "calm" scientific language.

Response: We thank the reviewer for this positive assessment of the manuscript. The feedback has been helpful in improving the clarity and presentation of the manuscript. Detailed responses to specific points are provided below.

Reviewer 1 (2): However, it also has clear implications for two main environmental issues affecting the Amazon: (1) climate change and (2) deforestation. It might be beneficial to highlight these points early on, possibly even in the Abstract. For example, it could state: "…a share that is significantly enhanced in agricultural zones, which may represent areas of deforestation." This is especially important because researchers often initially judge papers based on the Abstract alone.

Response: We thank the reviewer for this suggestion. In the revision, we adjusted the Abstract and early introduction to more explicitly note the relevance of the identified downwind dependencies to land-use change and climate-related hydroclimatic shifts.

In the **Abstract**, we added: "*This downwind dependence has crucial implications for understanding the hydrological impacts of land-use change and climate change-driven shifts in vegetation.*" (**lines 22-24**), and "*... and for assessing the broader hydrological consequences of land-use change and climate changes, highlighting the critical role of ecosystems in shaping continental-scale land-climate interactions.*" (**lines 38-40**).

In the **Main**, we also emphasized the relevance of these findings under ongoing environmental change: "*This limitation is particularly consequential under climate change and rapid land-use change, as vegetation loss in key source regions (e.g., Amazon) may alter soil moisture regimes in downwind regions (e.g., Brazil agricultural zones) through atmospheric moisture transport.*" (**lines 59-62**).

Reviewer 1 (3): The title feels odd? According to the authors' statistics in the Abstract, 67% of the predictive model for soil moisture relies on upwind information, with only 15% explained by vegetation. Should the title be made broader? (also see comment ** below – at first glance, the impression is that vegetation dynamics might be a local influence – and upwind is simply inbound moisture as an additional forcing).

Response: We thank the reviewer for this comment. We agree that the original title could be misinterpreted. We therefore revised the title to more explicitly reflect the combined influence of

upwind terrestrial conditions on soil moisture variability, rather than vegetation alone. The revised title now reads "***Upwind terrestrial influences on soil moisture variability across South America***".

Accordingly, we adjusted the opening of the **Abstract** as follows: "*Soil moisture exhibits coherent spatial patterns that reflect systematic influences from upwind terrestrial conditions across South America. Yet, the extent to which such influences propagate downwind via atmospheric transport remains challenging to quantify.*" (**lines 20-22**).

Reviewer 1 (4): I really appreciate the focus on atmospheric teleconnections, and I would say there has always been significant interest in these throughout my career as a climate researcher. They are, of course, challenging to define—linking different locations by definition, sometimes across seasons, and involving varying quantities at the start/end of a teleconnection. I imagine this could be a promising area for additional machine learning applications—beyond the "spatial context model" - used to identify other South American teleconnections. In this case, the authors have relied on their intuition, which has been validated by data showing a strong soil moisture dependence on upwind moisture transport. The authors might consider acknowledging that modern methods could further refine these findings. Otherwise could apply more subtle AI/ML techniques to extend the analysis presented here.

Response: We thank the reviewer for highlighting this perspective. In the revised manuscript, we added a brief statement in the **Discussion** noting that alternative machine learning or network-based methods may help further characterize large-scale moisture connectivity patterns, which reads: "*While the present framework focuses on a transport-informed and observation-driven representation of vegetation-soil moisture teleconnections, future work could explore complementary data-driven or network-based approaches to further characterize large-scale moisture connectivity, e.g., network-based moisture analysis*[33] *and graph-based learning*[59] *methods.*" (**lines 318-322**).

Additional references:

33 Wunderling, N., Wolf, F., Tuinenburg, O. A. & Staal, A. Network motifs shape distinct functioning of Earth's moisture recycling hubs. Nature Communications 13, 6574, (2022).

59 Kawale, J. et al. A graph-based approach to find Teleconnections in climate data. Statistical Analysis and Data Mining: The ASA Data Science Journal 6, 158–179 (2013).

Reviewer 1 (5): While I trust that the analysis is good, and reproducible, the presentation could be substantially improved in order to remove any risk of ambiguity. Figure 1 is especially critical to the paper, but I am really struggling to fully understand what it represents – despite reading the caption multiple times. This is not helped by the caption being incomplete, and potential some of what should be in the caption appearing in the colourbar label.

Aiming for a keynote Figure 1, the starting point of the paper is that there are three models considered of (1) a local model (so accounting only for local rainfall and evaporation), but not accounting for upwind influence, (2) the upwind contribution included and based on a "spatial context" model (so AI-derived) and (3) upwind contribution using the process-based atmospheric

transport model. Hence, in Figure 1, if I have understood correctly, panel (a) is the extra part of R^2 fit that is achieved with the AI-derived model compared to considering just local effects. Then panel (b) is the same, but using the physics-based atmospheric transport model. Hence, if the intention is to show the physics-based model does better, the authors might like to plot instead the difference in R^2 between the physics-based model and the AI-model – i.e. as panel b? That feels more logical to me, as it shows the progress (left-to-right) of better models.

The problem is, I cannot understand what panel c is showing at all. All the caption says is "Relative contribution of upwind regions to total feature importance". However, the main map does not disaggregate by different features – although the histogram inset does, of course. What is Figure 1c plotting?

What only becomes fully clear, when examining the inset of Figure 1c, is that disaggregating drivers into different factors actually relates to the upwind forcings. This needs to be emphasised more clearly in the introductory sections of the paper, because most might assume the study balances local factors (such as the local impact of LAI on soil moisture calculations) with inbound atmospheric moisture as simply another driver. In reality, the paper explores the significance of various terrestrial factors (inset) on the transported moisture from upstream. Please make it very clear that the paper is studying the terrestrial factors (inset Fig 1c) of the upstream contributions.

The reason the points made in the paragraph above are so important is that this really opens the way to using this analysis to understand the importance of land use change (deforestation) on soil moisture downstream. This could raise the prospect that some configurations of major deforestation might impact food security downstream.

I like Figure 2 partly because there are other papers that also examine atmospheric transport across South America – so it's helpful to see the mean flows displayed. For consistency, it would be good to include a reference back to Figure 1. One idea is to show the influence of all parameters in the inset (Figure 1c) as maps, like in Figure 2. Is SMA considered an aggregator of some of the non-LAI parameters listed in the inset?

Response: We thank the reviewer for the detailed and constructive comments on **Figure 1**. We agree that, in the original version, the figure's presentation and caption were not sufficiently clear. In response, we have substantially revised **Figure 1** and the associated text to more clearly convey the structure, logic, and purpose of the analysis.

1) To reflect the stepwise incorporation of spatial and physical information, we revised **Figure 1b** to present the ΔR^2 between the transport-informed model and the spatial-context model, following the reviewer's suggestion.

2) To address the ambiguity surrounding panel c, we redefined **Figure 1c** to show a single, clearly defined quantity. The revised **Figure 1c** now maps the fraction of predictive attribution originating from upwind grid cells relative to total model attribution within a fixed spatial window. The original inset histogram has been moved to **Figure 1d**, which now reports the variable-wise fraction of predictive attribution to upwind inputs.

3) To avoid confusion, we replaced the term "feature importance" with "*predictive attribution*" throughout the manuscript and clarified that soil moisture anomaly (SMA) is the predicted response variable, not an aggregate of upstream inputs (e.g., **lines 120-121**, **123**, **133-134**, **137**, **139**, **144**, **146**, **148**, **159**, **510**, **518**, and **549**).

4) Because **Figure 1** focuses on aggregated attribution, we show the spatial patterns of variable-wise contributions in Supplementary **Figure S6** and reference this in the **Results** text: "*Within this upwind contribution, LAI accounts for 15% on average **(Fig. 1d)**, alongside contributions from upwind air temperature and shortwave radiation (spatial contribution see **Fig. S6**)*" (**lines 125-127**).

5) To address the concern that the study could be misinterpreted as balancing local factors against inbound moisture as a bulk driver, we clarified early in the **main** text that the analysis explicitly targets terrestrial signals embedded in transported moisture: "*This framework disentangles the terrestrial drivers including vegetation, climate, and surface properties embedded in upwind moisture transport, allowing us to attribute downwind soil moisture variability to specific upwind land-surface processes rather than to moisture transport alone.*" (**lines 82-85**).

6) In line with this clarification, we also noted that the attribution-based framework allows for examining how land-use change, including deforestation, may influence soil moisture conditions and potentially food security through non-local moisture transport in **Main** text. Specifically, we added in the **Main**: *"... and the potential downwind consequences of large-scale vegetation loss."* (**lines 92-93**).

In the **Discussion**, we further contextualized this point with recent literature: *"This perspective is consistent with recent evidence that land-cover change can exert remote hydroclimatic impacts through atmospheric transport, with effects that depend on spatial scale and may even reverse seasonally[22]."* (**lines 272-275**), and *"Building on these insights, our analysis provides a pathway to assess how land-use change, such as large-scale deforestation, may alter soil moisture conditions in downwind regions. Such non-local impacts are particularly relevant for understanding potential consequences for agricultural productivity and food security far from the location of land-surface change."* (**lines 275-279**).

7) Finally, following the reviewer's suggestion, we now explicitly reference Fig. 1 when introducing Fig. 2 in the text. Fig. 2 is now presented as a follow-up analysis motivated by the upwind vegetation dominant contribution identified in Fig. 1, as reflected in the revised opening sentence of the corresponding section: "*Motivated by the prominent contribution of upwind vegetation identified in **Fig. 1c** and **d**, we characterize the spatial structure of vegetation-soil moisture teleconnections by mapping source influence and sink sensitivity.* " (**lines 144-146**).

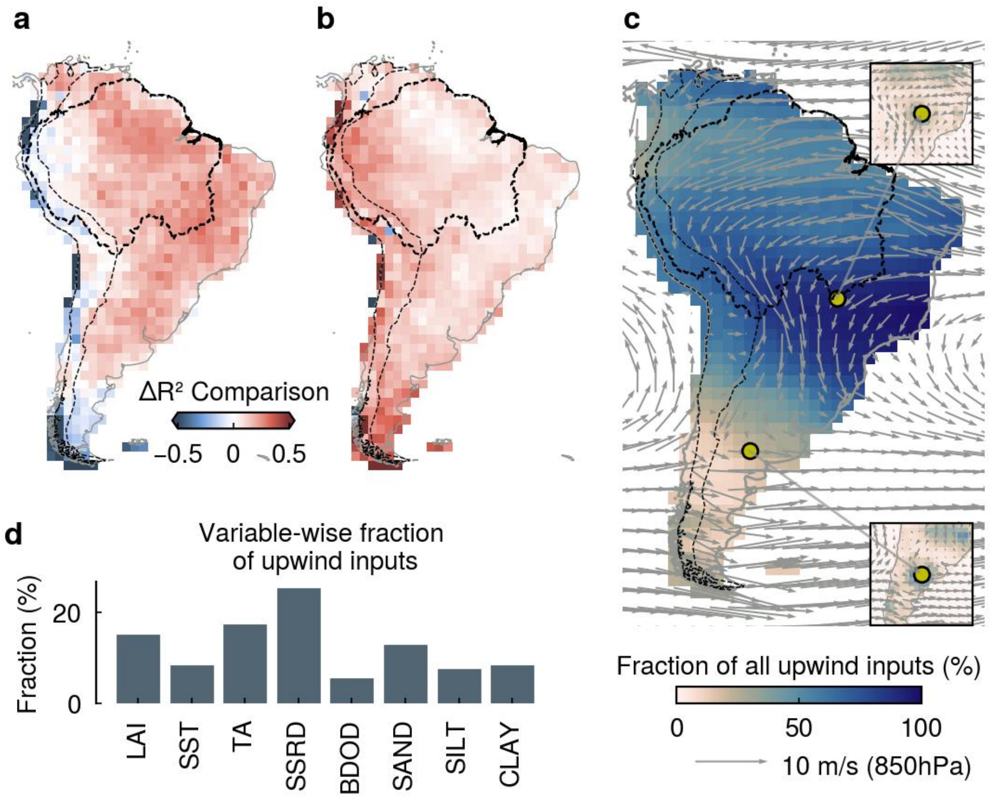

***Figure 1 | Performance gains of the transport-informed model and predictive attribution from land-surface and near-surface atmospheric inputs. a**, ΔR^2 between the spatial-context model and the local-only model. **b**, ΔR^2 between the transport-informed model and the spatial-context model. **c**, Fraction of predictive attribution originating from all upwind terrestrial grid cells. For each target grid cell, model inputs consist of a 21×21 surrounding window with eight variables. Predictive attribution is calculated using expected gradients[38] (**Methods**). The fraction is calculated as the sum of absolute attribution values across all variables for upwind grid cells, normalized by the sum of absolute attribution values across all input grid cells. A value of 1 indicates complete dependence on upwind information. Upwind grid cells are identified using UTrack[34] (**Methods**). The two insets illustrate the spatial distribution of predictive attribution across surrounding input terrestrial grid cells for representative single-grid-cell predictions (yellow points). The values shown in **Fig. 1c** correspond to the summed predictive attribution from upwind grid cells in the insets. Arrows indicate climatological 850 hPa wind vectors from ERA5 reanalysis for 2001-2018. **d**, Variable-wise fraction of predictive attribution to upwind inputs in the transport-informed model, shown as the fraction of total absolute attribution summed over all upwind grid cells. Spatial attribution maps for individual variables are shown in **Fig. S6**. Variables include leaf area index (LAI), sea surface temperature (SST), air temperature at 2 m (TA), surface downward shortwave radiation (SSRD), bulk density (BDOD), and sand (SAND), silt (SILT), and clay (CLAY) fractions.*

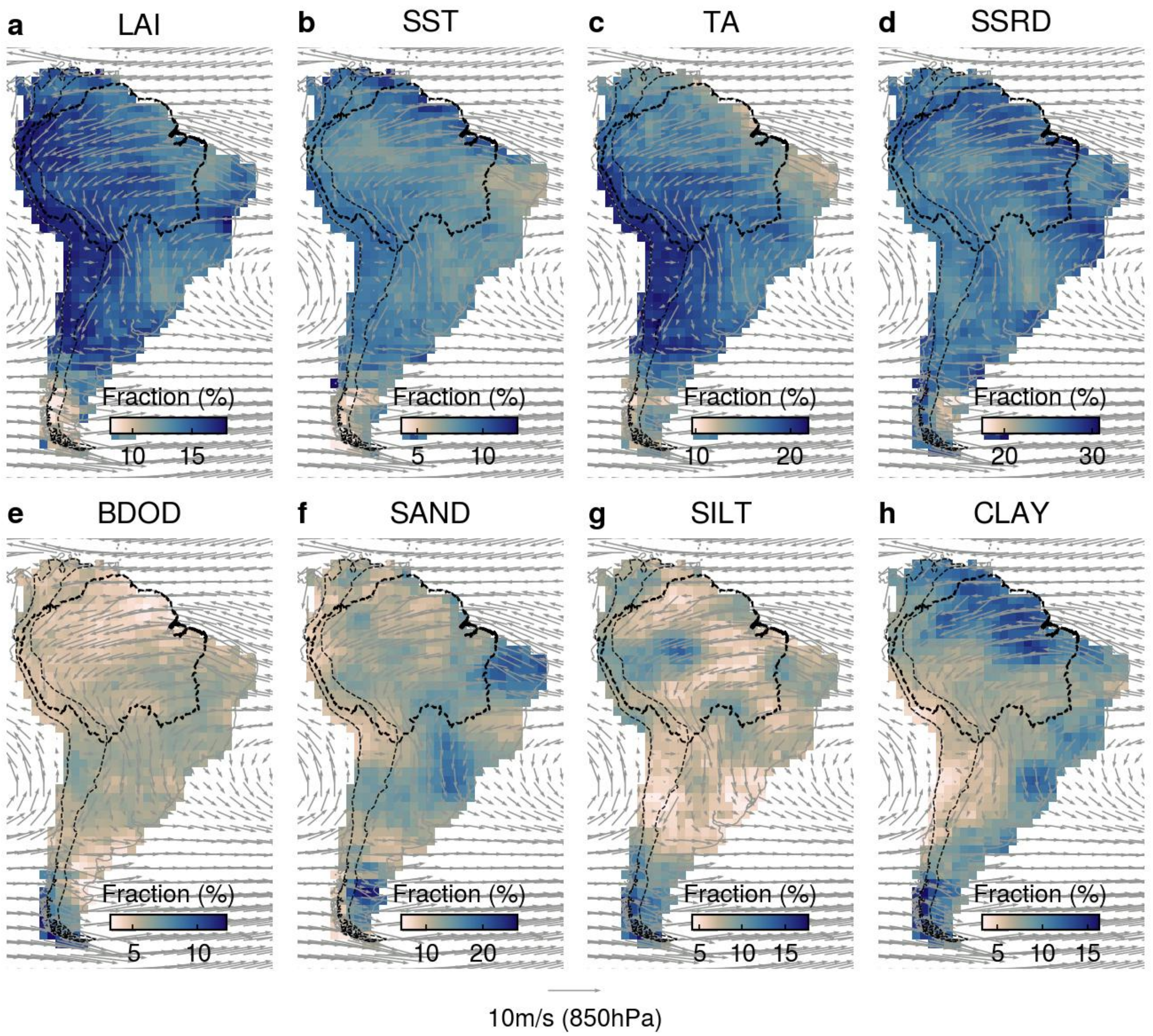

Fig. S6 | Variable-wise spatial distribution of predictive attribution within upwind grid cells in the transport-informed model.

*Higher values indicate stronger dependence of soil moisture anomaly prediction on upwind information from corresponding variables. Shown are leaf area index (LAI, **a**), sea surface temperature (SST, **b**), air temperature at 2 m (TA, **c**), surface shortwave radiation downward (SSRD, **d**), bulk density (BDOD, **e**), and sand (SAND, **f**), silt (SILT, **g**), and clay (CLAY, **h**) fractions.*

Reviewer 1 (6): I would like to understand the Asymmetry Index better. I appreciate there is an equation (3) in Methods, but I have found it hard to grasp its precise meaning. The text above Eqn (3) is also difficult to follow. (Additionally, if one does not refer to the Methods, there is a risk of misunderstanding from the caption of Figure 2. Although there is interannual variability, over a reasonable period, most locations will be in a near-equilibrium state, balancing import and export of moisture). To truly understand the contributions to the "Asymmetry Index," a schematic in the Methods might be helpful.

Response: We appreciate the reviewer's insightful comment and agree that the original description of the Asymmetry Index was not sufficiently clear. In response, we revised the Methods, figure captions, and main text to more explicitly define the index, clarify its interpretation, and reduce the risk of confusion with physical moisture fluxes.

First, we added a paragraph to explain the index in the **Methods** (**Upwind region, source influence, sink sensitivity, and asymmetry index**) as below: "*Source influence and sink sensitivity quantify the asymmetric roles of grid cells in the directional transmission of predictive information related to non-local vegetation effects on SMA. To summarize this directional imbalance, we compute an asymmetry index that compares the relative contributions of information exported downwind ("outgoing") versus information received from upwind ("incoming") for each grid cell, as inferred from predictive attribution in the transport-informed model. The index does not diagnose whether a location exports or imports moisture on average; rather, it indicates whether vegetation at a grid cell exerts a stronger influence on downwind soil moisture than it receives from upwind vegetation. When outgoing contributions dominate, the cell acts as a primary transmitter of predictive information to downwind regions; conversely, when incoming contributions dominate, it is primarily influenced by upwind vegetation (as shown in **Fig. S12**).*" (**lines 510-520**).

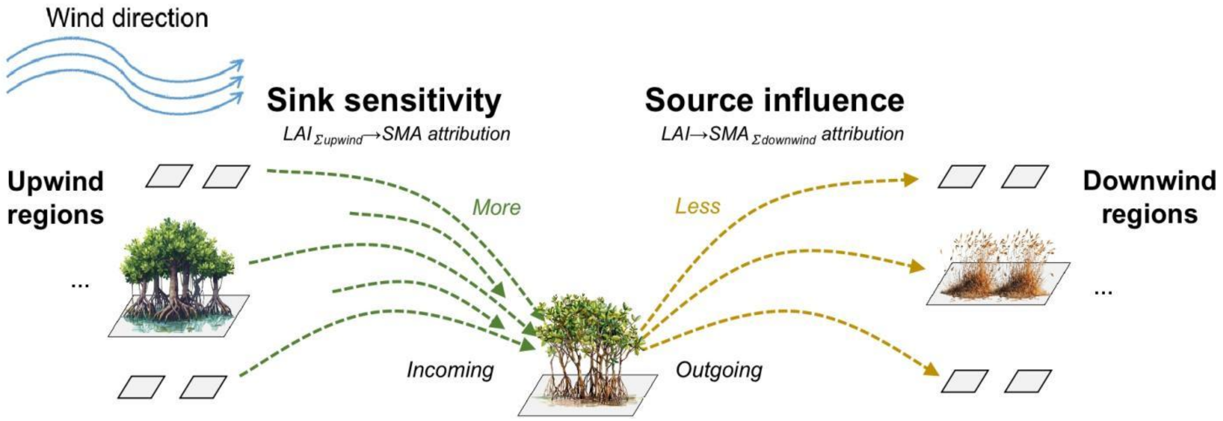

$$Asymmetry\ index = \frac{|Source\ influence| - |Sink\ sensitivity|}{|Source\ influence| + |Sink\ sensitivity|} + \varepsilon$$

Fig. S8 | Schematic illustration of source influence, sink sensitivity, and the asymmetry index.

Second, to address potential confusion with long-term moisture balance, we clarified that "outgoing" and "incoming" refer exclusively to the directional predictive influence inferred from the model, rather than to physical moisture fluxes or long-term hydrological mass balance. The Asymmetry Index is therefore meaningful even in regions where long-term moisture import and export may be approximately balanced, as it captures the directionality of vegetation-mediated influence on soil moisture variability along atmospheric pathways.

Third, we clarified the motivation for introducing the "asymmetry index" at its first occurrence in the section of **Source-sink spatial structure of vegetation-soil moisture teleconnections**: *"The maps show a predominantly positive and coherent source-sink structure, indicating that vegetation-driven influences on soil moisture are not symmetrically distributed. To quantify directional contribution imbalance between information transmitted to downwind regions and*

information received from upwind regions for each grid cell, *we compute an asymmetry index (**Fig. 2c; Methods**; **Fig. S12**). "*(**lines 149-152**).

Finally, we revised the caption of **Fig. 2c** to explicitly state that the index reflects directional predictive influence, as: *" **c**, Asymmetry index (**Methods**; **Fig. S12**), which quantifies whether vegetation at a grid cell exerts a stronger predictive influence on downwind soil moisture ("outgoing") than it receives from upwind vegetation ("incoming") within the transport-informed model. The values range from +1 (predominantly a moisture-exporting source) to 0 (moisture export and import balanced, recycling-dominated), then to -1 (predominantly a moisture-importing sink). "*(**lines 204-207**).

Reviewer 1 (7): The caveats could be elaborated further. I always value "one paper, one message," and this manuscript clearly adopts this approach to identify features of South American soil moisture from 2008 to 2017, emphasising upwind and vegetation dynamics. Such detailed focus enables a better regional understanding compared to, for example, analysing ESM outputs. However, for this analysis to be truly useful, it should be considered within the broader context of climate change and deforestation. Would the authors be willing to extend the caveats or discussion section of the paper to acknowledge this, and offer specific recommendations? For example, future research could compare ESM projections of soil moisture for South America for the contemporary period. Does this reduce the uncertainty bounds for future soil moisture under a changing climate? Could the presented analysis serve as a data point in an emergent constraint across the CMIP6 ensemble? Do large-scale models also demonstrate the dependence on land-use and vegetation dynamics the authors identify in this data-led analysis, thus increasing their reliability for simulations involving significant deforestation?

Response: We thank the reviewer for these thoughtful suggestions and agree that it is important to situate our results within the broader context of climate change, land-use change, and Earth system modeling.

In response, we have expanded the **Discussion** to clarify how our observation-based, transport-informed framework may inform future modeling efforts, which reads: "*Our results have important implications for Earth system models (ESMs), particularly for representing the role of upwind terrestrial processes in shaping soil moisture variability downwind. The substantial performance gain achieved by incorporating transport-informed upwind information (**Fig. 1**) indicates that vegetation and other land-surface factors modulate soil moisture not only locally but also through atmospheric moisture transport, a pathway that is often simplified or inadequately represented in ESMs[58]. In this context, the proposed framework (**Fig. S1**) offers an observation-driven reference for evaluating whether models appropriately capture the sensitivity of soil moisture variability to upwind land-surface conditions under contemporary climate conditions. Moreover, the identified directional asymmetries (**Fig. 2**) and climate-dependent response regimes (**Fig. 3**) provide physically interpretable patterns that may be used to diagnose whether large-scale models reproduce observed non-local land-atmosphere coupling.*" (**lines 323-334**).

At the same time, we explicitly acknowledge the limitations of the analysis and note the future work. As stated in the revised **Discussion**: "*However, our analysis focuses on the contemporary period (2008–2017) and its direct applicability to future climate projections is therefore limited. Future work could compare contemporary ESM simulations of soil moisture over South America with the observationally informed patterns identified here. Such comparisons may help assess model realism and, if combined with independent constraints and longer records, could contribute to emergent-constraint–type applications*[61,62] *across the CMIP6 ensemble.*" (**lines 335-340**).

In line with this framing, we also added to the **Abstract**: "*Our results provide a quantitative basis for representing vegetation-soil moisture teleconnections, with implications for evaluating land-atmosphere coupling in Earth system models,* ..." (**lines 37-38**).

Additional reference:

58 Winckler, J., Lejeune, Q., Reick, C. H., & Pongratz, J. (2019). Nonlocal effects dominate the global mean surface temperature response to the biogeophysical effects of deforestation. Geophysical Research Letters, 46, 745–755.

Cox, P. M. et al. Sensitivity of tropical carbon to climate change constrained by carbon dioxide variability. Nature 494, 341–344 (2013).

Hall, A., Cox, P., Huntingford, C. & Klein, S. Progressing emergent constraints on future climate change. Nature Climate Change 9, 269–278 (2019).

Reviewer 1 (8): In general, the presentation of the diagrams feels really rushed. Boxes are not closed around the panels, there is some ambiguity in places as to which panels the colourbars apply to, the panels are not lined up (they should be on either at least one dimension, horizontal or vertical), the font sizes seem in some places too large, elsewhere too small. If possible, please make the captions as complete as possible, given how people often export figures with captions for use in lectures and talks.

Response: We thank the reviewer for the careful assessment of the figure presentation and agree that several aspects of the figures could be improved in the original submission. In response, we have revised all figures in the manuscript to improve their presentation quality. Specifically, panels are now properly aligned, panel boundaries are clearly delineated, and color bars are properly associated with their respective panels. Font sizes have been standardized across all figures to improve readability. In addition, we expanded all figure captions where relevant to be more self-contained. We really appreciate the reviewer's attention to these presentation details.

Reviewer 1 (9): I am very happy to see this paper again. The overall impression is of an important piece of analysis, full of quite critical insights into the hydrological cycle of South America. However, the current version requires some further work to reach publication standards. I am confident that the authors can accomplish this, and I hope this happens.

Response: We sincerely thank the reviewer for the positive and encouraging assessment of our manuscript. We fully acknowledge that further revisions were required to meet publication standards, and we have carefully addressed all comments. We are grateful for the reviewer's constructive feedback and appreciate the opportunity to revise the manuscript.

Response to Reviewer #2

Reviewer 2 (1): This manuscript presents a well-structured analysis of how upwind vegetation dynamics influence downwind soil moisture across South America using a novel combination of deep learning and moisture-transport information. The study addresses an important scientific question, introduces a creative methodological framework, and provides continent-scale insights. Overall, the work is of high quality and potentially impactful, though several aspects would benefit from clarification before acceptance.

Response: We thank the reviewer for the positive assessment of the manuscript and for the constructive suggestions to improve the clarity and interpretation. We address each point in detail below.

Reviewer 2 (2): The regime classification is based on two criteria: 1) the persistence of the sign of source influence (e.g., >70% positive months), and 2) the shape of the piecewise linear regression between LAI anomaly and source influence. Have the authors tested the other thresholds, like 80% or 90%? Moreover, I suggest briefly summarizing the key steps in the main text, particularly the role of sign persistence and breakpoint-based regression, to improve clarity and readability.

Response: We thank the reviewer for the suggestion regarding robustness and clarity. We have tested alternative persistence thresholds of 60%, 70%, 80%, and 90% (**Fig. S19**). The resulting regime classifications obtained using thresholds between 60% and 80% are generally consistent with those based on the 70% threshold, with the main spatial patterns preserved (e.g., Regime 4, dark blue, and Regime 1, red). This indicates that the regime structure is not sensitive to the specific threshold choice within this range. When applying a 90% persistence threshold, the resulting regime patterns become sparse and are dominated by a small subset of climatically uniform regions. We think this arises because a 90% criterion effectively selects grid cells with near-year-round sign consistency, thereby excluding regions where source influence varies seasonally. Such seasonally varying behavior is a defining feature of the regime framework, particularly in transitional and monsoonal climates where the balance between moisture recycling and ET changes across seasons. We therefore consider the 90% case as overly restrictive for diagnosing spatial regime structure. By contrast, thresholds consistent with the intended regime definition yield spatial patterns that remain coherent and comparable, differ primarily in boundary extent rather than in overall structure.

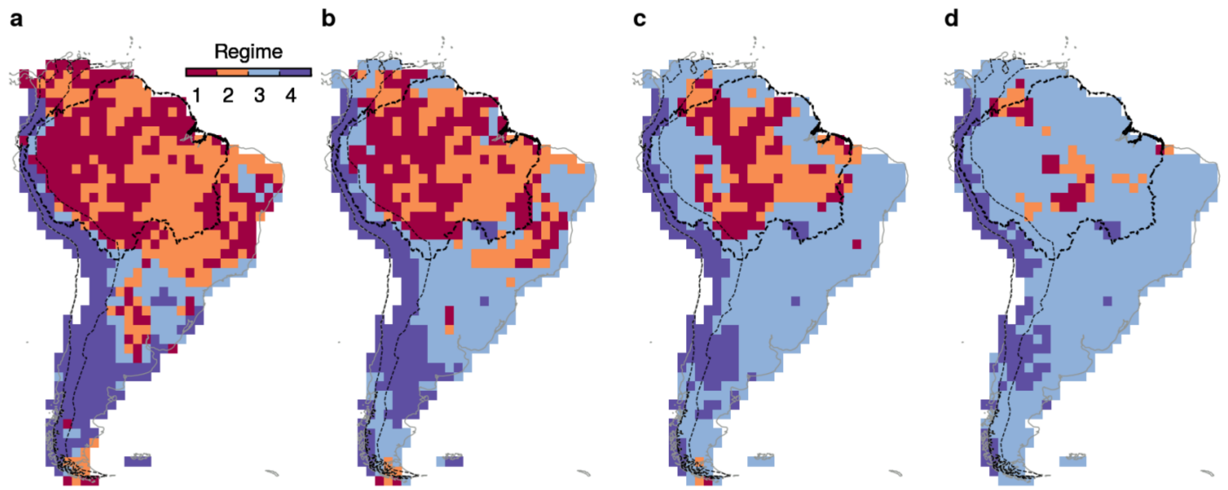

Fig. S19 | Sensitivity of regime classification to persistence thresholds.
*Regimes are classified based on the fraction of months with positive source influence. The figure compares the resulting regime assignments using thresholds of 60% (**a**), 70% (**b**), 80% (**c**), and 90% (**d**).*

To improve clarity and readability, we have also added a brief summary of the regime classification procedure in the main text. In the section on **Climatic regimes of nonlinear vegetation-soil moisture teleconnections**, we added: *"Grid cells are classified into four regimes (R1-R4; **Fig. 3a**) based on the temporal sign persistence of source influence and the post-breakpoint tendency in the relationship between LAI anomalies and source influence (**Methods**). The resulting regime patterns are robust across a range of persistence thresholds within a physically meaningful range (**Fig. S19**)."* (**lines 214-218**) and we revised the caption of Figure 3a accordingly. In the **Methods** (Definition of climatic regimes), we added "***Fig. S19** illustrates that these resulting regime classifications are robust across a range of persistence thresholds centered on the chosen 70% criterion.*" (**lines 551-553**). We also added an additional description in caption of Figure 3 as "***a**, Spatial distribution of four regimes across South America, identified by a 70% temporal sign-persistence threshold of source influence and the post-breakpoint trend in the leaf area index (LAI) anomaly-source influence relationship (**Methods**; see **Fig. S19** for robustness across alternative thresholds).* " (**lines 251-254**).

Reviewer 2 (3): The interpretation of regime 2 and 3 is interesting but underexplained. These two regimes represent the most complex and climatically heterogeneous parts of the continent, so I encourage the authors to provide a clearer mechanistic discussion for these two regimes.

Response: We agreed that Regimes 2 and 3 correspond to climatically heterogeneous regions where vegetation–atmosphere coupling is more complex. In the revised manuscript, we have expanded the **Discussion** to provide a clearer mechanistic interpretation of these regimes, which now reads: "*Vegetation-induced changes in downwind moisture availability can be interpreted as arising from a balance between enhanced ET and local moisture recycling, modulated by surface energy partitioning (e.g., through albedo and aerodynamic roughness effects)* [49, 53]. *When LAI increases from very low values, enhanced ET may be largely retained locally through recycling*

processes, limiting moisture export and yielding weak or negative downwind soil moisture responses[40, 54]*. With continued greening, ET can increasingly exceed local recycling capacity, allowing a larger fraction of moisture to be exported downwind and generating positive responses. Within this conceptual framework, R2 (**Fig. 3c**) may represent environments with intermediate moisture availability, such as the Amazon-Cerrado transition zone, where the transition from recycling-dominated to export-dominated behavior occurs at relatively low positive LAI anomalies. Further increases in LAI can be associated with a stabilization of positive downwind influence, which may reflect partial saturation of ET and increasing competition from local recycling and surface energy constraints. In R3 (**Fig. 3d**), characteristic of drier, more seasonally variable regions may exhibit a different balance. One possible interpretation is that initial greening primarily enhances ET and favors moisture export under a relatively dry boundary layer. As greening continues, however, associated changes in near-surface humidity, boundary-layer structure, precipitation efficiency, and surface roughness could increase the fraction of ET that is locally recycled*[57]*. If such a shift occurs, the amount of ET available for downstream transport would decrease, potentially weakening or even reversing the influence of upwind land conditions on downwind soil moisture.*" (**lines 295-315**).

And accordingly, we provided the new version of Figure 3 to illustrate the ET's dominate role in the linkage of vegetation and soil moisture downwind. Now, the **Climatic regimes of nonlinear vegetation-soil moisture teleconnections** reads: "*..., with peak ET occurring when both LAI anomalies and source influence are high. ... after which source influence weakens (**Fig. 3c**) with a shift also characterized by a relatively high ET state, consistent with increasing water limitation in this transitional zone. ... R3, covering much of the La Plata basin and nearby croplands, exhibits net moisture-importing behavior at the source scale, where initial greening is associated with increased downwind soil moisture, but further greening yields diminishing or negative returns accompanied with insufficient ET (**Fig. 3d**). R4, concentrated in the Andes and Brazilian Highlands, shows weak source influence and a predominantly negative association, consistent with strong local recycling mediated by high-elevation thermal structure and topographic effects*[53-55]*, where high ET percentiles do not trigger significant changes in source influence.*" (**lines 221-241**).

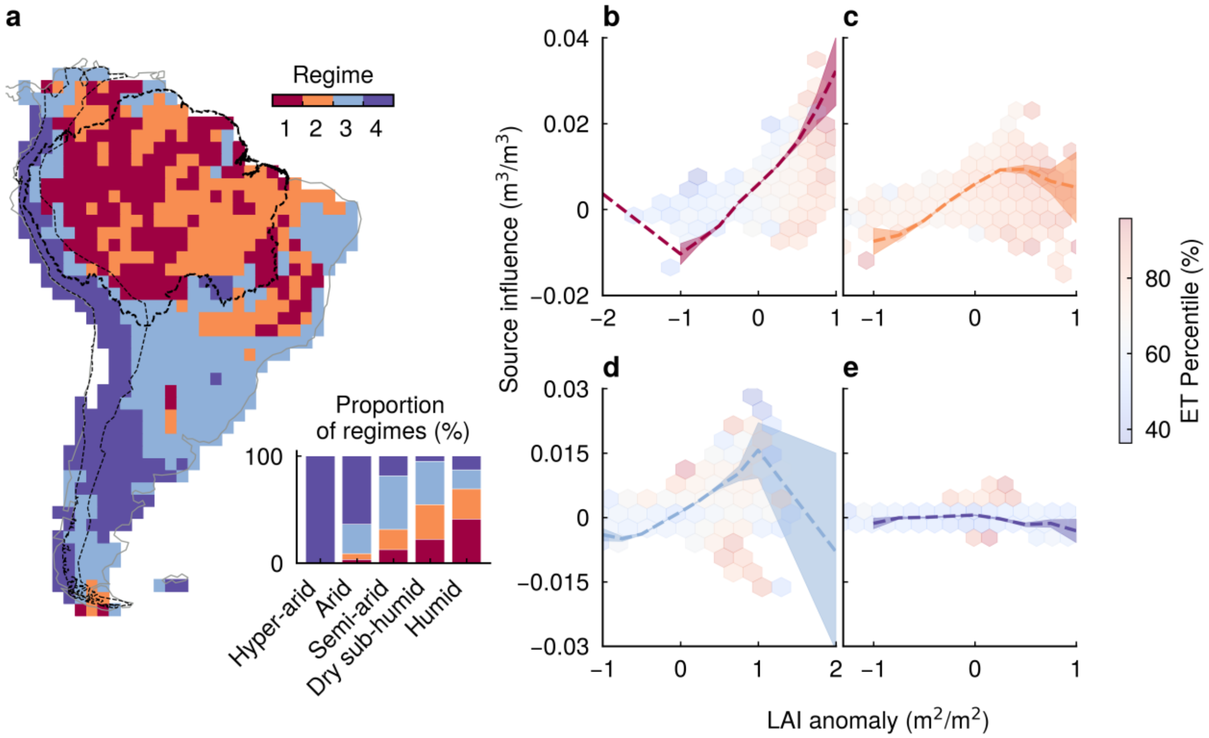

Figure 3* | *Nonlinear regimes of vegetation influence on downwind soil moisture. a, *Spatial distribution of four regimes across South America, identified by a 70% temporal sign-persistence threshold of source influence and the post-breakpoint trend in the leaf area index (LAI) anomaly-source influence relationship (**Methods**; see **Fig. S19** for robustness across alternative thresholds). The inset shows the proportion of each regime within aridity index classes, defined as precipitation (P) to potential evapotranspiration (P/PET: humid, >0.65; dry sub-humid, 0.5-0.65; semi-arid, 0.2-0.5; arid, 0.05-0.2; hyper-arid, <0.05). **b-e**, Mean relationships between LAI anomalies and source influence for each regime (R1-R4; colors match panel **a**), obtained by averaging the piecewise regression curves across grid cells in each regime. Dashed lines show the mean and shaded bands the 95% confidence intervals. Hexagon bins in **b-e** are colored by the evapotranspiration (ET) percentile, calculated based on the distribution of ET values within each individual grid cell.*

Additional reference:

57 Cao, M. et al. Revegetation impacts on moisture recycling and precipitation trends in the Chinese Loess Plateau. Water Resources Research 60, (2024).

Reviewer 2 (4): While the attribution analysis using Expected Gradients provides spatially resolved importance scores, it does not inform the directionality or nonlinear shape of input variables' influence on soil moisture anomaly. For example, how does increasing LAI or temperature affect predicted soil moisture?

Response: We thank the reviewer for this important comment. We agree that Expected Gradients (EG; Erion et al., 2021) quantify the magnitude of predictive attribution, and by themselves, do

not provide a complete functional response curve. In our framework, directionality and nonlinearity are addressed through a combination of attribution-sign analysis and regression-based diagnostics.

Directionality is inferred from the sign of EG values because all dynamic input variables and the SMA output are expressed as standardized anomalies. Under this formulation, positive (negative) attribution values indicate that increases in the input variable are associated with increases (decreases) in predicted SMA relative to the climatological mean. This directional interpretation is illustrated by the relationships between input anomalies and their corresponding EG values (**Fig. S9**) and by spatial maps of mean EG values that exhibit coherent regional attribution patterns (**Fig. S10**).

Nonlinear response shapes are characterized separately using breakpoint-based regression analyses between LAI anomalies and source influence (**Fig. 3**; **Methods**), which capture regime-dependent and non-monotonic behavior. In addition, representative examples demonstrate how the relationship between LAI anomalies and EG attribution differs for upwind, local, and downwind pixels (**Fig. S11**), highlighting spatially dependent nonlinear responses. This example corresponds to the first inset in **Fig. 1c** located in the southern Amazon.

We reference them in **Methods** (**Model evaluation and attribution**): "*While expected gradients measure the magnitude of predictive attribution, the anomaly-based formulation allows attribution signs to be interpreted directionally (**Fig. S9** and **S10**).*" (**lines 486-488**) and in **Source-sink spatial structure of vegetation-soil moisture teleconnections** section: "*... These metrics are derived from predictive attribution combined with regression-based diagnostics that establish directionality and nonlinearity (**Fig. 2a,b**; **Methods**; **Fig. S9-11**).* " (**lines 146-148**).

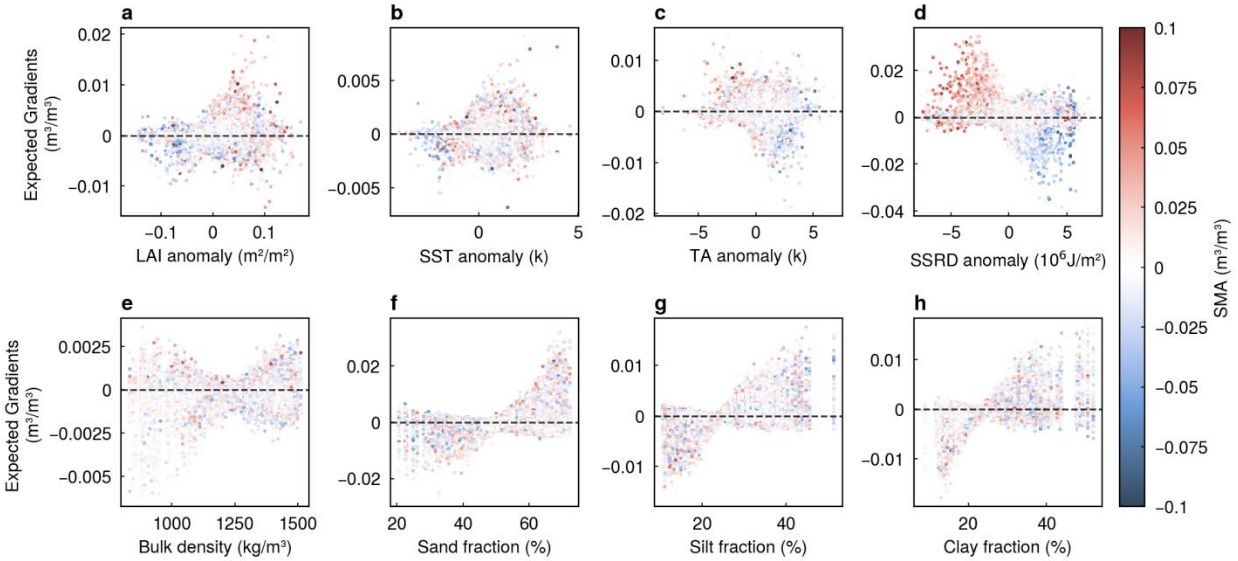

Figure S9 | Directional dependence of predictive attribution (Expected Gradients) on input variables in transport-informed model.

Relationships between inputs (standardized anomalies) and expected gradients (EG) values for individual variables. Because both input variables and soil moisture anomaly (SMA) are expressed as anomalies, the sign of EG provides directional information: positive (negative) attribution

values indicate that positive anomalies of the input variable are associated with increases (decreases) in predicted SMA relative to the climatological mean. The magnitude of EG reflects the local sensitivity of the prediction to perturbations in the input variable. Colors indicate the predicted SMA value. Panels show examples for (a) leaf area index (LAI), (b) sea surface temperature (SST), (c) near-surface air temperature (TA), (d) surface downward shortwave radiation (SSRD), and (f) sand, (g) silt, and (h) clay fractions.

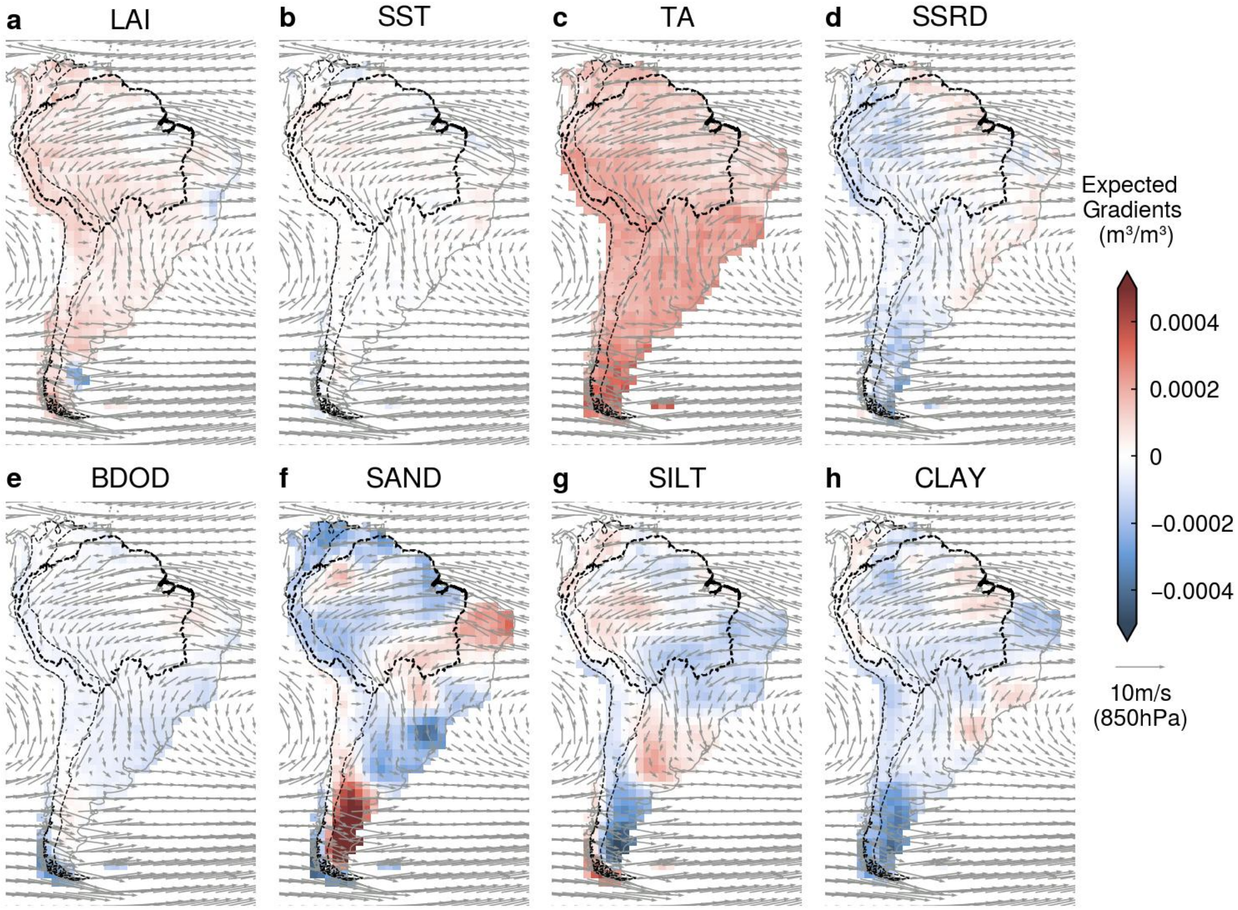

Figure S10 | Spatial distribution of averaged predictive attribution (Expected Gradients) for individual input variables.

Grey arrows indicate climatological 850-hPa wind vectors, shown for reference to atmospheric transport pathways. Panels show results for (a) leaf area index (LAI), (b) sea surface temperature (SST), (c) near-surface air temperature (TA), (d) surface downward shortwave radiation (SSRD), and soil properties including (e) bulk density (BDOD), (f) sand, (g) silt, and (h) clay fractions.

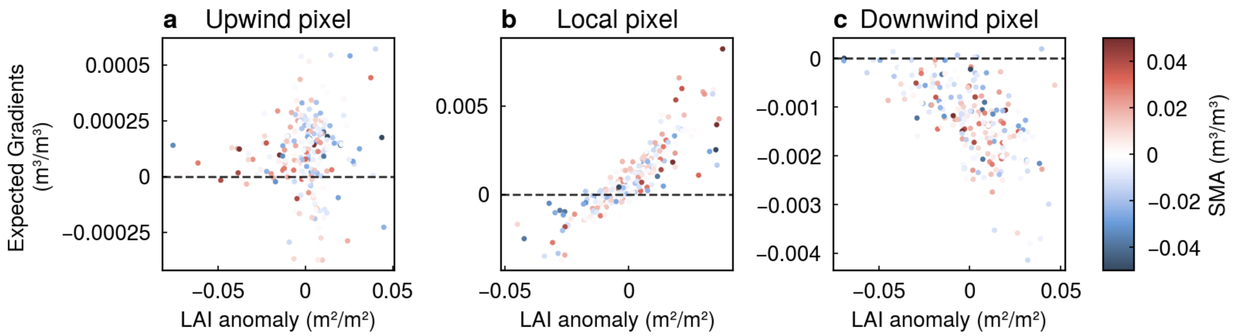

Figure S11 | Example of nonlinear and spatially dependent predictive attribution responses to LAI anomalies.

*Example prediction illustrating the relationship between standardized LAI anomalies and expected gradients (EG) attribution for input pixels located (**a**) upwind, (**b**) local, and (**c**) downwind for a specific target grid cell. This example corresponds to the first inset panel in Fig. 1c of the main text and is located in the southern Amazon. Points are colored by the predicted soil moisture anomaly (SMA).*

Reference:

Erion, G., Janizek, J.D., Sturmfels, P. et al. Improving performance of deep learning models with axiomatic attribution priors and expected gradients. Nature Machine Intelligence 3, 620–631, (2021).

Reviewer 2 (5): The analysis is based solely on surface soil moisture (0–10 cm). However, deeper soil layers (e.g., 10–100 cm) are crucial for plant water uptake, particularly in seasonally dry regions. Could the author discuss whether considering only shallow soil moisture would influence attribution analysis? If deeper soil layers were considered and excluded, please clarify why.

Response: We thank the reviewer for raising this important point. We clarify that throughout this study, soil moisture anomaly (SMA) is treated as the response variable, and the objective is to diagnose how vegetation-related signals from upwind regions are transmitted through atmospheric moisture transport and expressed in downwind land-atmosphere coupling, rather than to characterize vegetation water uptake or plant water stress per se. Within this framework, surface soil moisture (0-10 cm) is an appropriate response variable because it is most directly coupled to ET, precipitation, and boundary-layer processes that mediate atmospheric moisture transport. Root-zone soil moisture, while critical for controlling vegetation state and LAI variability through plant water uptake and physiological regulation, primarily acts as an upstream constraint on vegetation rather than as the receiving variable through which transported atmospheric signals are expressed at the land surface.

In the **Methods** (**Data processing**), we added:

"*Surface soil moisture was selected as the primary variable because it directly couples with ET and boundary layer processes, which is critical for capturing atmospheric moisture transport signals as demonstrated in [Citation].*". (**lines 393-395**)

Nonetheless, we fully agree that incorporating explicit root-zone soil moisture represents an important direction for future work, particularly for assessing vegetation stress and longer-term hydroclimatic impacts. At present, this extension is limited by the availability, consistency, and uncertainty of observation-constrained root-zone soil moisture products at continental scales.

Furthermore, our cross-comparison of multiple soil moisture products (ERA5-Land, GLEAM, GRACE, and SoMo.ml) reveals a significant divergence in anomalies at deeper layers (10-30, 30-50, 50-100 cm) compared to the surface layer (0-10 cm). While surface soil moisture anomalies are relatively well-constrained by satellite remote sensing observations and show high inter-product consensus (Fig. S22), subsurface anomalies exhibit much lower correlations across different datasets. This inconsistency stems from several factors: (1) the high land-surface heterogeneity (e.g., soil texture and vertical root distribution) that governs subsurface dynamics but is difficult to simulate at a 1.5° scale; (2) the lack of direct observational constraints from remote sensing for deeper layers; and (3) the uneven global distribution of in-situ training stations for machine-learning-based products like SoMo.ml. Given these substantial uncertainties, incorporating subsurface soil moisture at this stage could introduce significant noise into the attribution analysis and potentially lead to spurious interpretations of vegetation-mediated signals. We therefore prioritize the use of the 0-10 cm layer as it provides a physically consistent and observation-constrained response variable for diagnosing land–atmosphere coupling.

In the revised **Discussion**, we added: "*While our analysis focuses on surface soil moisture (0-10 cm) to capture rapid land–atmosphere coupling, deeper soil layers play a critical role in sustaining vegetation water uptake and regulating LAI variability through root-zone water availability*[67]*. Root-zone soil moisture typically exhibits longer memory and integrates hydrological processes over seasonal timescales*[27, 68]*. The transport-informed model partly accounts for persistence effects through the temporal structure of SMA anomalies (1-6 months), but does not explicitly represent root-zone dynamics*[69]*. Furthermore, cross-product comparisons (Fig. S22) reveal significant subsurface divergence, likely due to high land-surface heterogeneity and a lack of direct satellite constraints at depth. Incorporating deeper soil moisture may therefore modify the magnitude, persistence, or timing of vegetation-mediated influences inferred here. How such differences would affect the spatial expression and regime structure of vegetation-soil moisture teleconnections remains an open question and represents an important direction for future work, particularly as observation-constrained root-zone soil moisture products with sufficient spatial coverage and uncertainty characterization become available*" (**lines 358-372**).

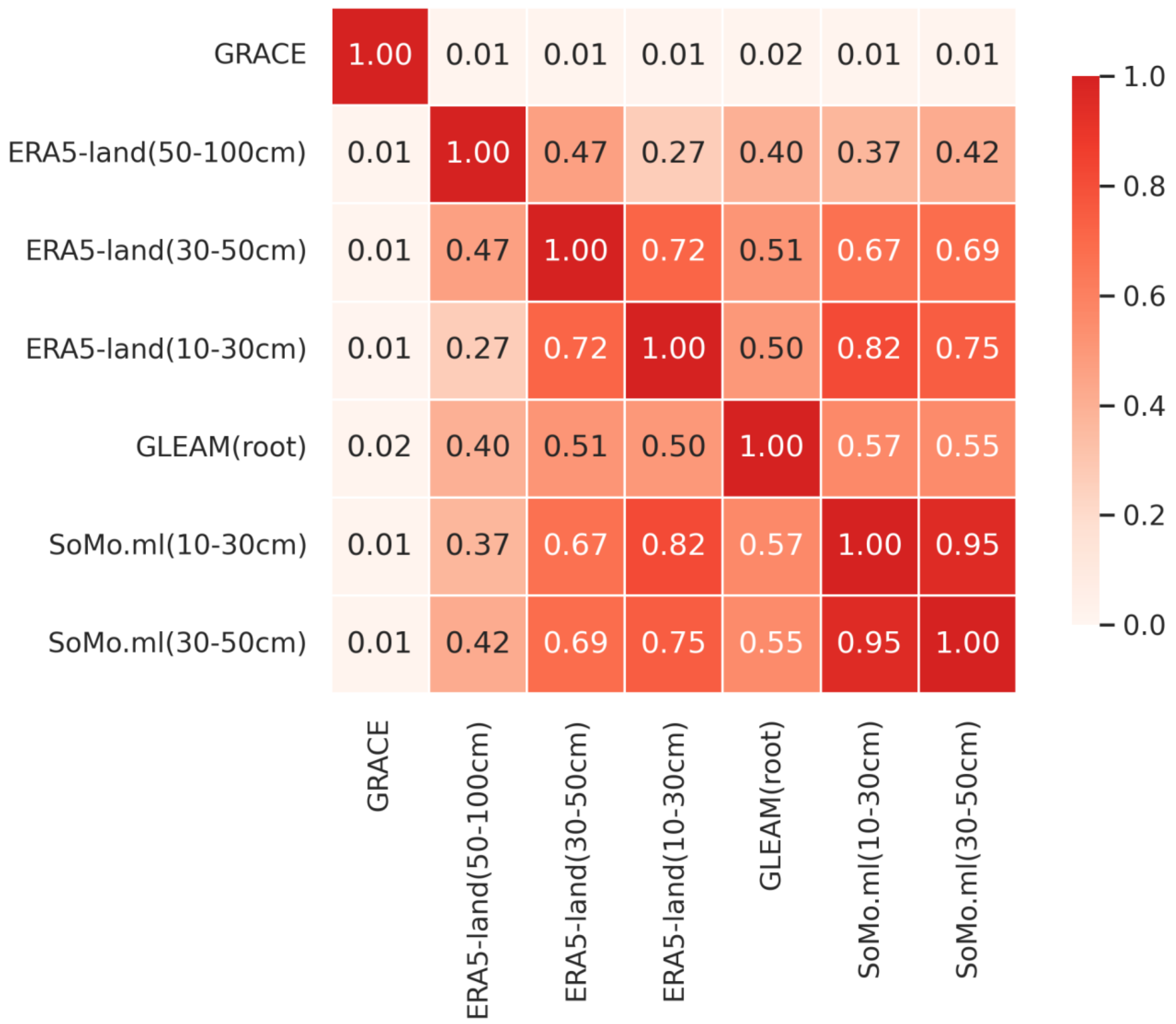

Fig. S22 | Pearson correlation of different sources of subsurface soil moisture anomaly data. *Data are obtained from GRACE (grace.jpl.nasa.gov), ERA5-Land (cds.climate.copernicus.eu), SoMo.ml (nature.com/articles/s41597-021-00964-1), and GLEAM (gleam.eu). For ERA5-Land, subsurface soil moisture data at 10–30 cm, 30–50 cm, and 50–100 cm layers were obtained through linear interpolation of the original levels.*

Additional references:

27 Seneviratne, S. I. et al. Investigating soil moisture–climate interactions in a changing climate: A Review. Earth-Science Reviews 99, 125–161 (2010).

67 Li, W. et al. Widespread increasing vegetation sensitivity to soil moisture. Nature Communications 13, (2022).

68 Rahmati, M. et al. Soil moisture memory: State-of-the-art and the way forward. Reviews of Geophysics 62, (2024).

69 Gao, H. et al. Root zone in the Earth System. Hydrology and Earth System Sciences 28, 4477–4499 (2024).

Response to the Reviewers

Reviewer #1 (1): Thank you for asking me to rereview paper: "Upwind terrestrial influences on soil moisture variability across South America" by Huang et al. First, can I apologise for taking longer than the usual two weeks to return this review. I continue to believe that this manuscript has important implications for agricultural resilience, as measured by changes in soil moisture, calculated downwind of the Amazon rainforest. What I particularly like is that, unlike other papers, the focus is applicable to both potential land-use drivers in the Amazon and climate change ("TA", Figure 1d). I can see that the authors have responded extensively to the review comments, which is appreciated.

Response: We thank the reviewer for these encouraging comments and for recognizing the implications of our work for agricultural resilience and land-use management. We also greatly appreciate the time taken for this thoughtful re-review.

Reviewer #1 (2): My only remaining problem with the paper is that, in places, it is still quite difficult to follow, which is a real shame given its important messages. Getting this right is particularly important for a generalist journal such as Nature Communications. Indeed, even environmental scientists, though very familiar with their own research area, still need to be informed when considering other aspects of large-scale environmental change.

If I can give an example, that may improve the presentation of analysis in Figure 3, and the related text and caption. Anything in the climate system that is nonlinear will always capture the interest of people, so this Figure is important.
The first line of the caption correctly addresses the influence of vegetation on downwind soil moisture. To make this super clear, maybe change the "y"-label to something like "Source influence on downwind soil moisture (m3/m3)."

Response: We fully agree with the reviewer's concern about accessibility. We took this overarching concern seriously.

First, at the manuscript level, we conducted a line-by-line pass to improve clarity throughout, simplifying technical phrasing, adding plain-language bridges, and ensuring consistent, accessible descriptions across the **Abstract**, **Main**, and **Methods** (see our responses to Reviewer #1 (4), (5), and (10)).

Second, for **Figure 3** specifically, as the reviewer noted, we have relabeled the y-axis to be more intuitive, enclosed panels **b–e** in full rectangular boxes for clearer visual definition, and annotated each panel with its corresponding regime name. We also simplified the caption wording accordingly.

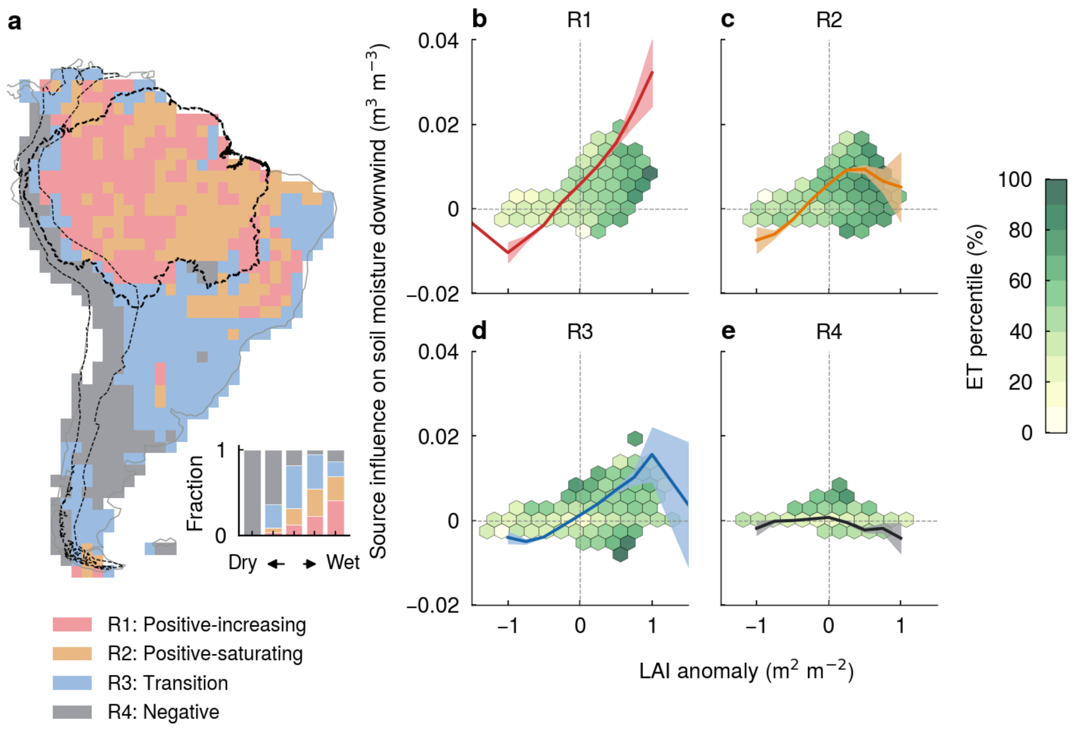

***Figure 3 | Nonlinear regimes of vegetation influence on downwind soil moisture. a**, Spatial distribution of the four climatic regimes across South America, reflecting the consistency and shape of the downwind soil moisture response to upwind vegetation changes. Regimes are defined by whether the source influence remains predominantly positive (>70% of months) and by its post-breakpoint trend (**Methods**; see **Fig. S19** for robustness across thresholds). White areas were excluded from the classification (hyper-arid cells, or cells with no statistically resolved coupling; **Methods**). The inset shows the fractional composition of the four regimes across five aridity classes (displayed from left to right) defined by the precipitation-to-potential evapotranspiration ratio, P/PET: hyper-arid (<0.05), arid (0.05-0.2), semi-arid (0.2-0.5), dry sub-humid (0.5-0.65), and humid (>0.65). **b-e**, Relationship between upwind LAI anomalies (m^2 m^{-2}) and source influence on downwind soil moisture (m^3 m^{-3}) for each regime (R1-R4; color matches panel **a**). Solid lines show the regime-mean source influence within bins of LAI anomaly and shaded bands the 95% confidence intervals; gray dashed lines mark zero. Hexagon bins are colored by the evapotranspiration (ET) percentile (calculated from the distribution of ET values within each grid cell) and require at least five grid cells per bin.*

Third, we have also comprehensively reviewed and revised the captions and descriptions for **Figure 1** and **Figure 2** as follows:

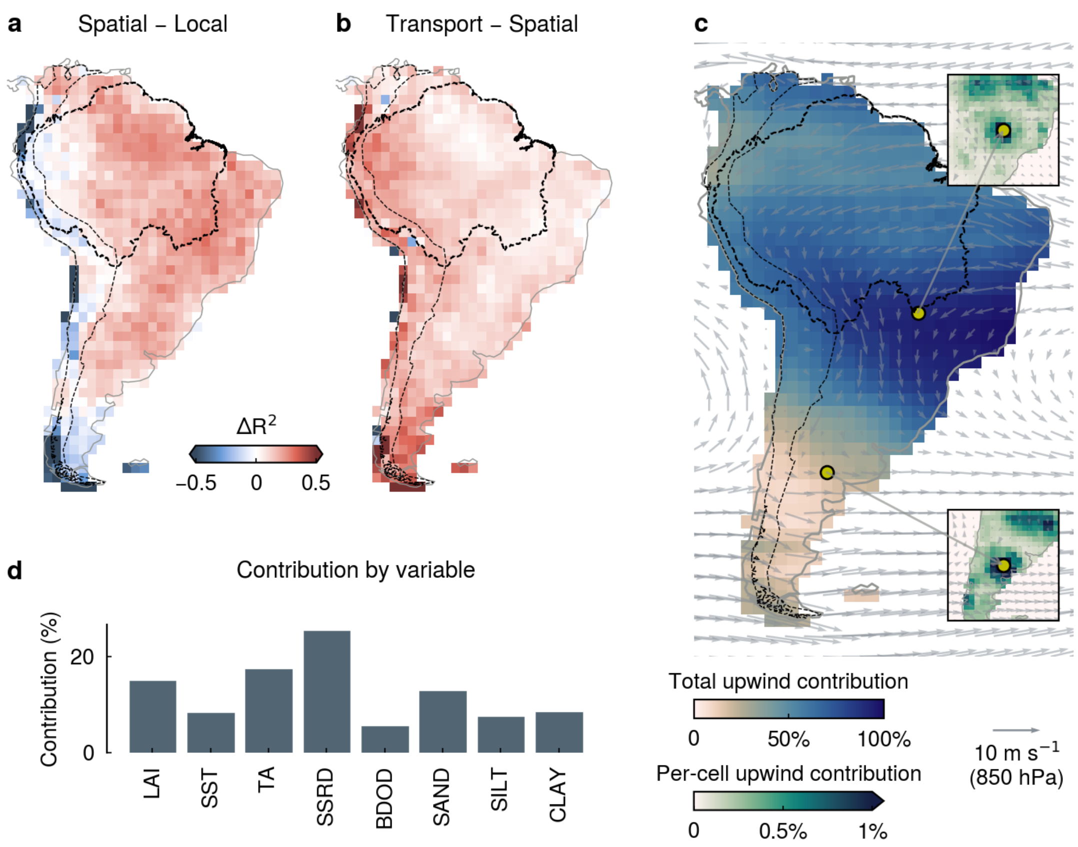

***Figure 1 | Performance gains of the transport-informed model and predictive attribution from land surface and near-surface atmospheric inputs. a**, Spatial-context gain (ΔR^2) from local-only model; positive values (red) indicate that adding non-directional spatial context improves skill. **b**, Transport-informed gain (ΔR^2) from spatial-context model; positive values indicate the additional gain from transport-informed directionality. Panels **a** and **b** share the ΔR^2 color scale. **c**, Total upwind contribution: for each target grid cell, the fraction of predictive attribution originating from all upwind terrestrial grid cells, computed as the summed absolute attribution over upwind cells normalized by the summed absolute attribution over all input grid cells in its 21×21 window (a value of 100% indicates complete dependence on upwind information). Upwind cells are identified with UTrack[34] (**Methods**); predictive attribution is from expected gradients[38] (**Methods**). The two insets show, for two representative target cells (yellow points), how this attribution is distributed across the surrounding source cells; colors give the per-cell contribution (% of that target cell's total attribution), so the value mapped in c equals the sum over the upwind cells shown in each inset. Gray arrows are climatological 850 hPa wind vectors from ERA5 (2001-2018). **d**, Contribution by variable (%), showing the share of total upwind predictability assigned to each terrestrial variable (summing to 100% across all eight variables) in the transport-informed model. It is estimated by the fraction of total absolute attribution over all upwind grid cells assigned to each variable. Variables include leaf area index (LAI), sea surface temperature (SST), air temperature at 2 m (TA), surface downward shortwave radiation (SSRD), bulk density (BDOD), and sand (SAND), silt (SILT), and clay (CLAY) fractions.*

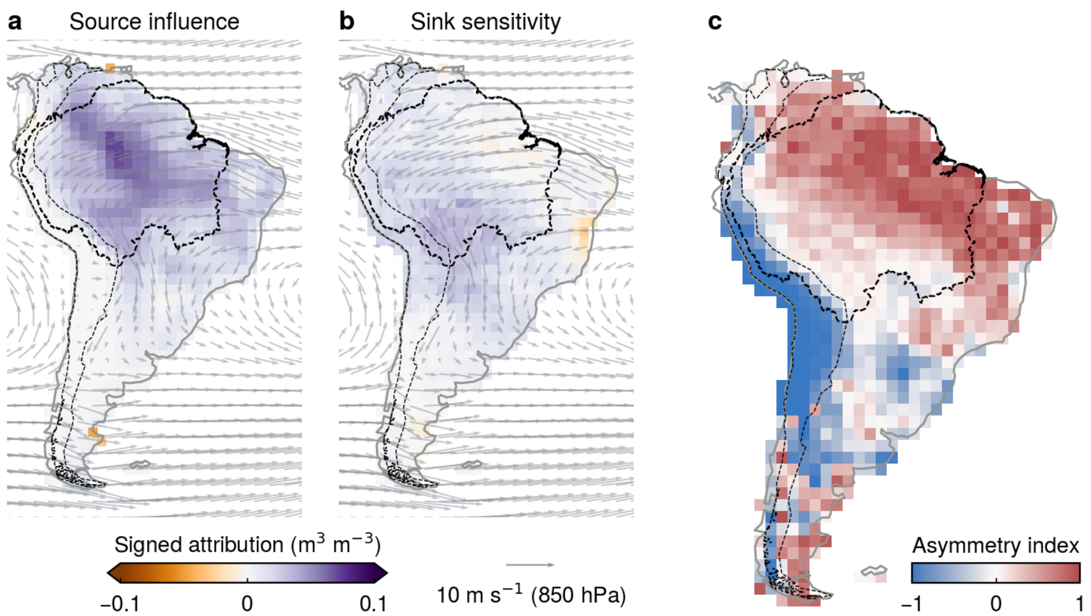

Figure 2 | Spatial structure of directional vegetation–soil moisture teleconnections across South America. ***a****, Source influence, which quantifies the outgoing effect of local leaf area index (LAI) anomalies on downwind soil moisture anomalies (SMA), averaged over its downwind target cells (2001-2018;* ***Methods****).* ***b****, Sink sensitivity, which quantifies the incoming effect of upwind LAI anomalies on local SMA, averaged over all upwind source cells (2001-2018;* ***Methods****). Positive values (purple) indicate that greening is associated with wetter downwind soil; negative values (orange) indicate the opposite. Panels* ***a*** *and* ***b*** *share the same color scale.* ***c****, Asymmetry index (****Methods****;* ***Fig. S12****), computed from the magnitudes |source influence| and |sink sensitivity|. Values range from +1 (predominantly source/outgoing) through 0 (balanced) to -1 (predominantly sink/incoming), which reflects whether vegetation at a cell exerts a stronger influence on downwind soil moisture than it receives from upwind. Gray arrows in* ***a-c*** *indicate climatological 850 hPa wind vectors from ERA5 (2001-2018).*

We hope these changes, taken together, make the manuscript substantially easier to follow for a broad readership.

Reviewer #1 (3): Land-use change is likely to cause LAI decreases, and so this is why this diagram needs prominence. Please ensure that specific point is reiterated more strongly in the Discussion.

Response: We thank the reviewer for this constructive insight, and agree the causal chain “land-use change -> LAI decline -> reduced downwind moisture supply” is important to the practical relevance of our study and is precisely what makes **Figure 3**

important. To reiterate this point more strongly, we now state it explicitly and prominently early in the **Discussion** and tie it directly to the regimes in **Figure 3**:

1) "*This asymmetry (**Fig. 2c**) indicates a critical vulnerability. Anthropogenic land-use change, which widely triggers severe LAI declines through deforestation and land degradation, especially in humid regions (R1 and R2 in **Fig. 3a**), threatens to disrupt these moisture supply regimes. Particularly if land-use change-driven forest loss exceeds ~40% in transitional zones[56], the associated persistent drop in LAI could shift moisture exporting regions toward weaker-export or recycling-dominated regimes. Such a shift would weaken atmospheric moisture supply lines that currently buffer agricultural zones against drought[48, 50].* " (**lines 294-300**).

2) "*Crucially, this framework operates bi-directionally: while vegetation greening alters transport fractions, land-use change-induced LAI reduction reverses these pathways with distinct non-linear thresholds (as prominently illustrated in **Fig. 3**).*" (**lines 306-308**).

3) "*Within this conceptual framework, the non-local land-atmosphere coupling and its non-linear behavioral shifts vary systematically across the identified hydroclimatic gradients. In the humid tropical core in the R1 zone (**Fig. 3a**), the system exhibits a non-linear response that is highly sensitive to canopy degradation (**Fig. 3b**). While positive LAI anomalies show an increasing effect because background moisture is perennially abundant and the system is limited by radiation, negative LAI anomalies follow a different trajectory. Specifically, lowering LAI, whether driven by anthropogenic deforestation or climate-driven dieback, leads to a direct reduction in moisture export along the dominant atmospheric transport pathways. This non-linear sensitivity underscores that canopy loss in the humid core can substantially alter soil moisture downwind, directly reducing the remote moisture supply during critical periods.*

*Moving away from the humid core region, the response pattern observed in the R2 regime (**Fig. 3c**) is consistent with environments of intermediate moisture availability, such as the Amazon-Cerrado transition zone. In these regions, the transition from recycling-dominated to export-dominated behavior appears to occur at relatively small positive LAI anomalies. Once this transition is reached, further increases in LAI are associated with a stabilization of positive source influence, which may reflect increasing constraints from local recycling and surface energy partitioning. Conversely, within the context of intense land-use change, even minor LAI decreases in R2 (**Fig. 3c**), characteristic of the Amazon-Cerrado transition zone, can abruptly shift the system back into a recycling-dominated state, effectively shutting down downwind moisture relief during critical periods. The R3 regime (**Fig. 3d**), characteristic of drier and more seasonally dynamic climates, suggests a different balance between these processes. Initial greening may enhance ET under relatively dry atmospheric conditions, favoring atmospheric moisture export and leading to increasing source influence. However, continued increases in vegetation density may*

lead to the amount of ET available for atmospheric transport to decline, potentially weakening or even reversing the influence of upwind land conditions on downwind soil moisture, through associated changes in near-surface humidity, boundary-layer structure, precipitation efficiency, surface roughness, and even local soil moisture deficit[57]*. In the driest highland environments (R4,* ***Fig. 3e****), source influence is weak and predominantly negative, with local recycling dominating and upwind vegetation exerting little net downwind effect.*" (**lines 317-347**).

Reviewer #1 (4): The methodological part is tough to follow. For instance, I really wanted to understand the threshold for the four nonlinear types. The caption is quite technical? But in particular, I wanted to understand what "identified by a 70% temporal sign-persistance threshold….and post-breakpoint trend" actually means in a more general sense.

Response: We sincerely thank the reviewer for this constructive feedback. We recognize that the previous description was overly technical and lacked a clear bridge between the technical metrics and their physical implications.

1) To address this, we substantially revised the "**Definition of climatic regimes**" subsection in the **Methods** section to make it accessible for a generalist audience. In particular, we now explain in plain terms that the **70% sign-persistence threshold** separates locations where greening reliably boosts downwind moisture from those where the effect is inconsistent, and that the **post-breakpoint trend** distinguishes couplings that keep strengthening (R1) from those that saturate (R2). The revised subsection now reads:

"**Definition of climatic regimes**

We classify each land grid cell into one of four regimes that summarize how upwind vegetation couples to downwind soil moisture. The classification proceeds in two stages: we first stratify cells by the temporal sign persistence of the source influence, and then, within the persistently positive cells, by the shape of the non-linear response to LAI anomaly. Here, the source influence is evaluated monthly. Before classification, we exclude hyper-arid cells and cells whose source influence is not statistically distinguishable from zero coupling, so that the regimes describe robust and active land–atmosphere interactions. The remaining cells are then classified as follows.
In stage 1, we quantify sign persistence as the percentage of months in which the monthly source influence is positive, and test it against a null hypothesis of random sign occurrence using a Binomial test (the probability of a positive month = 0.5, α = 0.05). This splits cells into three bands: persistently positive (>70% of months), transitional (30–70%), and persistently negative (<30%). A high persistence (>70%) identifies a stable climatologically consistent positive co-variation between upwind LAI and downwind moisture.
In stage 2, for persistently positive cells, we further characterize the coupling by fitting a single-breakpoint piecewise linear model to monthly source influence (y) versus LAI

*anomaly (x) at each cell (**Eq. 6**). The breakpoint is a freely floating variable optimized for each grid cell by minimizing the residual sum of squares using a genetic algorithm. Hence, what this means is that the model independently determines the specific vegetation threshold at which the coupling intensity shifts for every location. Both pre- and post-breakpoint slopes (k_1, k_2) are required to be significant (two-tailed t-tests, $p < 0.05$); the sign of ($k_2 - k_1$) then separates a strengthening from a saturating coupling.*

$$y = \begin{cases} k_1 x + b_1, x < breakpoint \\ k_2 x + b_2, x \geq breakpoint \end{cases} \quad (6)$$

where k_1 and k_2 are the slopes before and after the breakpoint respectively, and b_1 and b_2 are intercepts.

*The four regimes (**Fig. 3b–e**) follow directly:*

- *Regime 1: Positive-increasing (**Fig. 3b**), identified by >70% positive months with a steepening slope ($k_1 < k_2$); coupling strengthens as LAI anomaly increases.*
- *Regime 2: Positive-saturating (**Fig. 3c**), identified by >70% positive months with a flattening slope after the breakpoint ($k_1 > k_2$); the positive influence plateaus beyond a certain LAI anomaly.*
- *Regime 3: Transition (**Fig. 3d**), identified by 30–70% positive months; the vegetation-soil moisture link is inconsistent or frequently switches sign.*
- *Regime 4: Negative (**Fig. 3e**), identified by <30% positive months; LAI anomaly and downwind moisture supply are predominantly inversely associated.*

*Every non-masked cell falls into one of these four regimes. **Fig. S19** confirms that the classification is robust to alternative persistence thresholds around the 70% criterion.*" **(lines 583-620**).

We have also rigorously refined and polished other related subsections in the **Methods** to meet the high standards of publication. These include "**Model structures**", "**Model evaluation and attribution**", and "**Upwind region, source influence, sink sensitivity, and asymmetry index**".

2) In **Model structures,** we rephrased the description of three models: "*We develop three predictive deep learning models to estimate SMA, each incrementally adding spatial and physical context to isolate the effect of atmosphere transport information.*" (**lines 464-465**), and "*The local-only model serves as the baseline model, relating only local predictors at the target grid cell to local SMA via one-dimensional convolutional layers and fully connected layers*" (**lines 471-473**), "*The spatial-context model expands this view by incorporating non-directional surrounding context from a 21×21 grid-cell window (31.5°×31.5°) centered on each target cell.*" (**lines 474-475**), and "*The transport-informed model introduces a physically guided attention mechanism to capture the directionality of atmospheric moisture transport (**Fig. S1**).*" (lines **479-480**).

3) In **Model evaluation and attribution**, we reorganized it into "**Model evaluation**",

"**Predictive attribution**", and "**Quantifying upwind dominance**" subsections. Now it reads:

"*Model performance evaluation*

*Model performance is evaluated using the coefficient of determination (R^2) computed on held-out folds. Reported values are averaged across the four folds. To capture different dimensions of model skill, we compute: (1) An overall R^2 by concatenating test-set predictions across all grid cells and months; (2) A per-grid-cell temporal R^2 to map spatial patterns. Differences between models are expressed as ΔR^2 relative to the local-only and spatial-context baselines (**Fig. 1**); (3) To examine seasonal structure, we average per-grid-cell R^2 over space for each calendar month, producing the monthly skill shown in **Fig. S2**. For context, **Fig. S3** shows the spatial mean and standard deviation of LAI anomalies for each month.*

Predictive attribution

*To determine how much each terrestrial input contributes to the model's predictions, we estimate predictive attribution in the transport-informed model using expected gradients[38] and verify patterns with GradientSHAP[76]. Expected gradients integrates gradients from a reference distribution to produce attribution scores for every variable-location pair (see **Supplementary Information**). Attributions are rescaled to physical units (m^3 m^{-3}) by inverting the min-max normalization of the output. To identify long-term drivers on prediction, we summarize contributions by averaging absolute attributions over time. Shares are computed as the fraction of total absolute attribution assigned to a given variable or region (**Fig. 1c** and **d**). The anomaly-based formulation allows attribution signs to be interpreted directionally (**Figs. S9** and **S10**).*

Quantifying upwind dominance

*To assess the dominance of upwind processes, we calculate at each target cell and month the ratio between the absolute attribution summed over upwind land grid cells and the absolute attribution summed over all grid cells in the model's 21×21 input window. Upwind cells are those whose UTrack fractional moisture contribution to the target cell exceeds 0.1%. The denominator includes the local cell, and ocean cells are excluded throughout. We then average this ratio over time and across land cells to produce the maps in **Fig. 1c**. Variable-specific contributions (for example, LAI) are calculated the same way but restrict the numerator and denominator to the variable of interest; all results use absolute attributions (**Fig. 1d**).*" (**lines 501-530**)

4) In "**Upwind region, source influence, sink sensitivity, and asymmetry index**", we added mathematical definition of source influence and sink sensitivity. Now, it reads:

"*…We define two metrics based on the LAI anomalies predictive attributions (expected gradients) from the transport-informed model. For each month, the attribution from a*

cell's LAI anomalies to a target cell's SMA is signed, so that its sign indicates the direction of the ecological effect (positive: greening associated with wetter downwind soil; negative: with drier downwind soil). Source influence quantifies the outgoing effect of a given cell. For any source cell i, it is computed as the signed attribution from its LAI anomaly to downwind soil moisture (j), averaged across all downwind target cells ($N_{downwind}$) within its 21×21 neighborhood:

$$Source\ Influence(i) = \frac{1}{N_{downwind}} \sum_{j \in downwind} Attribution(LAI\ anomaly_i \rightarrow SMA_j) \quad (3)$$

Sink sensitivity quantifies the incoming sensitivity of a given cell. For a given target grid cell j, is the signed attribution from upwind cells' LAI anomalies to that target's SMA, averaged over all upwind source cells (N_{upwind}) in the same neighborhood.

$$Sink\ sensitivity(j) = \frac{1}{N_{upwind}} \sum_{i \in upwind} Attribution(LAI\ anomaly_i \rightarrow SMA_j) \quad (4)$$

*Retaining the sign is what allows the directional interpretation used in **Fig. 2** and the sign-persistence classification of regimes in **Fig. 3**. For the **Fig. 2a and b** maps, both the source influence and sink sensitivity are averaged over time to give a single value per cell; for the regime classification in **Fig. 3**, we instead use the monthly source influence and quantify how often it is positive (see "**Definition of climatic regimes**"). Source influence and sink sensitivity quantify the asymmetric roles of grid cells in the directional transmission of predictive information related to non-local vegetation effects on SMA. To summarize this directional imbalance, we compute an asymmetry index from the magnitudes of the two time-mean metrics, so that it compares the strength of the influence a cell exerts downwind ("outgoing") with the influence it receives from upwind ("incoming"):*

$$Asymmetry\ Index = \frac{|Source\ Influence| - |Sink\ Sensitivity|}{|Source\ Influence| + |Sink\ Sensitivity| + \epsilon} \quad (5)$$

*with $\epsilon = 10^{-5}$ for numerical stability. The index ranges from -1 to +1, where values approaching +1 indicate a predominantly outgoing (source-dominated) cell, values near 0 indicate balance, and values near -1 indicate a predominantly incoming (sink-dominated) cell. The index does not diagnose whether a location exports or imports moisture on average; rather, it indicates whether vegetation at a grid cell exerts a stronger influence on downwind soil moisture than it receives from upwind vegetation. When outgoing contributions dominate, the cell acts as a primary transmitter of predictive information to downwind regions; conversely, when incoming contributions dominate, it is primarily influenced by upwind vegetation (as shown in **Fig. S12**).*" (lines **532-569**)

We believe these revisions have significantly enhanced the clarity and readability of our methodological framework.

Reviewer #1 (5): To achieve (4), the reader is sent, correctly to "Methods". In the methods, the key part is "Definition of climatic regimes". Somehow the description there feels slightly incomplete. How is the binomial test applied, e.g. is the breakpoint a freely floating variable? The four regimes are described as "positive-saturating",

“positive-saturating”, “transition” and “negative”. However, does this order of the cases relate to the order of panels b, c, d and e? If so, then state this, or annotate the panels. For instance, is panel 3e actually “negative”. Maybe it is negative, but the diagram shows little change? The paper is very technical, and it is important that all information is made available. However, it also remains important that everything is written in an accessible way. For each technical point, extra words nearby something like “Hence, what this means is that….” all help.

Response: We thank the reviewer for these detailed suggestions, which have helped us improve the clarity of the **Methods**. We address each point below.

1) We now explicitly state that the breakpoint is a freely floating variable, and we specify how the Binomial test is applied:

“*In stage 1, we quantify sign persistence as the percentage of months in which the monthly source influence is positive, and test it against a null hypothesis of random sign occurrence using a Binomial test (the probability of a positive month = 0.5, α = 0.05)*” (lines **592-594**), and “*The breakpoint is a freely floating variable optimized for each grid cell by minimizing the residual sum of squares using a genetic algorithm. Hence, what this means is that the model independently determines the specific vegetation threshold at which the coupling intensity shifts for every location*.” (lines 600-603).

2) Yes, the order of the four regimes corresponds directly to panels **b**–**e**. We now state this correspondence explicitly in the text, and, following the reviewer’s suggestion, annotate each panel with its regime name (**Fig. 3**). We also corrected the earlier duplicate naming, where two regimes had both been labelled “positive-saturating”. The revised definitions read:

“*Regime 1: Positive-increasing (**Fig. 3b**), identified by >70% positive months with a steepening slope ($k_1 < k_2$); coupling strengthens as LAI anomaly increases.*
*Regime 2: Positive-saturating (**Fig. 3c**), identified by >70% positive months with a flattening slope after the breakpoint ($k_1 > k_2$); the positive influence plateaus beyond a certain LAI anomaly.*
*Regime 3: Transition (**Fig. 3d**), identified by 30–70% positive months; the vegetation-soil moisture link is inconsistent or frequently switches sign.*
*Regime 4: Negative (**Fig. 3e**), identified by <30% positive months; LAI anomaly and downwind moisture supply are predominantly inversely associated.*” (lines **610-618**).

3) To address the reviewer's concern about the visibility of the trend in Panel 3e, we kept panels b–e on a common axis for direct comparability and clarify that the 'Negative' label is assigned by the sign-persistence criterion (source influence positive in <30% of months), not by the position of the regime-averaged curve. At the level of individual cells and months, the LAI–moisture association in this regime is predominantly negative; the regime-averaged curve appears muted because averaging

across heterogeneous cells dampens the signal. This is fully consistent with the sign-persistence definition.

4) Following the reviewer's suggestion, we added interpretive, plain-language sentences throughout the "**Definition of climatic regimes**" subsection to keep each technical step accessible to a generalist audience (see our response to Reviewer #1 (4)).

Reviewer #1 (6): I'm assuming that every location falls into at least one of the four panels? Correct? So there is no "no change" option for ET as a function of LAI. Hence, even small negative gradients would fall in the "negative" case? (But if there is no "no change" option, how does that fit with the statistical thresholds of change set out in Methods?)

Response: We thank the reviewer for this precise question, which concerns both the completeness of the classification and how it relates to the statistical thresholds in the **Methods**. We clarify both aspects.

1) Not every location is classified. Before classification, we mask out hyper-arid cells (e.g., the Atacama desert) and cells whose source influence is statistically indistinguishable from no coupling. These appear as white (blank) areas in **Figure 3a** and are not assigned to any regime.

2) Within the classified domain, "no change" cells are not forced into the "Negative" regime. The regime label is set by the sign-persistence of the source influence, which is itself significance-tested (Binomial test, $\alpha = 0.05$). A cell is labelled "Negative" (R4) only when its source influence is positive in significantly fewer than 30% of months—that is, when the negative sign is statistically consistent, not merely a small or incidental negative gradient. Cells whose positive fraction is statistically indistinguishable from random (≈50%, the "no change" case the reviewer describes) fall in the Transition regime (R3, 30–70%) or, where the overall signal is negligible, are removed by the mask. Regime assignment additionally requires the piecewise slopes (k_1, k_2) to be statistically significant ($p < 0.05$); cells failing this are not classified. The statistical thresholds in the Methods are therefore precisely what prevent weak or "no change" cells from being mislabelled as "Negative".

3) To make this explicit for readers, we note in both the **Figure 3 caption** and the **Methods** that masked cells appear as white areas and are excluded:

In the **Figure 3a caption**: "*White areas were excluded from the classification (hyper-arid cells, or cells with no statistically resolved coupling;* ***Methods****).*" (lines **254-255**). In the **Methods** section: "*Before classification, we exclude hyper-arid cells and cells whose source influence is not statistically distinguishable from zero coupling, so that the regimes describe robust and active land-atmosphere interactions.*" (lines **588-590**).

Reviewer #1 (7): The presentation of Figure 3 can be improved a little. Close the rectangular boxes of b-e, add some space around each. The background colours used, along with the colourbar, appear especially weak – although that could be my printer (I have a paper copy of the manuscript in front of me).

Response: We thank the reviewer for these suggestions, which we have implemented in the revised **Figure 3**. We hope the revised figure now reads clearly regardless of the output device.

Reviewer #1 (8): Regarding the extra information of ET Percentile, the caption description is "ET percentile,… based on distribution of ET values within each individual grid cell". I think I understand this, but then why does the colourbar not stretch from 0% to 100%, if every location is represented in one of panels b-e? Apologies if I am missing something very obvious.

Response: We thank the reviewer and apologize for this oversight in the previous figure. The reviewer is correct that, since the ET percentile is calculated locally for each individual grid cell, the values collected across all cells within a regime should span the full range from 0-100% range. The earlier truncation was caused by an error in the hexbin aggregation and colorbar normalization. We have now corrected this in the revised **Figure 3**, so that the colourbar now spans the full 0-100% range and the hexbin values are distributed across it, consistent with the methodological description and the underlying data.

Reviewer #1 (9): Figure 3 is important because in the humid regions, many locations are "red" (Figure 3a; regime "1"), and so Figure 3b shows that lowering LAI (e.g. deforestation or climate-driven "dieback") can substantially alter soil moisture downwind. Please make sure the Discussion/Conclusions are incredibly clear and simple in the way they cite back to Figure 3.

Response: We thank the reviewer for this suggestion. To make the link to **Figure 3** as clear and simple as possible, the **Discussion** now opens its synthesis with a single sentence that maps each regime behaviour directly to its panel:

"*Humid rainforests robustly support downwind moisture export (**Fig. 3b**), whereas teleconnection strength saturates (**Fig. 3c**) and can reverse in transitional or semi-arid regions (**Fig. 3d**), where local recycling can dominate over downwind transport*." (lines **291-294**).

Building on this, we reinforce the specific warning the reviewer highlights: in the humid region (Regime 1, the widespread red cells in **Fig. 3a**), lowering LAI through deforestation or climate-driven dieback directly reduces downwind moisture supply (**Fig. 3b**). The land-use vulnerability that follows is developed in the subsequent paragraph (quoted in our response to Reviewer #1 (3)), so we do not repeat it here.

We then explain the behaviour regime by regime, with each step tied explicitly to its panel

4) "*Within this conceptual framework, the non-local land-atmosphere coupling and its non-linear behavioral shifts vary systematically across the identified hydroclimatic gradients. In the humid tropical core in the R1 zone (**Fig. 3a**), the system exhibits a non-linear response that is highly sensitive to canopy degradation (**Fig. 3b**). While positive LAI anomalies show an increasing effect because background moisture is perennially abundant and the system is limited by radiation, negative LAI anomalies follow a different trajectory. Specifically, lowering LAI, whether driven by anthropogenic deforestation or climate-driven dieback, leads to a direct reduction in moisture export along the dominant atmospheric transport pathways. This non-linear sensitivity underscores that canopy loss in the humid core can substantially alter soil moisture downwind, directly reducing the remote moisture supply during critical periods.*

*Moving away from the humid core region, the response pattern observed in the R2 regime (**Fig. 3c**) is consistent with environments of intermediate moisture availability, such as the Amazon-Cerrado transition zone. In these regions, the transition from recycling-dominated to export-dominated behavior appears to occur at relatively small positive LAI anomalies. Once this transition is reached, further increases in LAI are associated with a stabilization of positive source influence, which may reflect increasing constraints from local recycling and surface energy partitioning. Conversely, within the context of intense land-use change, even minor LAI decreases in R2 (**Fig. 3c**), characteristic of the Amazon-Cerrado transition zone, can abruptly shift the system back into a recycling-dominated state, effectively shutting down downwind moisture relief during critical periods. The R3 regime (**Fig. 3d**), characteristic of drier and more seasonally dynamic climates, suggests a different balance between these processes. Initial greening may enhance ET under relatively dry atmospheric conditions, favoring atmospheric moisture export and leading to increasing source influence. However, continued increases in vegetation density may lead to the amount of ET available for atmospheric transport to decline, potentially weakening or even reversing the influence of upwind land conditions on downwind soil moisture, through associated changes in near-surface humidity, boundary-layer structure, precipitation efficiency, surface roughness, and even local soil moisture deficit*[57]*. In the driest highland environments (R4, **Fig. 3e**), source influence is weak and predominantly negative, with local recycling dominating and upwind vegetation exerting little net downwind effect.*" (**lines 317-347**) to present the risk of land-use change on soil moisture downwind.

Reviewer #1 (10): In summary, the paper is near, and its findings are especially important – I can imagine this manuscript appearing in Nature Communications. But could the authors please make a final sweep through, checking that everything is as clear as possible to readers? Although the comments above are directed to Figure 3, please check the manuscript elsewhere for similar issues.

Response: We are grateful for the reviewer's positive assessment. Following this advice, we conducted a line-by-line sweep of the entire manuscript to improve clarity. We simplified abstract terms, tightened sentence structure, and improved the consistency the regime descriptions across sections. Representative changes are listed below:

1) In the **Abstract**, we revised it to

"*The cross-regional influence is organized along major transport corridors and varies non-linearly with climate. In humid forests, downwind moisture supply is highly sensitive to vegetation change, with canopy degradation associated with reduced downwind soil moisture; in semi-arid regions, initial greening is associated with increases, but further greening weakens or reverses this association*" (lines **28-32**).

2) In the **Main**, to enhance readability, we revised it to

"*Yet, despite case studies related to downwind effects*[16, 18, 19]*, we still lack a systematic framework to quantify how upwind vegetation dynamics shape soil moisture variability downwind, how these effects vary across seasons, and how they depend on climate. This gap limits our understanding of land-atmosphere feedbacks and how ecosystems sustain water availability across regions*[18-21]*.*" (lines **52-56**) and

"*Consequently, vegetation-soil moisture coupling remains largely unrepresented, outside of fully coupled climate models that often carry large uncertainties27, 28. These limitations motivate a complementary and observation-driven strategy that uses transport information to understand vegetation-soil moisture teleconnections without dependence on climate model assumptions*" (lines **73-77**).

3) In the **Upwind vegetation signals improve soil moisture prediction**, we revised

"*The transport-informed model achieves a cross-validated mean R^2 of 0.87 across grid cells and seasons (**Fig. S2**), significantly outperforming the spatial-context (0.70) and local-only (0.64) models (**Fig. 1a, b**), with improvements observed in approximately 95% of the grid cells.*" (lines **96-99**) to avoid redundant statements.

4) In the **Source-sink spatial structure of vegetation-soil moisture teleconnections**, we revised it to

"*To trace the spatial footprints of these upwind vegetation effects (**Fig. 1**), we map two complementary metrics: source influence and sink sensitivity. These metrics leverage the model's predictive attributions to capture both the directionality and non-linearity of land-atmosphere pathways, allowing us to distinguish regions that export moisture signals from those that receive them (**Fig. 2a**, **b**; **Methods**; **Figs. S9-11**). The maps reveal a predominantly positive and spatially coherent source-sink structure. The influence a cell exerts downwind and the influence it receives from upwind are, however, often unequal (**Fig. 2c**; **Methods**; **Fig. S12**).*" (lines **142-149**) and "*On average, in a*

*typical month 55% of cropland cells are LAI-dominant, i.e., the LAI-derived sink sensitivity exceeds the median across the eight input variables, indicating that upwind LAI is the leading driver of cropland soil moisture sensitivity (**Methods**). Additionally, upwind LAI anomalies account for 15% of total predictive skill, with the largest gains during the growing season (**Fig. S14**). About 60% of identified drought events coincide with positive sink sensitivity, consistent with partial moderation of severity in some cases (**Fig. S15**; **Methods** and **Supplementary Information**).*" (lines **160-164**).

5) In the section of **Climatic regimes of nonlinear vegetation-soil moisture teleconnections**, we revised

"*We group the grid cells into four regimes (R1–R4; **Fig. 3a**) based on two criteria: 1) how often the upwind influence remains positive over time, and 2) how the downwind response changes after the non-linear coupling reaches its breakpoint (**Methods**). The regime patterns are robust across a physically meaningful range of persistence thresholds (**Fig. S19**).* " (lines **211-214**) and

"*In each regime, we additionally color the response by the local ET percentile (relative to each cell's own ET distribution; **Fig. 3b–e**), which links the strength of source influence to the prevailing ET state*" (lines **217-219**) and

"*Increases in LAI are associated with nearly linear gains in downwind soil moisture once LAI anomalies rise above a low threshold (~-1.0 m^2 m^{-2}) (**Fig. 3b**); the highest source influence coincides with high-ET conditions, consistent with vigorous ET being exported downwind. R2 spans the southern Amazon-Cerrado transition and follows a similar pattern until positive LAI anomalies exceed a moderate level (~+0.5 m^2 m^{-2}), after which source influence weakens (**Fig. 3c**). Although ET remains high in this range, the source influence no longer increases, indicating that additional evapotranspired moisture is increasingly retained locally, consistent with increasing water limitation in this transitional zone.*" (lines **223-230**)

and "*R3, covering much of the La Plata basin and nearby croplands, shows a transitional response, where initial greening is associated with increased downwind soil moisture, but further greening yields diminishing returns and an eventual decline in the moisture available for atmospheric transport (**Fig. 3d**).*" (lines **234-237**) and

"*Continued forest loss via deforestation or climate-driven dieback can alter these source characteristics, reducing Amazonian moisture export, weakening continental moisture connectivity, and increasing the exposure of downwind agricultural regions to water scarcity.*" (lines **244-246**).

6) In the **Methods** section, we have introduced the three models in a progressive manner, utilizing distinct paragraphing and bulleted labels for enhanced clarity. And we have isolated the model training and running procedures into an independent subsection of "**Model training and running**". Furthermore, we have partitioned the "**Model evaluation and attribution**" section into three dedicated subsections:

“**Model performance evaluation**”, “**Predictive attribution**”, and “**Quantifying upwind dominance**”. We also revised the text to make it easily accessible to general environmental scientists.

7) In tandem with these structural changes, we have conducted a comprehensive audit of the entire manuscript and polished it for clarity, resolving any issues related to literature citations, mathematical equations, abbreviations, and grammatical fluency. The **Supplementary Information** has been thoroughly revised and updated accordingly.

Reviewer #2 (Remarks to the Author): The authors have thoroughly addressed my previous comments, and the manuscript has improved a lot. I have no further comments and support publication.

Response: We sincerely thank the reviewer for their time and for supporting publication. We greatly appreciate the constructive comments and insightful suggestions provided over the previous revision round, which have substantially helped us improve the clarity and quality of this manuscript.
